# Interaction between Theta Phase and Spike Timing-Dependent Plasticity Simulates Theta-Induced Memory Effects

**DOI:** 10.1523/ENEURO.0333-22.2023

**Published:** 2023-03-10

**Authors:** Danying Wang, George Parish, Kimron L. Shapiro, Simon Hanslmayr

**Affiliations:** 1 School for Psychology and Neuroscience and Centre for Cognitive Neuroimaging, University of Glasgow, Glasgow G12 8QQ, United Kingdom; 2 School of Psychology and Centre for Human Brain Health, University of Birmingham, Birmingham B15 2TT, United Kingdom

**Keywords:** episodic memory, STDP, theta oscillations

## Abstract

Rodent studies suggest that spike timing relative to hippocampal theta activity determines whether potentiation or depression of synapses arise. Such changes also depend on spike timing between presynaptic and postsynaptic neurons, known as spike timing-dependent plasticity (STDP). STDP, together with theta phase-dependent learning, has inspired several computational models of learning and memory. However, evidence to elucidate how these mechanisms directly link to human episodic memory is lacking. In a computational model, we modulate long-term potentiation (LTP) and long-term depression (LTD) of STDP, by opposing phases of a simulated theta rhythm. We fit parameters to a hippocampal cell culture study in which LTP and LTD were observed to occur in opposing phases of a theta rhythm. Further, we modulated two inputs by cosine waves with 0° and asynchronous phase offsets and replicate key findings in human episodic memory. Learning advantage was found for the in-phase condition, compared with the out-of-phase conditions, and was specific to theta-modulated inputs. Importantly, simulations with and without each mechanism suggest that both STDP and theta phase-dependent plasticity are necessary to replicate the findings. Together, the results indicate a role for circuit-level mechanisms, which bridge the gap between slice preparation studies and human memory.

## Significance Statement

Long-lasting changes in synaptic connectivity between neurons have been suggested to support learning and memory processes at the cellular level in the brain. Direct evidence of how this cellular mechanism links to human memory behavior is lacking. To investigate this, we constructed a computational model that implements two synaptic plasticity mechanisms that are well established in rodent studies. One mechanism suggests that strengthening or weakening in synaptic connectivity depends on the phase of ongoing theta activity. The other mechanism suggests that synaptic modification depends on spike timing between two neurons. Our model successfully reproduces results from rodent studies, as well as human episodic memory studies. These findings suggest a link between learning mechanisms at the cellular level and human associative memory.

## Introduction

Synaptic plasticity is considered to be the mechanism underlying human memory, which depends on synchronizing activity between neurons (Markram et al., 1997; Hebb, 2002). Therefore, brain oscillations that synchronize neuronal activity should be crucial for memory processes. Synchronized theta oscillatory activity (4–8 Hz), a dominant rhythm in the hippocampus (Hip), is thought to play a key role in synaptic modification (Buzsáki, 2002). Rodent studies show that the timing of inputs relative to a theta cycle are important to long-term potentiation (LTP) and long-term depression (LTD) of hippocampal synapses. *In vitro*, LTP can be induced by a brief burst of pulses delivered at the CA1 theta peak, whereas LTD occurs if the pulse is delivered at the theta trough (Huerta and Lisman, 1995). Such theta-dependent synaptic plasticity has also been observed *in vivo* in CA1 (Hölscher et al., 1997; Hyman et al., 2003) as well as in the dentate gyrus (Pavlides et al., 1988).

Several neural models have been proposed to investigate the role of hippocampal theta dynamics in learning and memory (Hasselmo et al., 2002; Norman et al., 2006; Ketz et al., 2013; Parish et al., 2018). One of these models implements theta-phase reversal between monosynaptic and trisynaptic pathways [i.e., from entorhinal cortex (EC) to CA1 via Schaffer collaterals], with the aim of promoting efficient learning by separating memory formation into phases of encoding and retrieval (Hasselmo et al., 2002; Ketz et al., 2013). The other two models purport to explore the theoretical role that theta might play in network plasticity and stability, by limiting the occurrence of LTP to a phase of high inhibition and the occurrence of LTD to a phase of low inhibition, allowing for an efficient encoding of new associations and the protection of preexisting memories from interference (Norman et al., 2006; Parish et al., 2018). The present modeling work attempts to explain the relationship more fully between theta and synaptic modification at the neuronal level, based on observations of theta-dependent plasticity in hippocampal cells (Huerta and Lisman, 1995).

Based on these findings, synchronizing inputs (i.e., arriving at the same time in the hippocampus) to the LTP inducing theta phase should lead to successful encoding of the association between those inputs, compared with when inputs are desynchronized (i.e., arrive at different times in the hippocampus). Recently, this has been demonstrated by human episodic memory studies (Clouter et al., 2017; Wang et al., 2018), in which the luminance of videos and the amplitude of sounds were modulated at 4 Hz to entrain visual and auditory cortical activity at theta frequency. Memory for synchronously presented stimuli was significantly better than for asynchronously presented stimuli. Importantly, this memory advantage was shown only for the theta-modulated stimuli, not for the faster (alpha, 10.5 Hz) or slower (delta, 1.7 Hz) frequencies (Clouter et al., 2017).

Interestingly, human studies support the idea that more effective memory formation depends on finely tuned timing between inputs. One such mechanism in synaptic plasticity is spike timing-dependent plasticity (STDP). This theoretical framework suggests that synaptic efficacy decays exponentially as a function of the delay between spikes of presynaptic and postsynaptic neurons, which leaves a very narrow time window (∼25 ms) for efficient synaptic weight changes (Song et al., 2000). If the postsynaptic neuron fires with a short delay after the firing of the presynaptic neuron, then the synaptic connection from the presynaptic neuron to the postsynaptic neuron will be strengthened. LTD of the synapses will be induced if the order of spikes is reversed. Empirical evidence of STDP has been shown in several slice and cell culture studies in rodent hippocampus (Bi and Poo, 2001) as well as in human hippocampus (Silva et al., 2010).

To better understand the role of the two components, theta phase-dependent plasticity and STDP, in human episodic memory formation, we build on a previous model, the Sync/deSync model (Parish et al., 2018), to create a new version. Significantly, we separate LTP and LTD processes, which are now modulated by opposing theta phases in the hippocampus, where previously plasticity was more generally modulated by theta phase (i.e., both LTP and LTD). To find the optimal set of parameters, we first aimed to reproduce findings from Huerta and Lisman (1995) showing that LTP and LTD occur in opposing theta phases, then used these parameters to reproduce findings from the human episodic memory studies (Clouter et al., 2017; Wang et al., 2018). Our findings provide a computational framework to link human behavior to hippocampal function at the circuitry or even cellular level.

## Materials and Methods

### Modeling principles and experimental paradigm

Inspired by models implementing reversals of theta phase across hippocampal subfields (Hasselmo, 2005; Ketz et al., 2013), we build on a previous model, the Sync/deSync model (Parish et al., 2018). This previous model focuses on the key function of CA1, where theta rhythm establishes an inhibitory phase (i.e., suppressed neural firing in CA1), during which time synapses undergo LTP, and a facilitatory phase (i.e., enhanced neural firing in CA1), when LTD occurs.

**Figure 1.**
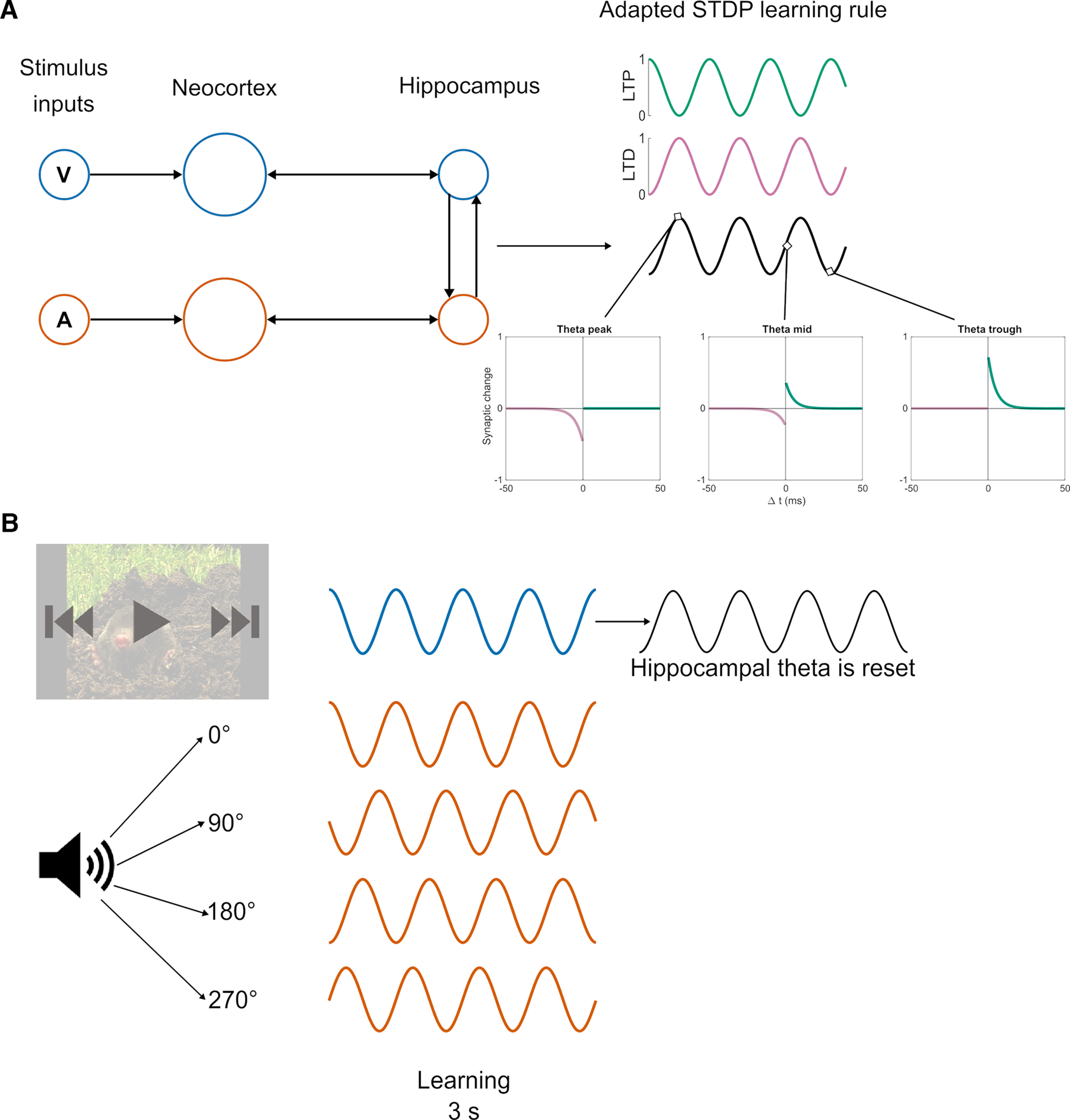
Model architecture and experimental paradigm. ***A***, Two subgroups of neurons are created to represent the visual and auditory stimuli, respectively, in both NC and the hippocampus. STDP is enabled and modulated by ongoing theta oscillations, which modulates LTP (green) and LTD (purple) at opposing phases. ***B***, To simulate the human episodic memory experiments using a multisensory entrainment paradigm (Clouter et al., 2017; Wang et al., 2018), two 4 Hz cosine waves as visual (blue) and auditory (orange) stimulus inputs were fed into two independent neural populations. The phase offsets of the auditory stimulus are either in phase (0°) or out of phase (90°, 180°, and 270°) from the visual stimulus input. Hippocampal theta phase was reset with a 180° offset from modulated visual stimulus after stimulus onset.

The model parameters were fit in relation to a hippocampal cell culture study (Huerta and Lisman, 1995) showing that stimulation at opposing theta phases induces LTP and LTD, respectively. Further, we simulated human episodic memory experiments involving a multisensory entrainment paradigm (Clouter et al., 2017; Wang et al., 2018) and showed that human episodic memory formation depends on stimulus input timing relative to theta oscillations. In these studies, the luminance of videos and the amplitude of sounds were modulated by 4 Hz cosine waves. The phase offsets of modulated sounds were either in phase (0°) or out of phase (90°, 180°, and 270°) from modulated videos (Fig. 1*B*). Each pair of sounds and videos was presented for 3 s while participants were asked to form associations between them. To test participants’ memory on the associations between the videos and sounds, each sound was presented again during recall and participants were asked to select the video that was paired with the sound earlier in the study phase. To simulate this paradigm, we fed two 4 Hz cosine waves as visual and auditory stimulus inputs into two independent neural populations. The phase offsets of the auditory stimulus are either in phase (0°) or out of phase (90°, 180°, and 270°) from the visual stimulus input.

### Neuron physiology

The model architecture is carried forward from that of the Sync/deSync model (Parish et al., 2018), which consists of two groups of neurons that represent the neocortex (NC) and hippocampus. Where previously these groups were themselves split into subgroups to denote different image-based stimuli (Parish et al., 2018), here two subgroups of neurons are created to represent the visual and auditory stimulus, respectively, in both the NC and the hippocampus (Fig. 1*A*). The total number of neurons in NC was 20, and the number of neurons in the hippocampus was 10.

Neuron membrane potential changes are modeled by an integrate-and-fire equation (Eq. 1), where the membrane potential decays over time to a resting potential (*E_L_* = –70 mV) at a rate dictated by the membrane conductance (*g* = 0.03). Here, a spike event is generated if the voltage exceeds a threshold (*V*th = –55mV), at which time the voltage is clamped to the resting potential for an absolute length of time to approximate a refractory period (2 ms). As well as the leak current, the input current for model neurons contains the sum of all spike events occurring at presynaptic neurons (*I_s_*_yn_), alternating current (AC) that represents NC alpha and hippocampal theta oscillations (*I*_AC_), any existing direct current (*I*_DC_) and an afterdepolarization (ADP) function (*I*_ADP_), described later:

(1) $$C_{m}\frac{dV_{m}}{dt}=g_{m}\left(E_{L}-V_{m}\right)+I_{\text{syn}}+I_{\text{AC}}+I_{\text{DC}}+I_{\text{ADP}}.$$

Equation 2 explains the process by which neurons communicate through spike events, whereby the sum of all spike events over time makes up the *I*_syn_ current. Here, an alpha function is used to model the EPSP, which provides an additive exponential function that diminishes further the current time point (*t*) is from the initiating spike event (*t*_fire_). The amplitude of the function is dictated by the current synaptic weight of the postsynaptic synapse (0 ≤ *ρ* ≤ 1) multiplied by its maximal weight (*W*_max_). All spike events had a delay of 2 ms before they reached *l*, as follows in Equation 2 (generation of an EPSP through time using an alpha function, here termed an EPSP function so as not to confuse with the latter modeling of alpha oscillations):

(2) $$\text{EPSP}\left(t\right)=W_{\text{max}}\cdot \rho (t)\cdot \left(e\cdot \frac{\Delta t}{\tau_{s}}\right)\cdot e^{-\frac{\Delta t}{\tau_{s}}},\Delta t=t-t_{\text{fire}}.$$

Hippocampal neurons received additional input from an ADP function, as in previous models (Jensen et al., 1996; Parish et al., 2018); Eq. 3; *A*_ADP_ = 0.2 nA, τ_ADP_ = 250 ms). This provided exponentially ramping input, which was reset after each spike event (*t*_fire_). The ADP function bestowed on model hippocampal neurons an inherent preference for spiking at a theta frequency, as well as slowing down their overall firing rate as a source of effective inhibition. It was this latter function that played a central role in the previous iteration of the Sync/deSync model (Parish et al., 2018), where the desynchronization of an oscillating cell assembly required more input for slower spiking neurons in Equation 3 (ADP function), as follows:

(3) $$I_{\text{ADP}}\left(t\right)=\frac{A_{\text{ADP}}\cdot \Delta t}{\tau_{\text{ADP}}}\cdot e^{1-\frac{\Delta t}{\tau_{\text{ADP}}}},\Delta t=t-t_{\text{fire}}.$$

**Figure 2.**
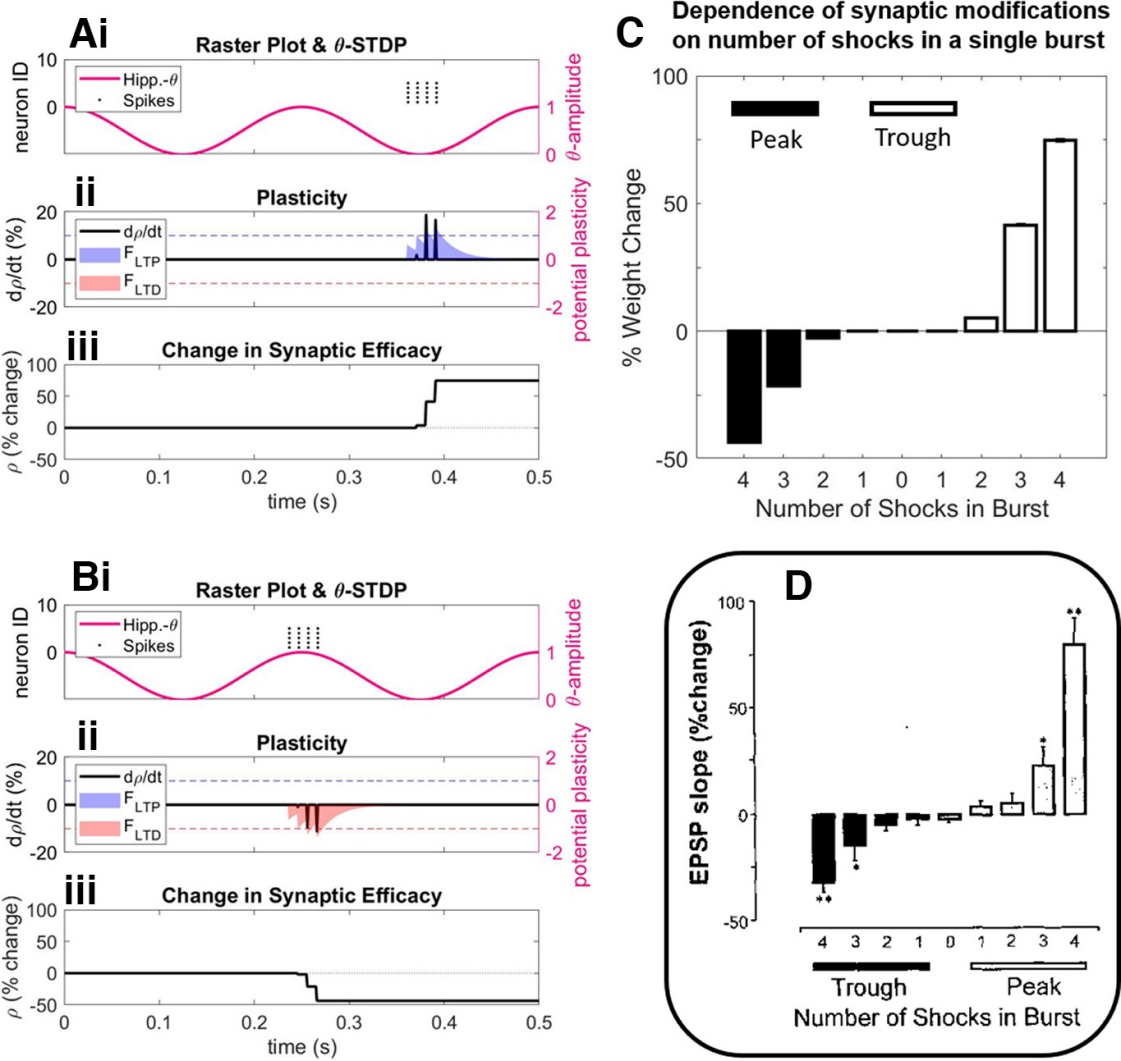
Evaluation of theta-modulated STDP. ***Ai***, ***Bi***, Independent simulations depicting synaptic plasticity after a single burst of 4 spikes at 100 Hz (amplitude = 3 pA), both in the trough (***Ai***) or the peak (***Bi***) of ongoing theta oscillations. ***Aii***, ***Bii***, Potential plasticity induced by spike pairings is calculated via functions (shaded regions; Eqs. 4.1, 4.2) for LTP (blue) and LTD (red). At the time of the spike event, synapses undergo potentiation or depression (black lines; Eqs. 5.1, 5.2) if potential plasticity is above or below a potentiation or depression threshold (blue and red dotted lines, respectively). ***Aiii***, ***Biii***, Overall synaptic change is calculated as a percentage of a baseline period through time. ***C***, Dependence on bursting for inducing plasticity, where the number of spikes in a burst was increased from 1 to 4 either in the peak or trough of ongoing theta (simulating 25 trials/condition). Overall synaptic change was calculated as the percentage difference to a baseline period, taking an average across all involved synapses. ***D***, Earlier experimental observations that indicated the importance of bursting for inducing plasticity (Huerta and Lisman, 1995). The notation of “trough” and “peak” is dependent on the location of the recording site that describes theta phase. We prefer to flip this notation in relation to prior studies, to make clear the functional role theta might play in neuronal selectivity. See also Extended Data Figure 2-1 for a more in-depth replication of the observations from the study by Huerta and Lisman (1995), in which an additional rule is implemented to model the occurrence of observed heterosynaptic plasticity on nonstimulated pathways (Extended Data Eqs. 5-1, 5-2).

The learning rule was implemented via an adapted STDP mechanism, inspired by other models (Song et al., 2000; Graupner and Brunel, 2012). We first consider two bidirectionally connected neurons in a traditional STDP framework. Upon the occurrence of a spike event in a model neuron, postsynaptic weights are strengthened for any given presynaptic neuron that spiked beforehand or weakened in the vice versa condition, the assumption being that the spike arriving at the postsynaptic connection must have either contributed to or competed with the spike event in question, depending on the directionality of the connection, leading to a reward or punishment of the synapse, respectively. To implement this, we here calculate potential synaptic plasticity via functions for LTP (*F*_LTP_) and LTD (*F*_LTD_) at the time of an eliciting spike (*t*) in Equations 4.1 and 4.2. Parameters were fitted to model the replication of data obtained from a hippocampal cell culture study (Huerta and Lisman, 1995) as shown in Figure 2, which also acts as a visual guide to the description of the set of Equations 4 and 5. Note that the measurement of global theta phase is dependent on the site of the observation, where it might be phase reversed if measuring at the hippocampal fissure or at the cell body of CA3 (Hasselmo, 2005). As in prior models (Parish et al., 2018), we reverse theta phase from that of anatomic observations (Huerta and Lisman, 1995). This enables the functional selectivity and preferential binding of neurons that fire together out of phase, at a time when most cells are inhibited and therefore inactive.

10.1523/ENEURO.0333-22.2023.f2-1 Figure 2-1 Summary of long-term synaptic plasticity during theta. ***Ai***, A single simulation depicting synaptic plasticity after two bursts of 4 spikes at 100 Hz (amplitude, 3 pA), first at the trough and then at the peak of ongoing theta oscillations. ***Aii***, ***Aiii***, Potential plasticity induced by spike pairings is calculated via functions (shaded regions; see Eqs. 4.1, 4.2) for LTP (blue) and LTD (red). At the time of spike event, synapses undergo potentiation or depression (black lines; see Eqs. 5.1, 5.2) if potential plasticity is above or below a potentiation or depression threshold (blue and red dotted lines, respectively). ***Aiv***, Overall synaptic change is calculated as a percentage of a baseline period through time. ***Aiii***, For this simulation only, we here make use of an additional equation that induces heterosynaptic LTD on nonstimulated (No Stim.) pathways, that was proportional to the amount of LTP that occurred on stimulated pathways (Stim.; see Eqs. 5.1, 5.2). ***B***, A summary of synaptic plasticity during theta, where confidence bars indicate variability over 100 trials. The percentage for LTP on stimulated pathways (Stim. LTP) and heterosynaptic LTD on unstimulated pathways (No Stim. Het. LTD) is the absolute of the percentage change of synapses relative to a baseline period. The percentage for LTD on stimulated pathways (Stim. LTD) is the depression produced by a single burst compared to the potentiation that preceded it (e.g., 100% is complete reversal). ***C***, Experimental observations (Huerta and Lisman, 1995) surmising the relationship of theta and neuronal activity.
Equation 5-1. Probability of synaptic plasticity on any *j* value of a spiking neuron where postsynaptic LTP has taken place.
Equations 5-2.1, 5-2.2. Heterosynaptic LTD (LTD_H_) acting on any *j* that has met the probability for change in Extended Data Equation 2-1. The amount of heterosynaptic LTD is further normalized against the amount of prior LTP on the postsynaptic connection [dρ(1)/dt from Eq. 6.1], such that the sum of LTD on presynaptic connections is approximately equal to half the amount of LTP that occurred on postsynaptic connections. Download Figure 2-1, TIF file.

In the case of potentiation (Eq. 4.1), potential LTP at the post-synaptic connection (*i*) is calculated as the summation of historic presynaptic spikes (*n*_pre_) that occurred before the spike event in question (where *t_i_* < *t*), weighted by an absolute value (*A*_+_ = 0.65). Contributions of presynaptic spikes were proportional to an exponential decay, thus favoring spikes that occurred close together in time (τ*_s_* = 20 ms). Potential potentiation of the synapse in response to eliciting spike events is shown in Figure 2*Aii* (right-hand axis, blue-shaded region). In the case of depression (Eq. 4.2), potential LTD at the presynaptic connection (*j*) was similarly calculated as the summation of historic postsynaptic spikes (*n*_post_) that occurred before the spike event in question (where *t_j_* < *t*), weighted by an absolute value (*A*_–_ = 0.65). Similarly, potential depression of the synapse in response to eliciting spike events is shown in Figure 2*Bii* (right-hand axis, red-shaded region), as follows:

(4.1) $$F_{\text{LTP}}\left(t,i\right)=\overset{n_{\text{pre}}}{\underset{t_{i}<t}{\sum }}A_{+}\cdot \left[1-\theta \left(t_{i}\right)\right]\cdot e^{\frac{t_{i}-t}{\tau_{s}}},$$

(4.2) $$F_{\text{LTD}}\left(t,j\right)=\overset{n_{\text{post}}}{\underset{t_{j}<t}{\sum }}A_{-}\cdot \theta \left(t_{j}\right)\cdot e^{\frac{t_{j-t}}{\tau_{s}}},$$

where synaptic plasticity functions (*F*) calculate potential plasticity as the summation of the total number (*n*_post_ and *n*_pre_) of historic spike events (*t_i_* and *t_j_*) arriving at a postsynaptic (*i*) or presynaptic (*j*) synapse relative to a given spike event (*t*), where an absolute value (*A*_+_/*A*_–_) is modulated by the difference in spike times and theta phase. Plasticity functions are separated for both LTP and LTD, which are in turn modulated by opposing phases of a theta oscillation (0 ≤ θ ≤ 1).

Further adding to this classical STDP framework, we here modulate learning by ongoing theta oscillations (0 ≤ θ ≤ 1), as in previous models (Hasselmo, 2005; Norman et al., 2006; Ketz et al., 2013; Parish et al., 2018) and as suggested by empirical evidence (Pavlides et al., 1988; Huerta and Lisman, 1995). As a continuation of previous modeling work (Parish et al., 2018), where both LTP and LTD were multiplied by the phase of theta, we then provided preferential phases of general plasticity to active neurons. Here, we develop the learning rule by splitting LTP and LTD into opposing phases of theta (as exemplified in Eqs. 4.1 and 4.2, where historic spike events were further weighted by either theta or the inverse of theta for *F*_LTP_ and *F*_LTD_, respectively, as indicated in Fig. 2*A*,*B* by the relationship of potential potentiation and depression to the phase at which spike events occur relative to an ongoing theta cycle). The current model replaces the exponential passive decay with the active theta phase-specific LTD, which was crucial for the model to replicate further experimental work (Clouter et al., 2017; Wang et al., 2018). Nuanced offsets in the phase of active stimuli were preferentially rewarded or punished because of this segregation of continuous time into phases of potentiation and depression. In this way, stimuli that synchronized in phase were rewarded and bound together, while those that dropped out of phase with one another were actively punished.

For each spike event, Equations 5.1 and 5.2 were applied to all *i* and *j* to the spiking neuron in a single-use fashion [i.e., at the millisecond of spike occurrence (*t*) and not over a sustained period]. The terms and parameters of Equations 5.1 and 5.2 were fit to model experimental observations of hippocampal cell cultures (Huerta and Lisman, 1995) and are visualized in the left-hand axis of Figure 2, *A* and *B* (black lines). Synapses changed in proportion to their prior value (or the inverse), such that 0 ≤ ρ ≤ 1. Synaptic change occurred at a constant rate for potentiation (γ*_p_* = 1.5) and depression (γ_d_ = 0.75), where potentiation occurred at twice the rate of depression to more closely resemble theta-modulated plasticity observed anatomically (Huerta and Lisman, 1995), as shown in Figure 2*D*. In these observations, the bursting of single cells was just as important in acting as a gateway to plasticity. This is captured in the usage of a Heaviside function (H[]), which provides either a 1 or a 0 dependent if potential plasticity (*F*_LTP_ or *F*_LTD_) was above a plasticity threshold (ε_LTP_ = 1 for potentiation; ε_LTD_ = 1 for depression), thus nullifying singlet or doublet spike pairings from triggering plasticity at the synapse. These thresholds are indicated by the dotted lines in Figure 2, *Aii* and *Bii* (right-hand axis; blue, ε_LTP_; red, ε_LTD_). The amount of plasticity at the synapse was also moderated by the amount by which potential plasticity was above the plasticity threshold, causing a graded plasticity effect that exponentially increases the more spike pairings are contained within a burst (Fig. 2*D*, experimental observations). These alterations to the more traditional STDP rules that informed our model (Song et al., 2000; Graupner and Brunel, 2012) allowed us to closely replicate anatomic observations that synaptic plasticity is dependent on theta phase (Fig. 2*A*,*B*) and bursting (Fig. 2*C*) in hippocampal cell cultures (Huerta and Lisman, 1995). This in turn has allowed us to replicate more nuanced memory effects (Clouter et al., 2017; Wang et al., 2018) than the previous instantiation of the Sync/deSync model (Parish et al., 2018), as follows:

(5.1) $$\frac{d\rho_{i}}{dt}=\gamma_{p}\left(1-\rho_{i}\right)\cdot H\left[F_{\text{LTP}}\left(t,i\right)-\varepsilon_{\text{LTP}}\right]\cdot \left[F_{\text{LTP}}\left(t,i\right)-\varepsilon_{\text{LTP}}\right],$$

(5.2) $$\frac{d\rho_{j}}{dt}=-\gamma_{d}\cdot \rho_{j}\cdot H\left[F_{\text{LTD}}\left(t,j\right)-\varepsilon_{\text{LTD}}\right]\cdot \left[F_{\text{LTD}}\left(t,j\right)-\varepsilon_{\text{LTD}}\right].$$

Equations 5.1 and 5.2 show actual plasticity acting on a synapse (0 ≤ ρ ≤ 1) at the occurrence of a spike event (*t*). For any given spiking neuron, postsynaptic (*i*) and presynaptic (*j*) efficacy changes are induced by potential plasticity functions (*F*_LTP_ and *F*_LTD_, respectively) being above a threshold (ε_LTP_ and ε_LTD_, respectively).

See also Extended Data Figure 2-1 for a more in-depth replication of the aforementioned anatomic observations (Huerta and Lisman, 1995). There we implement an additional rule to model the occurrence of observed heterosynaptic plasticity on nonstimulated pathways (Extended Data Eqs. 5-1, 5-2). With respect to the Occam’s razor principle, this was eventually excluded from the overall functionality of our model, as it did not directly influence results from our simulated paradigm and was also not central to the original anatomic observations. However, the additional rule might be useful to regulate network stability in the application of our theta-phase learning rule to large networks (Volgushev et al., 2016).

### NC system

Neurons within each subgroup (i.e., auditory or visual) of the NC had a 25% chance of being connected (*W*_max_ = 0.3). Connections of neurons between subgroups were not implemented in NC as it was assumed visual and auditory stimuli had not been previously associated. Synaptic plasticity (as described in Eqs. 4, 5) was also not operating on cortical synapses as in the complimentary systems framework (O’Reilly et al., 2014) it is assumed that cortical plasticity occurs on a much slower timescale. Background noise that each NC neuron is receiving was estimated by Poisson distributed spike trains (4000 spikes/s; *W*_max_ = 0.023). A cosine wave of 10 Hz (amplitude = 0.1 pA) was fed into NC neurons via *I*_AC_. Two constant inputs were fed into each NC subgroup to simulate the presentation of visual and auditory stimuli via *I*_DC_ (amplitude = 1.75 pA). These inputs were either modulated by a cosine wave at different frequencies and phase offsets or not modulated depending on the simulation purpose, which will be provided below.

### Hippocampal system

The two subgroups of hippocampal neurons that represented visual and auditory stimuli were fully connected to their NC counterparts (Fig. 1*A*; *W*_max_ = 0.35 for NC → Hip synapses; *W*_max_ = 0.08 for Hip → NC synapses), as it was assumed both stimuli were previously known. Background noise that each hippocampal neuron is receiving was estimated by Poisson distributed spike trains (1500 spikes/s; *W*_max_ = 0.015). A cosine wave of 4 Hz (amplitude = 0.25 pA) was fed into each hippocampal neuron to model ongoing theta activity. Synapses within the entire hippocampus had a probability of 50% of forming a connection (*W*_max_ = 0.65), such that weights for intrasubgroup synapses were set to maximum and those for intersubgroup synapses were initially set to 0. Synaptic plasticity was in effect on all hippocampal synapses, as described in Equations 4 and 5, allowing for the association of visual and auditory stimuli to take place within the hippocampus.

Critical to the model was the assumption of an intermediary relay node between NC and hippocampal subgroups, assumed to be located somewhere in the EC. We implemented a filter function to simplify the relay node as a filtered input, where the amplitude of the EPSP function (Eq. 2) that was applied to spikes originating in the NC and arriving at hippocampal synapses was modulated via the pathway between EC and the hippocampus (Eq. 6; *W*_EC_ = 0.3). This pathway is known to have a reversal phase relationship with hippocampal theta (Hasselmo, 2005), resulting in a stronger input at the hippocampal theta trough and a weaker input at the hippocampal theta peak. This intermediary filter allowed for model NC neurons that are entrained at a theta frequency to be active at and thus maximally induce plasticity in the appropriate hippocampal theta phase, as follows:

(6) $$\theta_{\text{EC}}=\frac{\left(1-\theta_{\text{Hip}}\right)+\left(1-W_{\text{EC}}\right)}{1+\left(1-W_{\text{EC}}\right)},$$

where theta in the EC existed as the phase reversal of hippocampal theta (0 ≤ θ_Hip_ ≤ 1), such that EC intermediary modulation of the EPSP function between NC and hippocampus neurons existed within the range 0 → 0.5 ≤ θ_EC_ ≤ 1, dependent on *W*_EC_.

### Simulation procedure and model evaluation

The human episodic memory paradigm we chose to simulate is a multisensory associative memory paradigm from the study by Clouter et al. (2017). The amplitude of neutral sound clips (3 s) and the luminance of neutral movie clips (3 s) were modulated with a 4 Hz sine wave. The phase offsets between the modulation of movies and sounds were 0° (in-phase) or 90°, 180°, and 270° (out of phase). Participants were asked to remember the association between a sound and a movie. After a distractor task, they were presented with one of the sounds and asked to choose, from four still images, which image was from the movie they had seen earlier paired with the sound. To compare our model with this episodic memory paradigm, we simultaneously fed two cosine waves (0 ≤ amplitude ≤ 1 pA) into the visual and auditory NC subgroups (Fig. 1*B*). A 2 s interstimulus interval was used before visual–auditory stimulus presentation. The two cosine waves were modulated at 4 Hz (theta), 1.652 Hz (delta), and 10.472 Hz (alpha) with auditory stimulus phase offsets of 0°, 90°, 180°, and 270° from the visual stimulus. The faster and slower frequencies were chosen to assess frequency specificity of the learning effect, as shown in the study by Clouter et al. (2017). A baseline condition was also conducted by comparing learning in the theta 0° and 180° phase offset conditions with a nonoscillatory, constant input (the “no-flicker” condition in Clouter et al., 2017). To account for the difference in the amount of information between oscillatory and nonoscillatory conditions, the stimulus length in the no-flicker condition was half of the length in the theta conditions (1.5 s). To explore the model behavior beyond our empirical data, stimulus inputs were modulated at three additional frequencies, 18.335 Hz (beta), 41.236 Hz (low gamma), and 71.771 Hz (high gamma) with four more phase offset conditions, 45°, 135°, 225°, and 315°. To allow learning in stimulus inputs modulated by higher frequencies, stimulus input strength was increased by using an exponential function of stimulus modulation frequency [1.75 · exp((frequency/20)^3^); delta stimulus strength = 1.75; theta stimulus strength = 1.76; alpha stimulus strength = 2.02] and as a logarithmic function of stimulus modulation frequency [2.2 · log10(frequency); beta = 2.78; low gamma = 3.55; high gamma = 4.08].

To model the variability of brain activity entrained by an external rhythmic stimulus, we introduced noise that was formed by a normal distribution centered at each stimulus input frequency (i.e., 4, 1.652, and 10.472 Hz) and with an Standard Deviation (SD) of 0.015 multiplied by each input frequency, respectively, for additional independent simulations. Similarly, in another set of independent simulations (theta, delta, alpha, and no-flicker conditions), we introduced noise to hippocampal dynamics by randomizing the hippocampal theta frequencies over a normal distribution centered at 4 Hz and with an SD of 0.02, as well as randomizing the EC phase offsets relative to ongoing theta with a normal distribution centered at 180° and with an SD of 0.167. To model the trial-by-trial variability of phase offsets between inputs, noise was introduced to phases of two input stimuli by randomizing the phases over normal distributions centered at the corresponding phases (i.e., 0°, 90°, 180°, and 270°) with an SD of 5. So far, for all simulations, ongoing alpha and theta cosine waves (i.e., *I*_AC_ in Eq. 1) had a random phase at the beginning of each simulation. During learning, hippocampal theta phase was reset with a 180° offset from modulated visual stimulus input after stimulus onset (Fig. 1*B*). Importantly, this enabled the fluctuation of theta-modulated plasticity to be fully synchronized with stimulus inputs, thus maximizing the learning potential of the model. Evidence for such a theta reset exists in several empirical studies (Rizzuto et al., 2003; ter Wal et al., 2021).

Model evaluation was performed by comparing the full model with two compact versions of the model. In the theta-phase learning-only version, the STDP component was eliminated, and selected synapses were strengthened or weakened depending on hippocampal theta phase (i.e., removing the exponential components and redefining −1 ≤ θ ≤ 1 in Eqs. 4.1 and 4.2, as well as forcing the constants γ*p* and –γ*d* in Equations 5.1 and 5.2 to both be positive). This forced synapses to be bidirectionally and nonspecifically weakened or strengthened at the inhibitory or excitatory phases of theta, respectively. In the STDP-only version, we removed hippocampal theta activity, such that hippocampal theta phase did not reset with stimulus onset and both STDP weight changes and EPSP at synapses of input to the hippocampus were independent of ongoing theta phase; that is, θ(*t*) was set to 1 in Equations 4.1 and 4.2 and θ_EC_ was set to 1 in Equation 6. This resulted in a lack of punishment for weak weight changes between groups with slightly overlapping time windows, a function previously performed by theta-specific LTD. This caused sufficient learning in every phase offset condition to such an extent that they did not differ from each other (Extended Data Fig. 7-1). To resolve this, we adjusted the range of input strength from between 0 and 1 to between −1 and 1 (amplitude = 1.75 pA), thus more precisely defining the time windows for overlapping firing between the two groups of neurons and punishing the weak weight changes between slightly overlapping groups, allowing us to evaluate the impact of STDP learning on the theta-modulated inputs with different phase offsets. All simulations were run for 384 trials for each condition, except the simulations for three additional input frequencies and phase offset conditions (48 trials for each condition), which were then averaged across trials for each condition. For each simulation, we randomized a new set of initial synaptic connections as well as new Poisson distributed spike trains for all conditions. We also initialized a new randomized frequency or EC phase offset for the simulations with noise.

### Model comparison statistics

**Figure 3.**
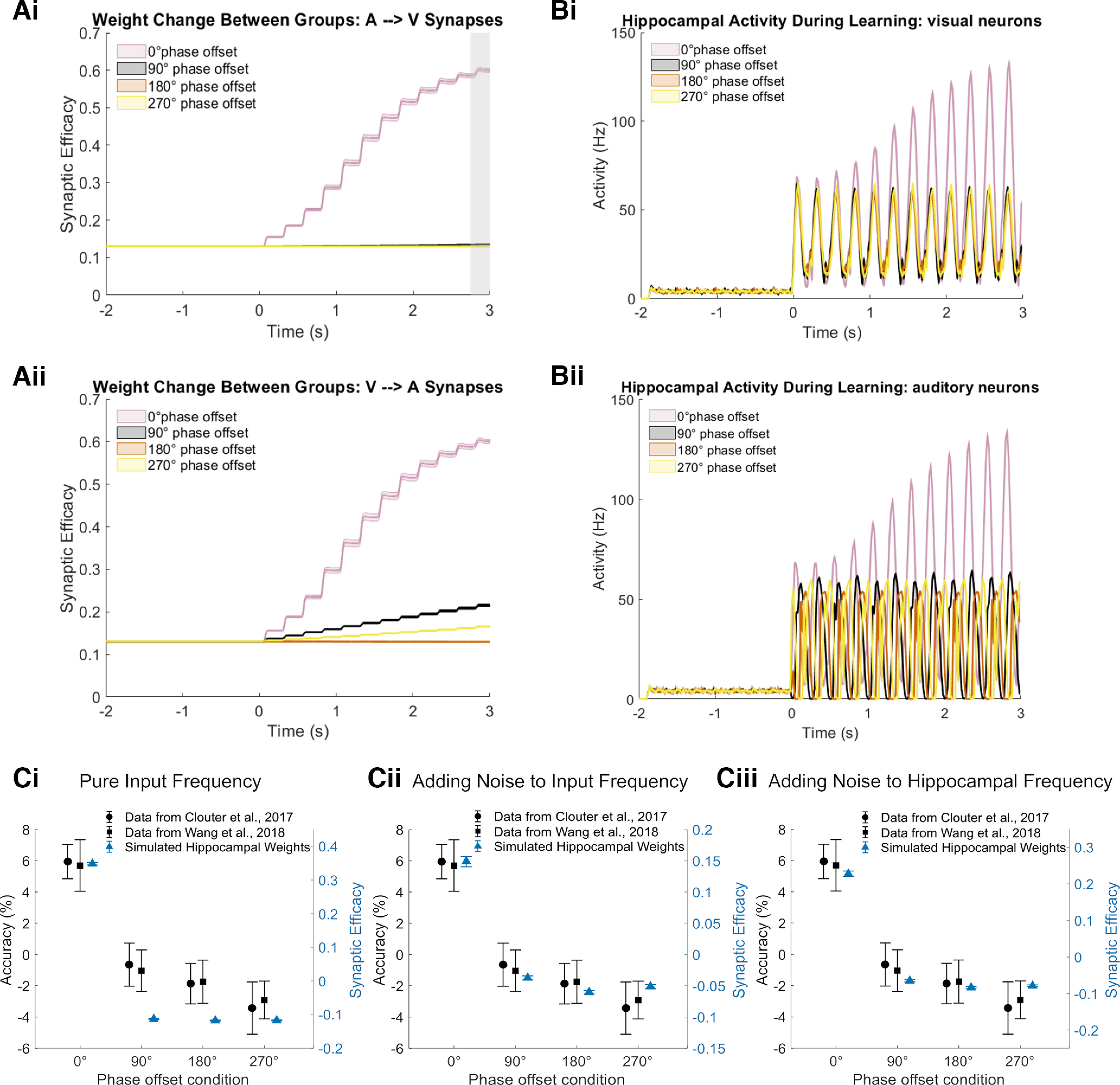
Recall performance of the model as a function of the phase offset condition when stimulus inputs are modulated at theta frequency (4 Hz). ***Ai***, Hippocampal weight change from the auditory to the visual subgroup during learning. Weights from the auditory to the visual subgroup increase significantly in the 0° phase offset condition after stimulus onset. The weights after learning are averaged between 2.75 and 3 s after stimulus onset (gray shaded area) to evaluate the recall performance of the model. ***Aii***, Same as in ***Ai***, but the weight change is from the visual to the auditory subgroup. ***Bi***, Firing rate of hippocampal visual neurons responding to the auditory stimulus during learning. ***Bii***, Same as in ***Bi***, but the firing activity is from hippocampal auditory neurons responding to the visual stimulus during learning. The phase offset conditions in both ***Ai*** and ***Bi*** represent phases of auditory (A) to visual (V), while the phase offset conditions in both ***Aii*** and ***Bii*** represent phases of V–A. Shaded error bands represent the Standard error of the mean (SE). ***C***, Mean of weights from the auditory to the visual subgroup after learning from 384 simulations and empirical data from Clouter et al. (2017) and Wang et al. (2018). Accuracy is normalized by subtracting the mean over all 4 phase offset conditions to make the between-studies data more comparable (i.e., to correct for differences in absolute memory performance between studies). ***Ci***, Simulations of pure input frequency (4 Hz), hippocampal frequency (4 Hz), and EC phase offset (180°) from hippocampal theta. ***Cii***, Simulations of input frequencies randomly drawn from normal distribution with a mean of 4 and an SD of 0.015 * 4, a pure hippocampal frequency of 4 Hz, and EC phase offset of 180° from hippocampal theta. ***Ciii***, Simulations of a pure input frequency of 4 Hz, hippocampal frequencies randomly drawn from normal distribution with a mean of 4 and an SD of 0.02, and EC phase offsets randomly drawn from normal distribution with a mean of 180° and an SD of 0.167. Error bars represent the SE. See Extended Data Figure 3-1 for the results that the hippocampal weight change is converted to the memory decision index.

The fitting of the full model was statistically compared with the fitting of the theta phase-only and the STDP-only models, respectively, using *F* tests. To directly compare the simulated memory performance with the empirical findings, after learning, hippocampal weights were averaged between 2.75 and 3 s (Fig. 3*Ai*) for each trial in the theta, delta, alpha, and no-flicker conditions. A memory decision index was computed by comparing the mean hippocampal weight value with a threshold (Extended Data Fig. 3-1). The threshold was set to the 10th percentile of the values in each trial across the above-mentioned conditions. The memory performance of a trial (recalled successfully or not) was determined by whether the value was above the threshold. The threshold was also applied to other simulations when noise was introduced to the input frequency or hippocampal theta frequency, as well as the theta phase-only and STDP-only models. The model comparisons were statistically tested by the Bayesian information criterion (BIC).

10.1523/ENEURO.0333-22.2023.f3-1 Figure 3-1 Simulated memory decision index. ***Ai–Aiii***, Simulated memory decision index and empirical data from the studies by Clouter et al. (2017) and Wang et al. (2018). Accuracy is normalized by subtracting the mean over all 4 phase offset conditions to make the between-studies data more comparable. ***Ai***, Simulations of pure input frequency of 4 Hz, hippocampal frequency of 4 Hz, and EC phase offset of 180° from hippocampal theta. ***Aii***, Simulations of input frequencies randomly drawn from normal distribution with a mean of 4 and an SD of 0.015 * 4, pure hippocampal frequency of 4 Hz, and EC phase offset of 180° from hippocampal theta. ***Aiii***, Simulations of pure input frequency of 4 Hz, hippocampal frequencies randomly drawn from normal distribution with a mean of 4 and an SD of 0.02, and EC phase offsets randomly drawn from normal distribution with a mean of 180° and an SD 0.167. Error bars represent the SE. ***Bi***, Simulations of pure input frequencies, hippocampal frequency of 4 Hz, and EC phase offset of 180° from hippocampal theta. ***Bii***, ***Biii***, Simulations of pure input frequency of 4 Hz, hippocampal frequencies randomly drawn from normal distribution with a mean of 4 and an SD of 0.02, and EC phase offsets randomly drawn from normal distribution with a mean of 180° and an SD 0.167. All error bars represent the SE. ***C***, ***D***, Model comparisons in simulated memory decision index between the full model and two alternative versions. ***Ci***, ***Cii***, Simulated memory decision index of two versions of model was fit to the data from the study by Clouter et al. (2017). ***Ci***, The full model was compared with a theta-phase learning-only version of the model. ***Cii***, Same as ***Ci***, but the comparison was between the full model and an STDP-only version of the model. ***D***, Same as ***C***, but the simulated memory decision index of two versions of the model was fit to the data from the study by Wang et al. (2018). All simulations were performed with pure input frequency of 4 Hz, hippocampal frequency of 4 Hz, and EC phase offset of 180° from hippocampal theta. All error bars represent the SE. The model comparisons were statistically tested by the BIC scores. The BIC score difference between the full model and theta phase-only model is 0.72 for fitting to the data from the study by Clouter et al. (2017), and 0.54 for fitting to the data from the study by Wang et al. (2018). The BIC score difference between the full model and STDP-only model is 0.03 for fitting to the data from the study by Clouter et al. (2017), and –0.38 for fitting to the data from the study by Wang et al. (2018). The BIC scores do not suggest that full model fits better to the data. This could be due to the fact that BIC scores penalized the model with more parameters to a greater degree. The pattern of the full model of the simulated memory decision index resembles the empirical data. Download Figure 3-1, EPS file.

### Data availability

All human data and the MATLAB code of the model to run the simulations can be downloaded at: https://github.com/GP2789/Sync-deSync-model-TIME. The code is available as Extended Data 1. The results of the simulations were obtained by running the code using a PC with a 2.8 GHz processor and 32 GB of RAM, a 64 bit version of the Microsoft Windows 10 operating system, and 64 bit MATLAB version R2020a.

10.1523/ENEURO.0333-22.2023.ed1 Extended Data 1 Human data and the MATLAB code of the model to run the simulations., Download

## Results

### Simulated hippocampal weight change reproduces theta-phase synchrony-induced memory enhancement

We compared memory performance between the results of our model and those from human episodic memory studies that establish modulation of theta-phase synchrony. Specifically, Clouter et al. (2017) and Wang et al. (2018) used a multisensory theta entrainment paradigm to externally manipulate the precise timing of visual and auditory inputs. As depicted in Figure 1*B*, luminance and amplitude of visual and auditory stimuli were modulated at 4 Hz with different phase offsets between the two stimuli [0° (in-phase), and 90°, 180°, and 270° (out-of-phase)]. Human participants’ recall accuracy was evaluated by presenting the auditory stimulus and requesting participants to select the associated visual stimulus with which it was previously paired during the encoding phase. The encoding phase was simulated by feeding two 4 Hz cosine waves that had phase offsets at either in-phase (0°) or out-of-phase (90°, 180°, and 270°) for 3 s into two subgroups of NC neurons that represented visual and auditory cortical neurons. The hippocampal theta phase was reset to be 180° offset from the visual stimulus by stimulus onset during encoding so that the LTP phase was aligned with the visual stimulus input. The entire procedure was simulated 384 times for each phase offset condition, and the results were then averaged across simulation runs per condition.

To evaluate the recall performance of the model, the hippocampal weights from the auditory to the visual subgroup after learning were averaged between 2.75 and 3 s (one theta cycle) after stimulus onset. As shown in Figure 3*Ai*, weights after stimulus onset increase significantly in the 0° phase offset condition compared with all other (out-of-phase) conditions. In the 0° phase offset condition, increases in weights are rhythmic at 4 Hz. This is because both inputs are synchronized with the inhibitory phase of theta. Neurons of both groups rarely fire at the excitatory phase of theta (i.e., the LTD-inducing phase); therefore, weight changes are mainly positive until weights reach their maximum. This is also reflected by the hippocampal firing activity during learning for the group of neurons corresponding to visual inputs. After stimulus onset, an increase in firing of the visual neurons in the 0° phase offset condition is evident across time because of the weight change (Fig. 3*Bi*), which leads to changes in the input current for *I*_syn_ (see Materials and Methods).

Figure 3*Ci* shows mean weights simulated for each phase offset condition compared with experimental data from Clouter et al. (2017) and Wang et al. (2018). Consistent with both experimental datasets, the model shows the highest memory performance for the 0° phase offset condition relative to the other three out-of-phase conditions. Moreover, the model successfully replicates the previously observed pattern that memory performance in the out-of-phase conditions (90°, 180°, and 270°) did not differ from each other. The fact that the recall performance in the 90° and 270° phase offset conditions does not differ from the performance in the 180° phase offset condition could be caused by the interaction between theta phase-dependent and STDP learning. Inputs of auditory neurons on visual neurons in the 90° and 270° phase offset conditions are less synchronized with the LTP phase within a theta cycle; thus, the synaptic changes are weighted less by corresponding theta phases. As synaptic weight changes decay exponentially across time, the resultant small weight changes will decay drastically when the next visual stimulation peak induces substantial spiking events, given that the delay between auditory and visual stimulation peak is >50 ms.

We note the difference, however, between the modeled and empirical data, whereby the model shows relatively larger differences between the in-phase and out-of-phase conditions compared with the experimental data. This could be because this version of the model had zero noise (i.e., it assumed a perfect transmission of rhythmic activity from the sensory channels to the brain as well as a perfect alignment of the hippocampal theta rhythm to the sensory input). Both are unlikely to be the case in the human brain, which is expected to show trial-by-trial variability to rhythmic sensory input (Wang et al., 2018). To model the variability of sensory-transmitted rhythms, we generated a normal distribution that centered at 4 Hz, with an SD of 0.015 multiplied by 4 as noisy input frequencies (see Materials and Methods). Incorporating such noise sources led to a better fit of the simulated data to the empirical data (Fig. 3*Cii*). Additionally, we modeled the variability of hippocampal theta by varying theta frequency (i.e., using a normal distribution centered at 4 Hz with an SD of 0.02). In addition, the EC phase offsets relative to ongoing theta was also randomized with a normal distribution centered at 180° with an SD of 0.167. Again, the simulation results showed a pattern more similar to that observed in the empirical studies (Fig. 3*Ciii*). The patterns of simulated memory decision index are similar to the pattern of mean weights (Extended Data Fig. 3-1*A*).

A prediction made by our model is that the theta synchronization-induced learning effect is bidirectional. As shown in Figure 3, *Aii* and *Bii*, weights from neurons of the visual subgroup to neurons of the auditory subgroup also increase significantly in the 0° phase offset condition compared with the out-of-phase conditions, as does hippocampal firing activity during learning for the neurons of the auditory subgroup. Although small weight increases are shown in the 90° and 270° phase offset conditions, the weights are very low compared with the 0° condition. The small increases might be because the visual stimulus is always in phase with the inhibitory phase of theta. Therefore, the weight change from the visual neurons might be able to be accumulated if firing happens. This prediction could be tested in a human behavioral experiment where memory is cued with the visual stimulus (i.e., the paired auditory stimulus needs to be recalled).

**Figure 4.**
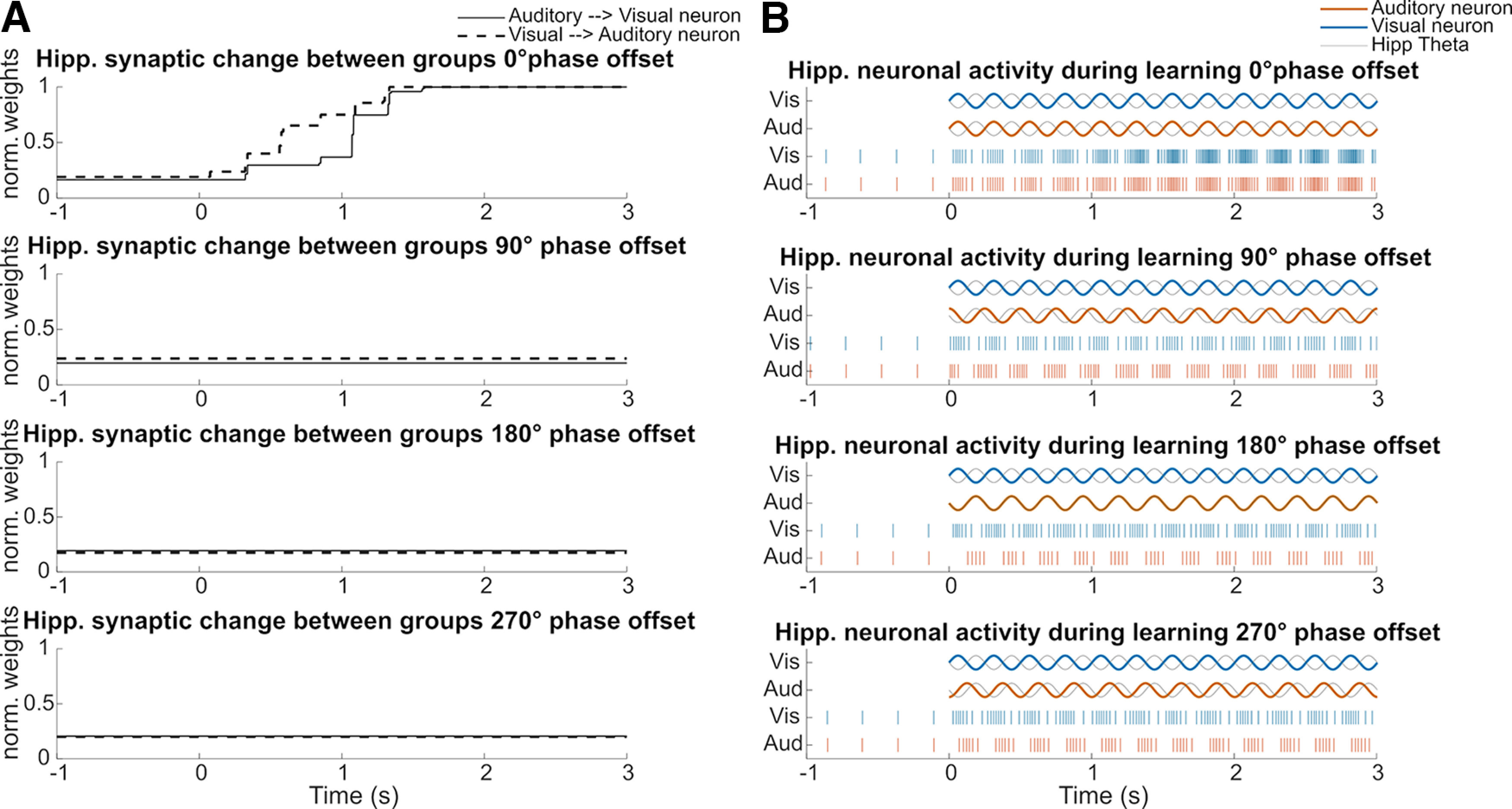
Hippocampal weight change between groups and firing activity for single hippocampal neurons in each phase offset condition at theta frequency (4 Hz). ***A***, Hippocampal weight change between a random selected neuron of the auditory subgroup and a neuron in the visual subgroup during learning. The neurons are bidirectionally connected. The solid black line represents the weight change from the auditory neuron to the visual neuron. The dashed black line represents the weight change from the visual neuron to the auditory neuron. ***B***, In each phase offset condition, the first two line plots show the stimulus inputs and the ongoing hippocampal theta oscillation during learning. Raster plots represent the firing activity of the same neurons shown in ***A***, in each phase offset condition, respectively. See also Extended Data Figure 4-1 for the hippocampal weight change between groups and firing activity for single units in the delta and alpha frequency-modulated conditions.

Figure 4*A* shows synaptic weight changes between randomly selected auditory neurons and their connected visual neurons in each phase offset condition during learning. Weights in the 0° phase offset condition increase bidirectionally. As shown in Figure 4*B*, both auditory and visual neurons receive corresponding inputs at the inhibitory theta phase in the 0° phase offset condition. Raster plots show the spike events of the same neurons in Figure 4*A*, which demonstrates that both auditory and visual neurons become more activated over time in the 0° phase offset condition because of increased synaptic weights.

10.1523/ENEURO.0333-22.2023.f4-1 Figure 4-1 Hippocampal weight change between groups and firing activity for single hippocampal neurons in each phase offset condition at other frequencies. ***Ai***, Hippocampal weight change between a random selected neuron of the auditory subgroup and a neuron in the visual subgroup during learning, when stimulus inputs are modulated at delta frequency. ***Aii***, In each phase offset condition at delta frequency, the first two line plots show the stimulus inputs and hippocampal ongoing theta oscillation during learning. Raster plots represent firing activity of the same neurons shown in ***Ai***, in each phase offset condition, respectively. ***Bi***, ***Bii***, Same as in ***Ai*** and ***Aii***, but stimulus inputs are modulated at alpha frequency. Download Figure 4-1, EPS file.

### Simulated hippocampal weight change reproduces theta specificity of the phase synchrony-induced memory enhancement

**Figure 5.**
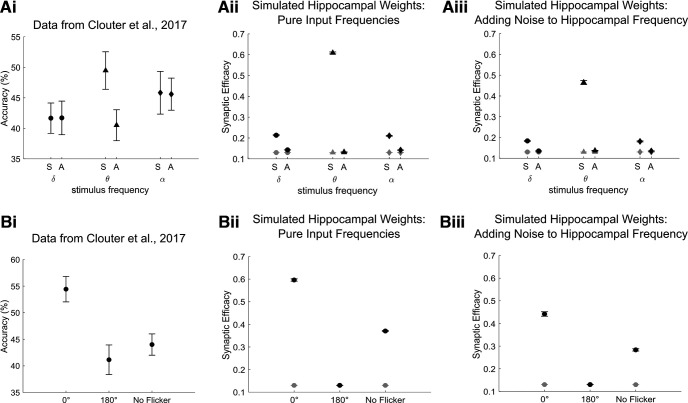
Recall performance as a function of the degree of phase synchronization in the theta-modulated condition and other control conditions. ***Ai***, Data from Clouter et al. (2017) showing recall accuracy when the movies and sounds were flickering in synchrony (S) or out of synchrony (A) at delta, theta, and alpha frequencies. ***Aii***, ***Aiii***, Mean of weights from the auditory to the visual subgroup after learning from 384 simulations. ***Aii***, Simulations of pure input frequencies (delta, 1.7 Hz; theta, 4 Hz; alpha, 10.5 Hz); hippocampal frequency, 4 Hz; and EC phase offset, 180° from hippocampal theta. ***Aiii***, Simulations of pure input frequency, 4 Hz, hippocampal frequencies randomly drawn from normal distribution with a mean of 4 and an SD of 0.02, and EC phase offsets randomly drawn from normal distribution with a mean of 180° and an SD of 0.167. Stimulus input strength increases as an exponential function of stimulus modulation frequency for lower frequencies (see Materials and Methods), delta (1.65 Hz stimulus strength, 1.75), theta (4 Hz stimulus strength, 1.76), and alpha (10.47 Hz stimulus strength, 2.02). ***Bi***, Data from the study by Clouter et al. (2017) showing recall accuracy when the movies and sounds were presented at 0° and 180° phase offsets or were unmodulated. ***Bii***, ***Biii***, Mean of weights from the auditory to the visual subgroup after learning from 384 simulations. ***Bii***, Simulations of pure input frequencies, hippocampal frequency of 4 Hz, and EC phase offset of 180° from hippocampal theta. ***Bii***, Simulations of pure input frequency of 4 Hz, hippocampal frequencies randomly drawn from normal distribution with a mean of 4 and an SD of 0.02, and EC phase offsets randomly drawn from normal distribution with a mean of 180° and an SD of 0.167. All error bars represent the SE. Black dots represent hippocampal weights after learning. Gray dots represent hippocampal weights averaged between −1.75 and 0 s during prestimulus baseline. See Extended Data Figure 5-1 for simulations of pure input and hippocampal frequencies, but noise was introduced to the phase offsets between two input stimuli.

We tested whether our model would also show theta specificity of the phase offset effects, as revealed in the study by Clouter et al. (2017). More specifically, Clouter et al. (2017) showed that the difference in memory performance between in-phase (0°) and out-of-phase (90°, 180°, 270°) conditions is specific to theta stimulation and is not observed for a slower (1.7 Hz) or faster (10.5 Hz) frequency. We therefore fed two cosine waves with the same phase offsets as above, but modulated their frequencies at 1.7 Hz (delta) and 10.5 Hz (alpha) instead of at 4 Hz (theta). This was done to prevent the chosen frequency bands occurring in a harmonic relationship with 4 Hz (Pletzer et al., 2010). To compare these results with the data from the study by Clouter et al. (2017), we averaged simulated hippocampal weights after encoding across the three out-of-phase conditions (90°, 180°, and 270°) to yield a single asynchronous measure (Fig. 5*Aii*). The modeled results replicated the pattern observed by Clouter et al. (2017) in showing that the memory difference between synchronous and asynchronous conditions is significantly greater at theta compared with the other two frequencies (Fig. 5*Ai*,*Aii*). Moreover, the model replicates the findings of Clouter et al. (2017) in showing that memory performance in the synchronous condition at theta is better than in the same conditions of the two control frequencies. This suggests that synchronous stimulation improves memory specifically in the theta frequency, reflecting coordinated timing of external inputs relative to hippocampal theta. Given that the alpha-modulated and delta-modulated inputs do not coincide with the LTP phase during learning, firing could lead to LTP or LTD, or, alternatively, half-weighted LTP and LTD (Fig. 1*A*, Extended Data Fig. 4-1, network connectivity and activity dynamics between single units in the delta and alpha frequency-modulated conditions). Therefore, only synchronization at the preferred frequency of the hippocampus is more likely to induce effective associative learning.

10.1523/ENEURO.0333-22.2023.f5-1 Figure 5-1 Weight changes as a function of the degree of phase synchronization in the theta-modulated condition and other control conditions. Simulations of pure input frequency of 4 Hz, hippocampal frequency of 4 Hz, and EC phase offset of 180° from hippocampal theta. Input phases randomly drawn from normal distributions with means of 0°, 90°, 180°, and 270°, and an SD of 5. All error bars represent the SE. Black dots represent hippocampal weights after learning. Gray dots represent hippocampal weights averaged between –1.75 and 0 s during the prestimulus baseline. Download Figure 5-1, EPS file.

**Figure 6.**
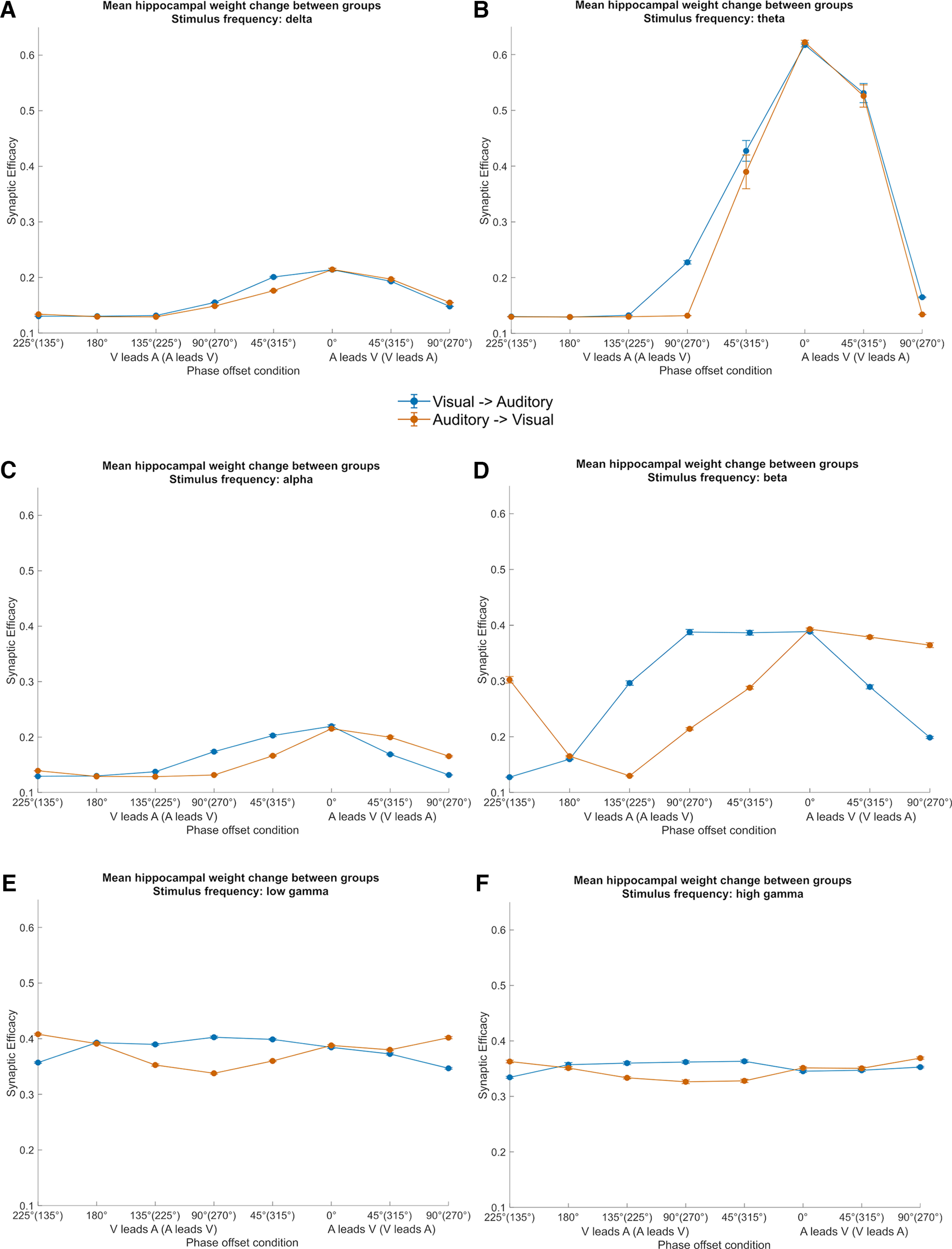
Mean hippocampal weight change between groups as a function of phase offset condition at ***A***, delta frequency; ***B***, theta frequency; ***C***, alpha frequency; ***D***, beta frequency; ***E***, low gamma frequency; and ***F***, high gamma frequency. After learning, weights were averaged across 48 simulations and between 2.75 and 3 s after stimulus onset. Stimulus input strength increases as an exponential function of stimulus modulation frequency for lower frequencies (see Materials and Methods) delta (1.65 Hz stimulus strength, 1.75), theta (4 Hz stimulus strength, 1.76), and alpha (10.47 Hz stimulus strength, 2.02). Stimulus input strength increases as a logarithmic function of stimulus modulation frequency for higher frequencies (see Materials and Methods) beta (18.34 Hz stimulus strength, 2.78), low gamma (41.24 Hz stimulus strength, 3.55), and high gamma (71.77 Hz stimulus strength, 4.08). Error bars represent the SE.

Next, we modulated the stimulus inputs at more frequencies (beta, 18.335 Hz; low gamma, 41.236 Hz; high gamma, 71.771 Hz) with more phase offset conditions (45°, 135°, 225°, 315°), which allows us to explore the learning behavior of the model as a function of stimulus frequency and phase offset condition. This enhances our understanding of the interaction of the theta phase-dependent and the STDP learning mechanisms more comprehensively. The results of this simulation are shown in Figure 6. In the theta frequency-modulated conditions, learning in the 45° and 315° phase offset conditions is benefited because of stimulation closer to the theta inhibitory phase. This learning advantage is bidirectional. Learning in the phase offset conditions that are between 90° and 270° was very low because of less synchronous stimulation relative to the LTP phase, as well as the exponential decay of weights because of the STDP learning rule. The learning advantage of the synchronous stimulation condition, compared with the asynchronous conditions is specific to theta-modulated inputs. As input modulation frequencies get faster, learning increases somewhat in asynchronous conditions (e.g., 45° or 90° phase offset conditions) as the delays between presynaptic and postsynaptic neurons firing are within the optimal time window for STDP. Moreover, weights from auditory neurons to visual neurons are higher when the auditory stimulus leads over the visual stimulus, whereas the pattern is reversed when the visual stimulus leads over the auditory stimulus. This effect is particularly pronounced in higher stimulation frequencies (>10 Hz), which is consistent with the STDP learning rule (Markram et al., 1997; Bi and Poo, 1998). However, because of the role of theta phase-dependent plasticity, learning in any other frequency than 4 Hz could not reach the maximum, as happened in the theta synchronous condition.

Our model also replicates findings from another control experiment of Clouter et al. (2017) showing that flickering visual and auditory stimuli at 4 Hz in synchrony boosts memory beyond a natural nonflickering condition (Fig. 4*Bi*). To simulate this, two constant stimulus inputs were fed into NC auditory and visual neurons during encoding. The length of the input stimuli was reduced to 1.5 s to control for the amount of overall stimulus presentation time in the flickering condition (i.e., the screen was essentially blank for half of the time because of the flicker). Figure 5*Bi* reveals that simulated hippocampal weights after learning are higher in the 0° phase offset condition than in either the 180° phase offset condition or the nonflickering condition. Because of the role of EC modulation in filtering NC inputs to be more active in the theta LTP phase and less active in the theta LTD phase (see Materials and Methods), the constant inputs result in substantial associative learning compared with the baseline. However, they are still less optimal than the theta synchronized inputs, which is consistent with the empirical data, confirming that the theta synchrony-enhanced memory effect is indeed a result of maximally optimizing input timing relative to the hippocampal theta oscillation. Figure 5, *Aiii* and *Biii*, shows that the patterns still hold after adding noise into hippocampal dynamics and input phases. Moreover, the patterns resemble the empirical data better than before adding noise, which is reflected in a smaller difference between the theta synchronous condition and the other conditions. The patterns are also replicated by a simulated memory decision index (Extended Data Fig. 3-1*B*), although performance is zero in the out-of-phase conditions. This is because of the fact that a threshold was set by the top 10% of values across trials in all conditions.

### Theta phase-dependent and STDP learning rules are necessary to reproduce the theta-induced memory effect

**Figure 7.**
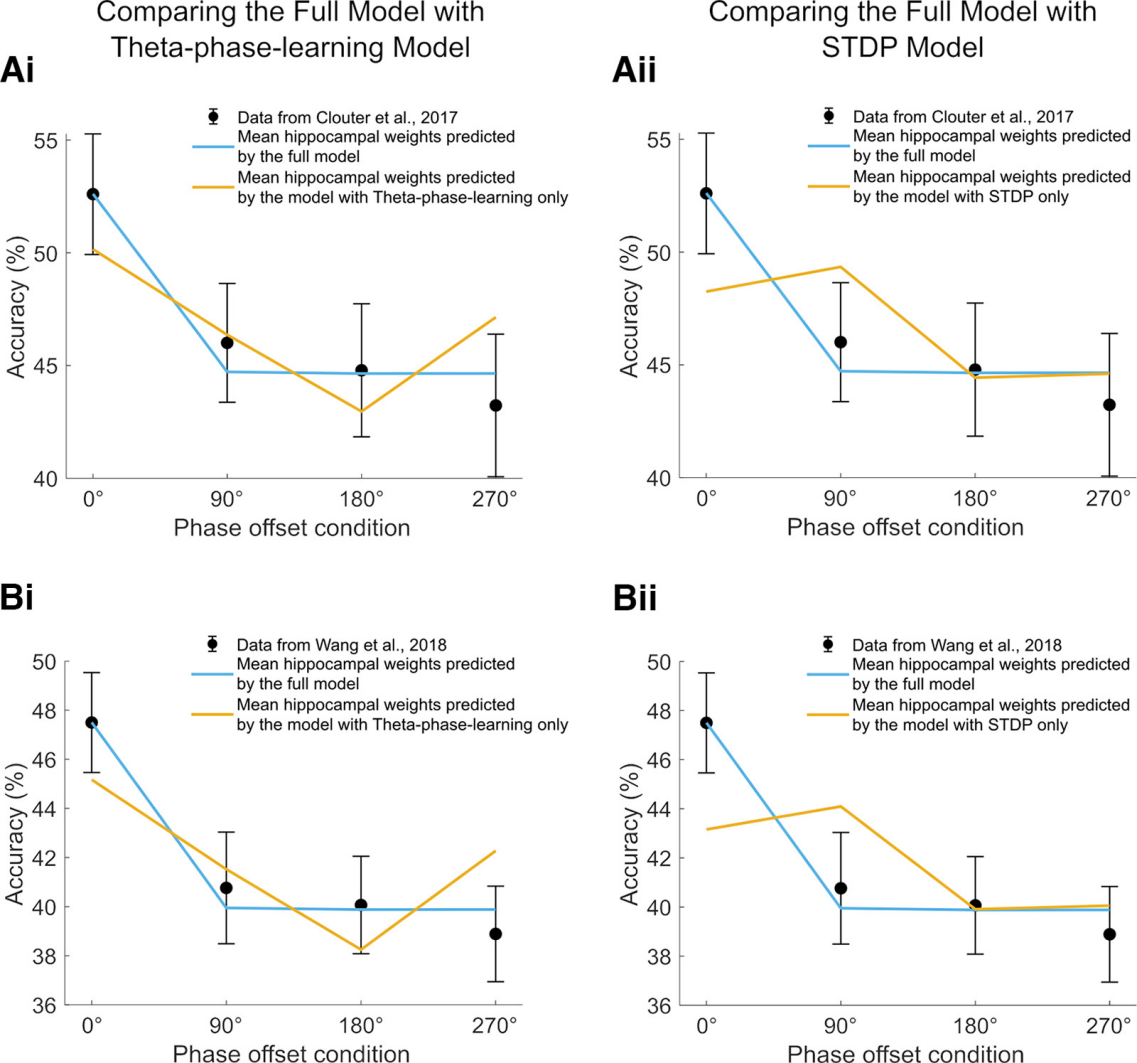
Model comparisons in recall performance between the full model and two alternative versions. ***A***, Mean of weights from the auditory to the visual subgroup after learning simulated by two versions of the model was fit to the data from the study by Clouter et al. (2017). ***Ai***, The full model was compared with a theta-phase learning-only version of the model. ***Aii***, Same as ***Ai***, but the comparison was between the full model and an STDP-only version of the model. ***B***, Same as ***A***, but the mean of weights from the auditory to the visual subgroup after learning simulated by two versions of model was fit to the data from the study by Wang et al. (2018). All simulations were done with pure input frequency of 4 Hz, hippocampal frequency of 4 Hz, and EC phase offset of 180° from hippocampal theta. All error bars represent the SE. See Extended Data Figure 7-1 for the model comparisons between the full model and the STDP-only model when the range of input strength was between 0 and 1.

We compared the recall performance of the model to two alternative versions. The first is a model of purely theta phase-dependent learning. For this version, we eliminated the STDP component such that synaptic weights are modified solely depending on hippocampal theta phase. For example, if a hippocampal neuron from the auditory subgroup fires during the inhibitory (i.e., LTP-inducing) phase, synapses between this neuron and its connected visual neurons will be potentiated, whereas the synapses between the neuron and its connected visual neurons will be depressed if it fires during the excitatory phase (i.e., LTD-inducing phase). Hippocampal weights for the four phase offset conditions were fit to the two empirical datasets (Clouter et al., 2017; Wang et al., 2018) using linear least squares. Figure 7, *Ai* and *Bi*, shows that the theta phase-dependent learning-only model does not fit the empirical data as well as the full model. The aforementioned result is likely because of the fact that memory performance for the theta phase-only model increases depending on the degree to which the inputs overlapped with theta LTP phase. Weights in the 90° and 270° phase offset conditions are therefore slightly better than in the 180° phase offset condition but is worse than in the 0° phase offset condition. We conducted *F* tests to statistically compare the fitting of the theta phase-only model with the fitting of the full model. The residual sum of squares (RSS) for the theta-only version was significantly larger than the RSS for the full model fitting to Clouter et al. (2017) theta-phase learning-only versus full (*F*_(1,3)_ = 17.11, *p *<* *0.05) and the fitting to Wang et al. (2018) theta-phase learning-only versus full (*F*_(1,3)_ = 33.90, *p* < 0.05). We suggest that this outcome arises because learning is canceled in the 180° phase offset condition, as the auditory inputs cause firing at theta LTD phase, whereas firing at 90° and 270° lead only to a reduction in the positive weights, compared with firing exactly at theta LTP phase (i.e., 0°).

10.1523/ENEURO.0333-22.2023.f7-1 Figure 7-1 Model comparisons in recall performance between the full model and the STDP-only model. ***A***, Mean of weights from the auditory to the visual subgroup after learning simulated by two versions of the model was fit to the data from the study by Clouter et al. (2017). The full model was compared with an STDP-only version of the model, in which the range of input strength was between 0 and 1. ***B***, Same as ***A***, but the mean of weights from the auditory to the visual subgroup after learning simulated by two versions of model was fit to the data from the study by Wang et al. (2018). All error bars represent the SE. Download Figure 7-1, EPS file.

Next, we created a version of the model (i.e., the STDP-only model), where all elements relating to theta-modulated plasticity were removed. In this version of the model, weight changes can occur at any time, completely independent of theta phase, if spike timing between a sending neuron and a receiving neuron is within a short time window (<50 ms). This means that if enough spikes overlap in time between inputs, learning strength will not differ between phase offset conditions. To enlarge the spike-timing gap between two inputs in each phase offset condition so the impact of STDP alone can be evaluated, we adjusted the amplitude of the 4 Hz cosine wave to be between −1 and 1 (as opposed to 0 and 1; Extended Data Fig. 7-1), which effectively narrows the time window for spiking. This leads to the largest gap in firing between two groups of neurons in the 180° phase offset condition because there is no overlap in spiking between the two groups. A few spikes overlapped in the 90° and 270° phase offset conditions, but the difference is that the spikes in the auditory group of the 90° condition are leading over the spikes in the visual group, whereas in the 270° condition, the neurons of auditory group firing are lagging behind those of the visual group. Therefore, LTP in the synapses from auditory to visual groups is expected to occur in the 90° phase offset condition. In contrast, LTD should mainly occur in the synapses from auditory to visual groups in the 180° and 270° conditions. This is confirmed by Figure 7, *Aii* and *Bii*, which shows enhanced memory performance in the 90° phase offset condition, whereas recall performance decreases exponentially from the 90° phase offset condition to the 180° and 270° phase offset conditions, with memory performance not being different between 180° and 270° phase offset conditions. However, memory performance is also enhanced for the 0° phase offset condition, where memory performance appears slightly lower compared with the 90° condition. This might be because of the complete overlap in neurons firing between two groups in the 0° condition. Since theta phase does not modulate weight changes anymore, such complete overlapping would lead to a reward and punishment simultaneously, hence balancing at a similar level in weights as in the 90° condition. As a result, the model with STDP learning mechanisms produces only a poor fit to the empirical data compared with the full model. *F* tests showed that RSS for the STDP-only version is significantly larger than the RSS for the full model [fitting to Clouter et al. (2017), STDP only (input range, −1 and 1) versus full: *F*_(1,3)_ = 23.08, *p *<* *0.05; fitting to Wang et al. (2018), STDP only (input range −1 and 1) versus full: *F*_(1,3)_ = 52.69, *p *<* *0.05). Therefore, implementing both STDP and theta phase-dependent learning mechanisms is essential to replicate the theta-induced memory effect from human episodic memory experiments.

## Discussion

We present a simple model involving a hippocampal system that implements STDP learning, where LTP and LTD are modulated by opposing phases of an ongoing theta oscillation. The model reproduces important findings from a hippocampal cell culture study showing that a brief burst of a few spikes at opposing theta phases induces LTP and LTD, respectively (Huerta and Lisman, 1995). Using parameters fitted to reproduce these rodent data, we can replicate several key findings from human episodic memory studies (Clouter et al., 2017; Wang et al., 2018). The simulated hippocampus received inputs from two different groups of neocortical neurons, visual and auditory neurons that are modulated at the same frequency as the hippocampal theta frequency. Synchronizing the inputs to be in phase with the fluctuation of theta phase-dependent LTP induces more effective hippocampal synaptic connectivity, compared with when the inputs are desynchronized. Such learning effects induced by phase synchronization were present only when the inputs were modulated at the same frequency as hippocampal theta but not at alpha or delta frequencies that do not modulate hippocampal LTP or LTD. Moreover, our model replicates the result that synchronizing input at theta frequency improved memory compared with unmodulated input, suggesting that the theta phase-induced memory effect is highly attributed to the hippocampal dynamics rather than purely perceptual binding (Clouter et al., 2017). To simulate the exact pattern of recall accuracy observed in the human episodic memory studies, the hippocampus must have both synaptic modification mechanisms. On the one hand, the theta phase-dependent learning-only model fails to replicate the pattern that learning in the 90°, 270°, and 180° conditions did not differ from each other, as these conditions seemingly benefit from the additional subtlety that STDP brings with regard to slight differences in spike timing. On the other hand, STDP learning alone shows that learning only depends on presynaptic and postsynaptic inputs timing. Therefore, both the 0° and 90° phase offset conditions show enhanced learning, compared with the 180° and 270° conditions, hence failing to reproduce the empirical findings.

Theta phase-dependent plasticity as implemented in our model separately modulates the two components of STDP: LTP and LTD. Such modulation by opposing theta phases is inspired by several theta learning models. Hasselmo et al. (2002) and Ketz et al. (2013) modeled the theta phase reversal between hippocampal subregions and demonstrated its functional utility in memory formation. In the model of Hasselmo et al. (2002), phase differences between EC and hippocampal CA1 either encourages LTP within hippocampus CA3 to CA1 in an effective encoding phase or blocks this activation pathway in favor of encouraging cortical activation through the reverse pathways in an effective retrieval phase. Thus, theta phase reversal efficiently separates encoding and retrieval, enabling pattern separation of overlapping memories. Ketz et al. (2013) modeled similar hippocampal theta dynamics, additionally showing that theta-phase learning is more effective for error-driven learning compared with purely Hebbian learning, thus showing how theta can further hone accurate memory formation in an iterative manner. Another model in this line of thought operates at a more theoretical level with the aim of solving some issues arising from competing and overlapping representations in neural network learning (Norman et al., 2006). Rather than modulating specific neuronal pathways, the model capitalizes on the general function of oscillations in rhythmically varying levels of inhibition. The model of Norman et al. (2006) is able to solve competition between overlapping memories by showing how an increase in inhibition can strengthen target memories, while a decrease in inhibition can weaken competitors. Thus, theta, which Norman et al. (2006) hypothesize to be the candidate mechanism for modulating network stability in this way, can efficiently perform pattern separation on a population simply via distinct phases for excitation and inhibition. While our model does not focus on the role of theta phase in recurrent medial–temporal lobe pathways, or on some key hippocampal functionality such as pattern separation and error-driven learning, we are able to show how preferential stimulus encoding can be achieved through the extrapolation of theta-induced effects at the neuronal level alone (i.e., when overlapping sensory input is in phase with theta-modulated LTP, then weights are stronger than when input is out of phase with these LTP fluctuations). We do this by marrying the notion of reward and punishment from STDP dynamics with the theta dynamics from the models of Hasselmo et al. (2002) and Norman et al. (2006), where distinct phases of theta provide contrasting functional windows that are vital for continued network stability when creating new memories. By exploring the emergent network properties that arise from these changes at the cellular level, we hope to show the multifaceted role that the theta frequency might play in memory formation, from both network and cellular perspectives.

This model is a continuation of the previous Sync/deSync model (Parish et al., 2018). Instead of receiving inputs from the NC populations that represent preferred and nonpreferred concepts, the two hippocampal subgroups receive inputs from the corresponding NC subgroups that represent visual and auditory stimuli. It has been suggested that most hippocampal neurons are modality invariant (Suthana and Fried, 2012). However, to make the model less complex yet remain possible to capture the general learning dynamic, each hippocampal subgroup receives a unimodal input. The visual and auditory stimuli used in the studies by Clouter et al. (2017) and Wang et al. (2018) were video and sound clips that contain complex contents (i.e., documentary images and music) and were not semantically related. It is highly likely that the neurons in each hippocampal subgroup code the representation rather than the modality information of each unimodal stimulus. Our model suggests that after learning, the two representations are successfully integrated, which the hippocampus needs to bind the semantically unrelated information into a coherent memory representation (Staresina and Davachi, 2009).

In the Sync/deSync model, though LTP and LTD are modulated by opposing phases of the ongoing theta rhythm, LTD happening at the theta peak is applied by a global passive decay. The passive decay is an exponential function that is multiplied by the complement of theta phases, which leads to maximal weight decay at theta peak and 0 at theta trough. The current model replaces this with an active theta phase-specific LTD, which is crucial to replicate the key findings from the study by Clouter et al. (2017), namely that the phase synchrony-induced memory advantage is specific to theta-modulated stimulus inputs. The Sync/deSync model from Parish et al. (2018) could replicate the behavior from the empirical data when stimuli were modulated at 4 Hz but failed to replicate another key finding of Clouter et al. (2017), namely that the memory enhancement was specific to the theta in-phase condition and was not present if stimuli were modulated at delta or alpha frequencies. When stimuli are modulated at nonhippocampally preferred frequencies (i.e., outside of theta), neuronal firing at the theta LTD phase is more likely to happen, and is less likely to happen at the LTP phase. The active theta phase-specific LTP and LTD thus allow more potentiation of synapses when stimulus inputs are theta modulated and in synchrony, and more LTD when stimulus inputs are modulated with delta and alpha frequencies, although they are in synchrony. This in turn allows the Sync/deSync model to be much more sensitive to very slight differences in the timings of concurrent stimuli, which was not an immediate concern during its original conception as a first proof-of-principle model that related oscillatory dynamics to human episodic memory formation.

In rodents, LTP and LTD can be induced by burst stimulation at opposing hippocampal theta phases, respectively (Pavlides et al., 1988; Huerta and Lisman, 1995; Hölscher et al., 1997; Hyman et al., 2003). In humans, theta-phase synchronization can enhance episodic memory performance (Clouter et al., 2017; Wang et al., 2018), as well as contingency knowledge between a conditioned stimulus (CS) and an aversive unconditioned stimulus, and affective rating on the CS in a fear-conditioning task (Plog et al., 2022). Our model can account for data from both rodents and humans, which connects human episodic memory behavior to synaptic modifications induced by non-neurophysiological stimulation in the animal brain. Our model provides an explanation for the findings in human electrophysiological studies, which show that hippocampal theta synchronization supports episodic encoding, especially when there is need to form arbitrary associations (Staudigl and Hanslmayr, 2013; Backus et al., 2016; Kota et al., 2020). Though those findings are correlational rather than causal, our model suggests that theta synchronization links to stronger synaptic weights between corresponding hippocampal neurons after encoding. Our model predicts that to induce substantial LTP in synapses between hippocampal neurons, cortical inputs must be in phase with the fluctuation of LTP. In contrast, if any input is desynchronized with the oscillatory changes of LTP, synaptic weights are very weak between the hippocampal neurons corresponding to those inputs. The phase of the hippocampal theta oscillation is reset to coordinate the timing of inputs with time windows of LTP, which is a key characteristic in our model to optimize learning. Indeed, hippocampal theta-phase resetting has been found to occur after stimulus onset during memory encoding (Rutishauser et al., 2010). Further, Kota et al. (2020) show in an intracranial EEG (iEEG) study that the ongoing hippocampal theta oscillation phase was reset to support episodic encoding. Such a phase reset indicates a mechanism for inputs that is more likely to undergo LTP (Rizzuto et al., 2003; McCartney et al., 2004; Mormann et al., 2005). Moreover, a rodent study recently revealed that using visual rhythmic stimulation at gamma frequency (40 Hz) can entrain gamma activity in the hippocampus and preserve hippocampal neurons and synapses (Adaikkan et al., 2019). Our model suggests that this could also happen in the human hippocampus, where theta phase is reset by sensory stimuli, thus aligning stimulus inputs to be more likely to induce LTP and form associations.

Investigating the cellular mechanisms of human learning and memory is challenging as it is relatively difficult to access human single neurons *in vivo*. Although there is evidence on STDP in the human hippocampus *in vitro* showing that LTP was induced in a wider time window compared with rodent hippocampal STDP (Silva et al., 2010). It is still unclear whether the same STDP rule applies in the human hippocampus *in vivo*. In our model, the classical STDP rule observed in the rodent hippocampus is implemented (Song et al., 2000; Bi and Poo, 2001; Parish et al., 2018). Our model efficiently forms new associations in a one-shot manner as required by episodic memory. In human single-neuron studies, neuron firing selectivity in the medial temporal lobe was extended to the learned, associated contextual pictures or temporal sequences (Ison et al., 2015; Reddy et al., 2015). Synaptic plasticity is very likely to be the circuit mechanism supporting episodic formation, which provides evidence that neuronal mechanisms of memory formation might be universal across species. However, our model predicts that STDP alone will result in a similar performance between 0° phase offset and 90° phase offset conditions, since LTP and LTD can happen at the same time if spikes are completely overlapped. Therefore, the two learning mechanisms, theta phase-dependent plasticity and STDP, are both essential to reproduce the empirical findings. They might interact to support episodic binding. STDP is a passive learning process so that weight changes happen at any time. However, if combined with theta phase-dependent learning, an active learning mechanism, the model accurately reproduces the empirical data. This is consistent with the notion that theta oscillations might compress the neural events that have happened on longer timescales, thus enabling STDP in action (Hanslmayr et al., 2016). In the rodent hippocampus, the LTP component of STDP is dependent on spike timing triggered during a theta oscillation, but not during a low-frequency activity (Wittenberg and Wang, 2006). This is consistent with our model, which shows that the learning effect is specific to theta-modulated inputs and all other conditions modulated by non-theta frequencies showed similarly low performance. Interestingly, a recent iEEG study showed that in the human hippocampus, cofiring of neurons at 20–40 ms indicated successful episodic memory formation, while cofiring at a longer delay of 60 ms resulted in forgetting. Moreover, such a subsequent memory-related cofiring effect was specific to the neuron pairs that were coupled to distal theta and local gamma oscillations. Reversal of the order of the neuron pairs resulted in an LTD-like effect, where a shorter delay resulted in subsequent forgetting (Roux et al., 2022). In the human hippocampus, the interaction between the two learning mechanisms, STDP and theta phase-dependent plasticity, likely provides an explanation for the role of the hippocampus in actively coordinating the timing of input arrival, hence binding the inputs into long-term memory.

Our model allows us to explore the interaction between STDP and theta phase-dependent learning beyond reproducing the empirical data by modulating inputs with more frequencies and phase offset conditions. As the modulation frequency becomes faster, learning from auditory to visual neurons is enhanced when the auditory stimulus leads with a short delay over the visual stimulus, while the pattern is reversed when the visual stimulus leads. This is consistent with the STDP model. However, learning in the higher frequency-modulated conditions is restricted by the theta phase-dependent learning rule, since firing does not always happen at the theta inhibitory phase. Therefore, the learning benefit in the higher frequencies is reduced compared with the learning benefit in the theta-synchronous condition. Future experiments where multisensory stimuli are modulated at higher frequencies and memory is cued with different sensory modalities can test the prediction if the resultant memory performance is more like STDP, as observed in our simulations. Another prediction that arises from our model is that the theta-phase synchronization-induced memory effect should be bidirectional. That is, synaptic connectivity from visual to auditory neurons (and auditory to visual neurons) is boosted by firing synchronously at the inhibitory theta phase. Learning in the three out-of-phase conditions is very low because of the nonoptimal timing of firing relative to the ongoing theta phase, and relative to the spike timing of receiving neurons.

We simulated our data by matching the stimulus input frequency with the hippocampal theta frequency. Our empirical data showed that there was variability in sensory entrainment. We therefore modeled the variability of input frequencies as well as hippocampal theta dynamics. Unsurprisingly, such variability weakened synaptic weight changes. This variability might come from attentional modulation (Tiitinen et al., 1993; Müller et al., 2006) or neocortex–hippocampus feedback loops where hippocampal theta rhythms entrain the neocortex to allow more optimal information transmission from the neocortex to the hippocampus (Sirota et al., 2008). This could be tested by directionality analyses in further experimental work using the same paradigm and recording hippocampal and cortical activity, or by implementing additional modules in future models. Our model reveals fluctuations of hippocampal weight change when inputs match with the hippocampal dynamics in different degrees of synchronization. Both inputs and the hippocampal theta are modeled with continuous 4 Hz cosine waves. However, empirical studies suggest that, compared with the clear theta peak in rodent studies, human hippocampal theta shows a less clear peak frequency with smaller peak height, and less continuity (Watrous et al., 2013; Qasim et al., 2021). Our model uses a small population of neurons that could limit the match between the hippocampal and input frequencies. A larger neuron population might be able to capture the interaction between continuous inputs and discontinuity of hippocampal theta. As discussed above, hippocampal theta might be entrained by external sensory stimuli to coordinate spike timing. Future modeling and empirical work should investigate to what degree hippocampal theta and the frequency of rhythmic sensory inputs benefit from matching to optimize learning.

In conclusion, our model successfully reproduced results from human episodic memory studies that show a causal role of theta-phase synchronization in episodic memory formation. Our findings provide new and important computational evidence for a combined role of two well known synaptic plasticity mechanisms to modulate theta phase-dependent memory effects in humans (i.e., STDP and theta phase-dependent plasticity).

## Synthesis

Reviewing Editor: Niko Busch, Westfalische Wilhelms-Universitat Munster Decisions are customarily a result of the Reviewing Editor and the peer reviewers coming together and discussing their recommendations until a consensus is reached. When revisions are invited, a fact-based synthesis statement explaining their decision and outlining what is needed to prepare a revision will be listed below. The following reviewer(s) agreed to reveal their identity: Jonathan Daume.

# Synthesis

Both reviews highlight the merit of your study, but they also agree on the need to improve the manuscript further. Specifically, I encourage you to follow #1’s suggestion that the model might be more powerful if it were extended such that its output is an observable, behavioral variable that could be compared to empirical data, instead of just synaptic weight change. Please also attend to the reviewers’ suggestions for clarifications and elaborations.

Finally, I appreciate that you are making the code available for reviewers and readers! I encourage you to add some kind of README file to briefly explain how to use or navigate these files.

# Reviewer 1

The authors describe a model of theta-phase dependent STDP learning and compare the simulated results of this model to prior empirical work demonstrating that synchronizing inputs at theta frequency improves memory. The authors make a reasonable argument that theta-phase or STDP alone are not sufficient to explain associative memory processes in the hippocampus. The paper is generally well-written and does a good job summarizing the model and justifying the specific choices in the model’s design.

My main concern with these results is the choice to present the model output (change in synaptic weights from the auditory to visual subgroups of model neurons) as a direct simulation of memory performance. This is a confusing choice, as it makes it more difficult to perform formal model comparison (i.e. how to compute the likelihood of the model without a comparison between real accuracy and simulated accuracy?), and also makes the interpretation of 3, 5, and 7 quite difficult. Ideally, the model could reproduce behavior in a way that matches the empirical data, and then the authors could use the model to make inferences about these hippocampal synaptic weights - a latent variable in the empirical data. I suggest the authors make a simple but critical change: implement a decision rule into the model which transforms the hippocampal weights into a mnemonic decision. Then, Figures 3, 5, 7 can feature direct comparisons of real behavior to simulated behavior, and the several instances of model selection can be performed in a more appropriate fashion (i.e. computing BIC scores). Otherwise, it becomes very difficult to assess the manuscript and its stated goal of providing mechanistic insight into prior empirical findings.

I have a few other minor questions and concerns:

1. Figure 3: The simulated neurons fire in an implausible range (50-150 Hz). Is this magnitude essential to producing the model results?

2. Figure 4: After about 1.5 seconds, the model produces perfectly symmetric bi-directional weight changes in the auditory and visual subgroups - is this finding supported by any empirical studies?

3. The model makes insufficient allowance for the empirical volatility of human theta. While small amounts of noise are allowed for frequency, these do not match the range of frequencies typically seen in human hippocampal theta (Qasim et al. 2021), and the authors do not account for the relative sparsity of human hippocampal theta (Watrous et al., 2013). The authors should either implement these features into the model or discuss these limitations in the Discussion.

4. The authors are very thorough in describing the task and model, but many of these details come group’s prior studies. The authors should more clearly re-iterate the change to their model relative to Parish 2018 throughout the manuscript, and more directly connect it to the novel findings in this paper.

5. It was not totally clear to me how the neocortical neurons in the model factored into the model outputs. This is important to the interpretation of this work, given the authors’ own prior findings of entrainment in this task in scalp EEG data (Wang et al. 2018).

# Reviewer 2

The authors present a computational study in which they develop a novel model to investigate the influence of theta phase as well as STDP on episodic memory. Using in-phase and out-phase visual and auditory inputs, they show that theta-phase-dependent STDP leads to a model output that is comparable to human behavior. The model is further compared to models lacking the theta phase or STDP component which reveals that especially the interplay between both components is crucial to replicate human behavior.

The manuscript is very well written, the methods are sound, and the results are convincing, interesting and relevant to the field. In general, I very much enjoyed reading it. I only have a few clarification questions that I will outline below in no particular order.

1) The model uses two separate subpopulations receiving auditory and visual sensory inputs. While that might make sense in the neocortex, it is less plausible in the hippocampus where most neurons are known to react to multimodal inputs. This is different in the Parish et al. 2018 model where two subpopulations code for preferred and nonpreferred (possibly multimodal) inputs which has been shown to be the case for neurons in the hippocampus. But it’s not clear to me how this makes sense for auditory and visual subpopulations in the hippocampus. To what degree is the model restricted to these separated, unimodal subpopulations? Could the model also be implemented using multimodal hippocampal populations that code for preferred stimuli rather than the sensory modality? Since this is an important limitation, it should at least be discussed.

2) If I understand the model correctly, synaptic efficacy should only change if LTP/LTD reaches a certain threshold. However, panels Aii/iii and Bii/iii in Figure 2 show changes even for values below this threshold. How can this be explained? Are the thresholds in panels ii referring to changes at a single synapse while the efficacy changes in panels iii are shown across all synapses? A better description of these results would be helpful.

3) The authors state multiple times (e.g., p. 35, line 742ff) that the active theta-phase-specific LTD, which is the main difference of their model to that of Parish et al., is crucial to replicate the nuances of human memory behavior from earlier studies. However, they don’t provide evidence for that claim. While they implement nice control models that show that removing the theta-phase or the STDP components from the model does not yield the same/worse results as the full model, they don’t show that the Parish et al model, which also has both components but in a less active manner, wouldn’t yield the same results. To make this statement and to show that their model is in fact superior to earlier versions of this model, I would like to ask the authors to also compare their new model to the Parish et al model. Does the new model still fit the behavioral results better when compared to the model from Parish et al., 2018?

4) The authors fit different models to the behavioral data of two earlier studies and find that the full model fits human behavioral data better than the two control models (Figure 7). I wonder why the authors here use data from the full model that did not contain noise. They showed earlier (Figure 2) that adding noise yields better comparable results to human behavior than the model without noise. So why not also using these simulations containing noise for the statistical comparisons in Figure 7? Is the full model still fitting human behavioral data better when noise is added either to the input or to the hippocampal frequency?

Minor:

- Since the behavioral results of Clouter et al, 2017 and Wang et al, 2018 are central to this manuscript, it would be helpful to describe the experimental paradigms of these two studies in more detail. It’s not clear what exactly has been done in these two studies from the current manuscript alone.

- p. 12, lines 226ff: It does not become immediately clear what the main difference is between the current model and the one published by Parish et al., 2018. The discussion makes this distinction clearer. It would be nice if the difference and the novelty of the current model could be made clear already at this point.

- P. 14, line 286: I assume the spike-trains of 4000 spikes/s represent background noise that each neuron is receiving. However, this is not clear from the text. I first assumed that random 4000 Hz spike trains were added to the spiking activity of each neuron which would of course be way too high. Please make this point clearer. Same for the hippocampal neurons in the next section.

- I suggest using the terms in-phase and out-of-phase instead of synchronous and asynchronous inputs. The “asynchronous” inputs are in fact also perfectly synchronous but just shifted in their phase offset. Out-of-phase inputs might therefore be the more precise term. That being said, it would be interesting to see what the model output was for an actual asynchronous input (e.g., by adding noise to the phase of both inputs). Depending on the degree of noise added to the phase of the inputs it could lead to results comparable to the out-of-phase conditions.

- It would be helpful to have a readme or demo file for the software package that explains how to run the code. It was not clear to me how to navigate through the different functions.

## Author Response

We would like to thank the reviewers for their insightful comments and the editor for overseeing the review process and provide us with helpful comments on how to address the reviewers’ points.

Please find below our detailed responses. We hope to have adequately addressed all reviewers’ concerns.

# Reviewer 1

The authors describe a model of theta-phase dependent STDP learning and compare the simulated results of this model to prior empirical work demonstrating that synchronizing inputs at theta frequency improves memory. The authors make a reasonable argument that theta-phase or STDP alone are not sufficient to explain associative memory processes in the hippocampus. The paper is generally well-written and does a good job summarizing the model and justifying the specific choices in the model’s design.

My main concern with these results is the choice to present the model output (change in synaptic weights from the auditory to visual subgroups of model neurons) as a direct simulation of memory performance. This is a confusing choice, as it makes it more difficult to perform formal model comparison (i.e. how to compute the likelihood of the model without a comparison between real accuracy and simulated accuracy?), and also makes the interpretation of 3, 5, and 7 quite difficult.

Ideally, the model could reproduce behavior in a way that matches the empirical data, and then the authors could use the model to make inferences about these hippocampal synaptic weights - a latent variable in the empirical data. I suggest the authors make a simple but critical change: implement a decision rule into the model which transforms the hippocampal weights into a mnemonic decision. Then, Figures 3, 5, 7 can feature direct comparisons of real behavior to simulated behavior, and the several instances of model selection can be performed in a more appropriate fashion (i.e. computing BIC scores). Otherwise, it becomes very difficult to assess the manuscript and its stated goal of providing mechanistic insight into prior empirical findings.

Response: We appreciate the reviewer’s comments on converting the simulated hippocampal weights into recall behaviour using a decision rule. We have added this analysis using a threshold that was determined by the first 10% of weights in each trial across all conditions, see p. 19 lines 393 - 403: ‘To directly compare the simulated memory performance with the empirical findings, after learning, hippocampal weights were averaged between 2.75 and 3 s (Figure 3Ai) for each trial in the theta, delta, alpha and no-flicker conditions. A memory decision index was computed by comparing the mean hippocampal weight value with a threshold. The threshold was set to the 10th percentile of the values in each trial across the above-mentioned conditions. The memory performance of a trial (recalled successfully or not) was determined by whether the value was above the threshold. The threshold was also applied to other simulations when noise was introduced to the input frequency or hippocampal theta frequency, as well as the theta-phase-only and STDP-only models. The model comparisons were statistically tested by the Bayesian Information Criterion (BIC).”

The resulting patterns did not differ from the previous results where we used the mean of the weights, see Figure 3-1: Figure 3-1. Simulated memory decision index. A, Simulated memory decision index and empirical data from Clouter et al. (2017) and Wang et al. (2018). Accuracy is normalized by subtracting the mean over all 4 phase offset conditions to make the between studies data more comparable. Ai, simulations of pure input frequency, 4 Hz, hippocampal frequency, 4 Hz, and EC phase offset, 180{degree sign} from hippocampal theta. Aii, simulations of input frequencies randomly drawn from normal distribution with mean 4, and standard deviation (STD) 0.015*4, pure hippocampal frequency, 4 Hz, and EC phase offset, 180{degree sign} from hippocampal theta. Aiii, simulations of pure input frequency, 4 Hz, hippocampal frequencies randomly drawn from normal distribution with mean 4, and STD 0.02, and EC phase offsets randomly draw from normal distribution with mean 180{degree sign}, and STD 0.167. Error bars represent SE. Bi, simulations of pure input frequencies, hippocampal frequency, 4 Hz, and EC phase offset, 180{degree sign} from hippocampal theta. Bii & Biii, simulations of pure input frequency, 4 Hz, hippocampal frequencies randomly drawn from normal distribution with mean 4, and STD 0.02, and EC phase offsets randomly drawn from normal distribution with mean 180{degree sign}, and STD 0.167. All error bars represent SE. C & D, Model comparisons in simulated memory decision index between the full model and two alternative versions. C, simulated memory decision index of two versions of model was fit to the data from Clouter et al. (2017). Ci, The full model was compared with a theta-phase-learning only version of the model. Cii, Same as Ci, but the comparison was between the full model and an STDP only version of the model. D, Same as C, but simulated memory decision index of two versions of model was fit to the data from Wang et al. (2018). All simulations were done with pure input frequency, 4 Hz, hippocampal frequency, 4 Hz, and EC phase offset, 180{degree sign} from hippocampal theta. All error bars represent SE. The model comparisons were statistically tested by the Bayesian

Information Criterion (BIC). The BIC scores difference between the full model and theta-phase-only model is 0.72 for fitting to the Clouter et al. (2017) data and 0.54 for fitting to the Wang et al. (2018) data. The BIC scores difference between the full model and STDP-only model is 0.03 for fitting to the Clouter et al. (2017) data and -0.38 for fitting to the Wang et al. (2018) data. The BIC scores do not suggest that full model fits better to the data. This could be due to that BIC penalised more for the model with more parameters. The pattern of the full model of simulated memory decision index resembles the empirical data.

However, the non-linear all-or-none manner of the thresholding procedure loses the nuanced changes in the out-of-phase conditions. We have tried adjusting the thresholds with different decision rules. The current threshold seems to be the most ideal one. A more ideal one could be selected by more complex decision rules, which are likely at play during memory recall. However, modelling memory recall processes would be another modelling project in its own right and therefore is beyond the scope of our paper. Therefore, we decided to keep the results of weights but to include the results of simulated performance from the thresholding procedure (see Figure 3-1).

I have a few other minor questions and concerns:

1. Figure 3: The simulated neurons fire in an implausible range (50-150 Hz). Is this magnitude essential to producing the model results?

Response: We thank the reviewer for their careful observation. The high firing rate is because of the way we modelled learning to prevent learning from being triggered by singlet or doublet spike pairings. Instead, learning was triggered by a burst of spikes. This is consistent with experimental observations suggesting the importance of bursting for inducing plasticity (Huerta & Lisman, 1995), as shown in Figure 2.

2. Figure 4: After about 1.5 seconds, the model produces perfectly symmetric bi-directional weight changes in the auditory and visual subgroups - is this finding supported by any empirical studies?

Response: This is a good question. We do not know if there are any empirical studies supporting the symmetric bi-directional weight changes between the two subgroups. We used the model to make a prediction on the question of whether the theta-phase-synchronisation-induced memory effect still holds if memory is cued with a visual stimulus. Our model showed that there is no difference. The in-phase condition showed the largest weight changes from the visual subgroup to the auditory subgroup. Whether this is true remains to be tested experimentally. We would be appreciative and like to add the evidence into our Discussion if the reviewer knows any empirical studies that support non-symmetric bi-directional weight changes between the two subgroups.

3. The model makes insufficient allowance for the empirical volatility of human theta. While small amounts of noise are allowed for frequency, these do not match the range of frequencies typically seen in human hippocampal theta (Qasim et al. 2021), and the authors do not account for the relative sparsity of human hippocampal theta (Watrous et al., 2013). The authors should either implement these features into the model or discuss these limitations in the Discussion.

Response: We thank the reviewer for pointing out the important limitations. We followed the reviewer’s suggestion and added a paragraph in the Discussion section where we address these limitations, see p. 41, lines 894 - 905: ‘Our model reveals fluctuations of hippocampal weight change when inputs match with the hippocampal dynamics in different degrees of synchronisation. Both inputs and the hippocampal theta are modelled with continuous 4-Hz cosine waves. However, empirical studies suggest that, as compared to the clear theta peak in rodent studies, human hippocampal theta shows a less clear peak frequency with smaller peak height, and less continuity (Qasim et al., 2021; Watrous et al., 2013). Our model uses a small population of neurons that could limit the match between the hippocampal and input frequencies. A larger neuron population might be able to capture the interaction between continuous inputs and discontinuity of hippocampal theta. As discussed above, hippocampal theta might be entrained by external sensory stimuli to coordinate spike timing. Future modelling and empirical work should investigate to what degree hippocampal theta and the frequency of rhythmic sensory inputs benefit from matching to optimise learning.”

4. The authors are very thorough in describing the task and model, but many of these details come group’s prior studies. The authors should more clearly re-iterate the change to their model relative to Parish 2018 throughout the manuscript, and more directly connect it to the novel findings in this paper.

Response: The difference between the current model and Parish et al. (2018) has been stated more clearly on p. 12 lines 233 to 234: ‘The current model replaces the exponential passive decay with the active theta-phase-specific LTD, which was crucial for the model to replicate further experimental work (Clouter et al., 2017; Wang et al., 2018).”

5. It was not totally clear to me how the neocortical neurons in the model factored into the model outputs. This is important to the interpretation of this work, given the authors’ own prior findings of entrainment in this task in scalp EEG data (Wang et al. 2018).

Response: The neocortical neurons receive corresponding inputs that are pure 4 Hz sinewaves with different phase offsets. To reflect the findings from Wang et al., which shows that single trial fluctuation of the strength in sensory entrainment predicts later memory performance, we added noise to the input frequency (Figure 3Cii). We found that learning in the in-phase condition decreased, as did the enhancement of learning in the in-phase condition, compared to the out-of-phase conditions, which resembled the empirical findings. To implement the suggestion from reviewer 2, we also added noise to the phase offsets between inputs, which mimics more the findings from Wang et al. (2018). The results (Figure 5-1) were similar as shown in the conditions where input noise was added.

# Reviewer 2

The authors present a computational study in which they develop a novel model to investigate the influence of theta phase as well as STDP on episodic memory. Using in-phase and out-phase visual and auditory inputs, they show that theta-phase-dependent STDP leads to a model output that is comparable to human behavior. The model is further compared to models lacking the theta phase or

STDP component which reveals that especially the interplay between both components is crucial to replicate human behavior.

The manuscript is very well written, the methods are sound, and the results are convincing, interesting and relevant to the field. In general, I very much enjoyed reading it. I only have a few clarification questions that I will outline below in no particular order.

1) The model uses two separate subpopulations receiving auditory and visual sensory inputs. While that might make sense in the neocortex, it is less plausible in the hippocampus where most neurons are known to react to multimodal inputs. This is different in the Parish et al. 2018 model where two subpopulations code for preferred and nonpreferred (possibly multimodal) inputs which has been shown to be the case for neurons in the hippocampus. But it’s not clear to me how this makes sense for auditory and visual subpopulations in the hippocampus. To what degree is the model restricted to these separated, unimodal subpopulations? Could the model also be implemented using multimodal hippocampal populations that code for preferred stimuli rather than the sensory modality? Since this is an important limitation, it should at least be discussed.

Response: We thank the reviewer for pointing out this limitation. We have acknowledged that a limitation of the current model is its use of hippocampal subgroups to represent each unimodal stimulus separately in the Discussion, see p. 36, lines 768 - 781: ‘Instead of receiving inputs from the NC populations that represent preferred and non-preferred concepts, the two hippocampal subgroups receive inputs from the corresponding NC subgroups that represent visual and auditory stimuli. It has been suggested that most hippocampal neurons are modality-invariant (Suthana & Fried, 2012). However, to make the model less complex yet remain possible to capture the general learning dynamic, each hippocampal subgroup receives a unimodal input. The visual and auditory stimuli used in Clouter et al. (2017) and Wang et al. (2018) were video and sound clips that contain complex contents, i.e., documentary and music and were not semantically related. It is highly likely that the neurons in each hippocampal subgroup code the representation rather than modality information of each unimodal stimulus. Our model suggests that after learning, the two representations are successfully integrated, which the hippocampus needs to bind the semantically unrelated information into a coherent memory representation (Staresina & Davachi, 2009).”

2) If I understand the model correctly, synaptic efficacy should only change if LTP/LTD reaches a certain threshold. However, panels Aii/iii and Bii/iii in Figure 2 show changes even for values below this threshold. How can this be explained? Are the thresholds in panels ii referring to changes at a single synapse while the efficacy changes in panels iii are shown across all synapses? A better description of these results would be helpful.

Response: We thank the reviewer for their thorough observation. We would like to clarify that if the panels Aii/iii and Bii/iii in Figure 2 are zoomed in slightly, there is a very small fraction over the threshold. The value that potential plasticity increases is 0.65 whereas the threshold is 1. When taking into consideration the exponential decay of potential plasticity, the 2nd spike elicits a very small weight change. This is shown more clearly in Figure 2C for 2 spikes in a burst (which is in line with experimental observations). The % change value in both figures is the average across involved synapses, not single synapses. We have updated this in the caption: ‘Overall synaptic change was calculated as the percentage difference to a baseline period, taking an average across all involved synapses.”

3) The authors state multiple times (e.g., p. 35, line 742ff) that the active theta-phase-specific LTD, which is the main difference of their model to that of Parish et al., is crucial to replicate the nuances of human memory behavior from earlier studies. However, they don’t provide evidence for that claim. While they implement nice control models that show that removing the theta-phase or the STDP components from the model does not yield the same/worse results as the full model, they don’t show that the Parish et al model, which also has both components but in a less active manner, wouldn’t yield the same results. To make this statement and to show that their model is in fact superior to earlier versions of this model, I would like to ask the authors to also compare their new model to the Parish et al model. Does the new model still fit the behavioral results better when compared to the model from Parish et al., 2018?

Response: The reviewer’s comment on comparing the performance produced by the old model with the new model is valid. The model from Parish et al., (2018) did not implement the active theta-phase-specific LTD; instead, an exponential passive decay that was multiplied by (1-θ(t)) was implemented. The model could replicate the behaviour from the experiments where stimuli were modulated at 4 Hz (Clouter et al. 2017 & Wang et al. 2018). We did not show the results from the old model because it failed to replicate one of the key findings from Clouter et al. (2017). Clouter et al. (2017) showed that the memory enhancement was specific to theta synchronous condition and was not found if the stimuli were modulated at delta (1.7 Hz) or alpha (10.5 Hz) frequencies. Since the LTP and LTD in the Parish et al. (2018) model were both multiplied by the phase of theta, if a neuron fires at theta peak, in-phase stimuli at faster modulation frequency (i.e. 10.5 Hz) would also enhance learning as compared to out-of-phase stimuli. The new model that implements the active theta-phase-specific LTD overcomes this issue and successfully reproduces the frequency specificity shown by Clouter et al. (2018). We added these clarifications to the Discussion and highlighted in p. 37, lines 788 to 801: ‘The Sync/deSync model from Parish et al. (2018) could replicate the behaviour from the empirical data when stimuli were modulated at 4 Hz but failed to replicate another key finding of Clouter et al. (2018) namely that the memory enhancement was specific to the theta in-phase condition and was not present if stimuli were modulated at delta or alpha frequencies. When stimuli are modulated at non-hippocampally preferred frequencies (i.e. outside of theta), neuronal firing at the theta LTD phase is more likely to happen, and less likely to happen at the LTP phase. The active theta-phase-specific LTP and LTD thus allows more potentiation of synapses when stimulus inputs are theta modulated and in synchrony, and more LTD when stimulus inputs are modulated with delta and alpha frequencies, even though they are in synchrony. This in turn allows the Sync/deSync model to be much more sensitive to very slight differences in the timings of concurrent stimuli, which was not an immediate concern during its original conception as a first proof of principle model that related oscillatory dynamics to human episodic memory formation.”

4) The authors fit different models to the behavioral data of two earlier studies and find that the full model fits human behavioral data better than the two control models (Figure 7). I wonder why the authors here use data from the full model that did not contain noise. They showed earlier (Figure 2) that adding noise yields better comparable results to human behavior than the model without noise. So why not also using these simulations containing noise for the statistical comparisons in Figure 7? Is the full model still fitting human behavioral data better when noise is added either to the input or to the hippocampal frequency?

Response: The simulations with input noise and noise of hippocampal theta all reproduced similar behaviour as did the simulations with pure inputs and hippocampal theta, as shown in Figure 3C. Therefore, we present only the results with pure inputs and hippocampal theta.

Minor: - Since the behavioral results of Clouter et al, 2017 and Wang et al, 2018 are central to this manuscript, it would be helpful to describe the experimental paradigms of these two studies in more detail. It’s not clear what exactly has been done in these two studies from the current manuscript alone.

Response: A description of the paradigm was added to p. 16, lines 322 - 329: ‘The human episodic memory paradigm we chose to simulate is a multisensory associative memory paradigm from Clouter et al. (2017). Amplitude of neutral sound clips (3 s) and luminance of neutral movie clips (3 s) were modulated with a 4 Hz sine wave. The phase offsets between the modulation of movies and sounds were 0{degree sign} (in-phase) or 90{degree sign}, 180{degree sign} and 270{degree sign} (out-of-phase). Participants were asked to remember the association between a sound and a movie. After a distractor task, they were presented with one of the sounds and asked to choose, from four still images, which image was from the movie that they saw earlier paired with the sound.” - p. 12, lines 226ff: It does not become immediately clear what the main difference is between the current model and the one published by Parish et al., 2018. The discussion makes this distinction clearer. It would be nice if the difference and the novelty of the current model could be made clear already at this point.

Response: The difference between the current model and Parish et al. (2018) has been stated more clearly on p. 12 lines 233 to 234: ‘The current model replaces the exponential passive decay with the active theta-phase-specific LTD, which was crucial for the model to replicate further experimental work (Clouter et al., 2017; Wang et al., 2018).” - P. 14, line 286: I assume the spike-trains of 4000 spikes/s represent background noise that each neuron is receiving. However, this is not clear from the text. I first assumed that random 4000 Hz spike trains were added to the spiking activity of each neuron which would of course be way too high. Please make this point clearer. Same for the hippocampal neurons in the next section.

Response: The reviewer is correct. We have made this point clearer by changing the text to ‘Background noise that each neuron is receiving’ in p. 14, line 286 and p. 15, line 298: ‘Background noise for each NC neuron is receiving was estimated by Poisson distributed spike-trains (4000 spikes/s, Wmax = 0.023).” ‘Background noise for each hippocampal neuron is receiving was estimated by Poisson distributed spike-trains (1500 spikes/s, Wmax = 0.015).” - I suggest using the terms in-phase and out-of-phase instead of synchronous and asynchronous inputs. The “asynchronous” inputs are in fact also perfectly synchronous but just shifted in their phase offset. Out-of-phase inputs might therefore be the more precise term. That being said, it would be interesting to see what the model output was for an actual asynchronous input (e.g., by adding noise to the phase of both inputs). Depending on the degree of noise added to the phase of the inputs it could lead to results comparable to the out-of-phase conditions.

Response: We have changed the ‘synchronous’ and ‘asynchronous’ terminology to ‘in-phase’ and ‘out-of-phase’, respectively, where appropriate. We have added the analysis of adding noise to the phases of inputs, see p. 17 lines 357 - 360: ‘To model the trial-by-trial variability of phase offsets between inputs, noise was introduced to phases of two input stimuli by randomising the phases over normal distributions centred at the corresponding phases i.e., 0{degree sign}, 90{degree sign}, 180{degree sign} and 270{degree sign} with STD of 5.” The results did not differ from the results of adding noise to the hippocampal theta frequencies. We report these results in Supplementary Figure 5-1: Figure S4. Weight changes as a function of degree of phase synchronisation in theta modulated condition and other control conditions. Simulations of pure input frequency, 4 Hz, hippocampal frequency, 4 Hz, and EC phase offset, 180{degree sign} from hippocampal theta. Input phases randomly drawn from normal distributions with mean 0{degree sign}, 90{degree sign}, 180{degree sign} and 270{degree sign}, and STD 5. All error bars represent SE. Black dots represent hippocampal weights after learning. Gray dots represent hippocampal weights averaged between -1.75 s and 0 during pre-stimulus baseline. - It would be helpful to have a readme or demo file for the software package that explains how to run the code. It was not clear to me how to navigate through the different functions.

Response: A readme file has been added to each subfolder.
