# Supplementary figures and images for: Interaction between Theta Phase and Spike Timing-Dependent Plasticity Simulates Theta-Induced Memory Effects
Source: eNeuro. 2023 Mar 10;10(3):ENEURO.0333-22.2023. doi: 10.1523/ENEURO.0333-22.2023 (PMC10012328; doi:10.1523/ENEURO.0333-22.2023)

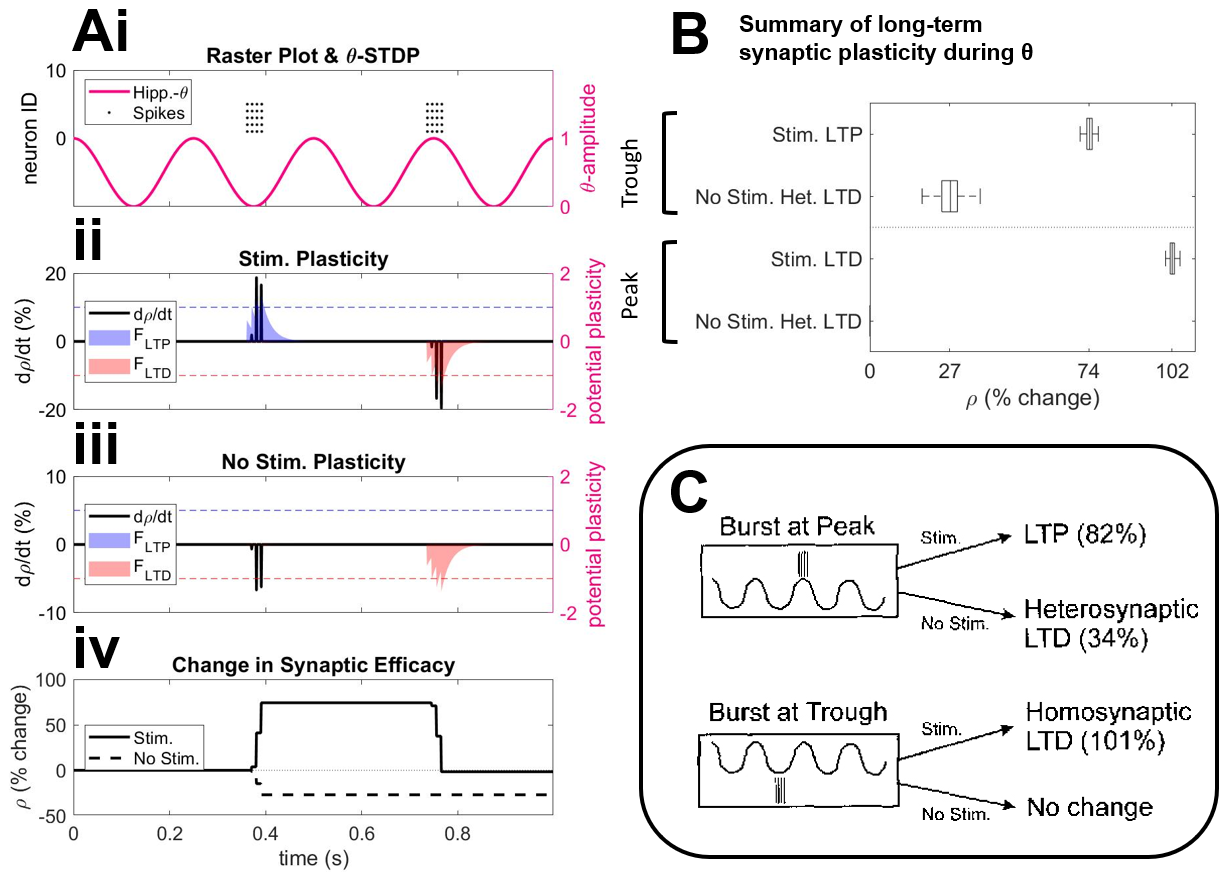

Supplement: Figure 2-1 — Summary of long-term synaptic plasticity during theta. Ai, A single simulation depicting synaptic plasticity after two bursts of 4 spikes at 100 Hz (amplitude, 3 pA), first at the trough and then at the peak of ongoing theta oscillations. Aii, Aiii, Potential plasticity induced by spike pairings is calculated via functions (shaded regions; see Eqs. 4.1, 4.2) for LTP (blue) and LTD (red). At the time of spike event, synapses undergo potentiation or depression (black lines; see Eqs. 5.1, 5.2) if potential plasticity is above or below a potentiation or depression threshold (blue and red dotted lines, respectively). Aiv, Overall synaptic change is calculated as a percentage of a baseline period through time. Aiii, For this simulation only, we here make use of an additional equation that induces heterosynaptic LTD on nonstimulated (No Stim.) pathways, that was proportional to the amount of LTP that occurred on stimulated pathways (Stim.; see Eqs. 5.1, 5.2). B, A summary of synaptic plasticity during theta, where confidence bars indicate variability over 100 trials. The percentage for LTP on stimulated pathways (Stim. LTP) and heterosynaptic LTD on unstimulated pathways (No Stim. Het. LTD) is the absolute of the percentage change of synapses relative to a baseline period. The percentage for LTD on stimulated pathways (Stim. LTD) is the depression produced by a single burst compared to the potentiation that preceded it (e.g., 100% is complete reversal). C, Experimental observations (Huerta and Lisman, 1995) surmising the relationship of theta and neuronal activity. Equation 5-1. Probability of synaptic plasticity on any j value of a spiking neuron where postsynaptic LTP has taken place. Equations 5-2.1, 5-2.2. Heterosynaptic LTD (LTDH) acting on any j that has met the probability for change in Extended Data Equation 2-1. The amount of heterosynaptic LTD is further normalized against the amount of prior LTP on the postsynaptic connection [dρ(1)/dt from Eq. 6.1], such that t [file enu-eN-NWR-0333-22-s05.tif]

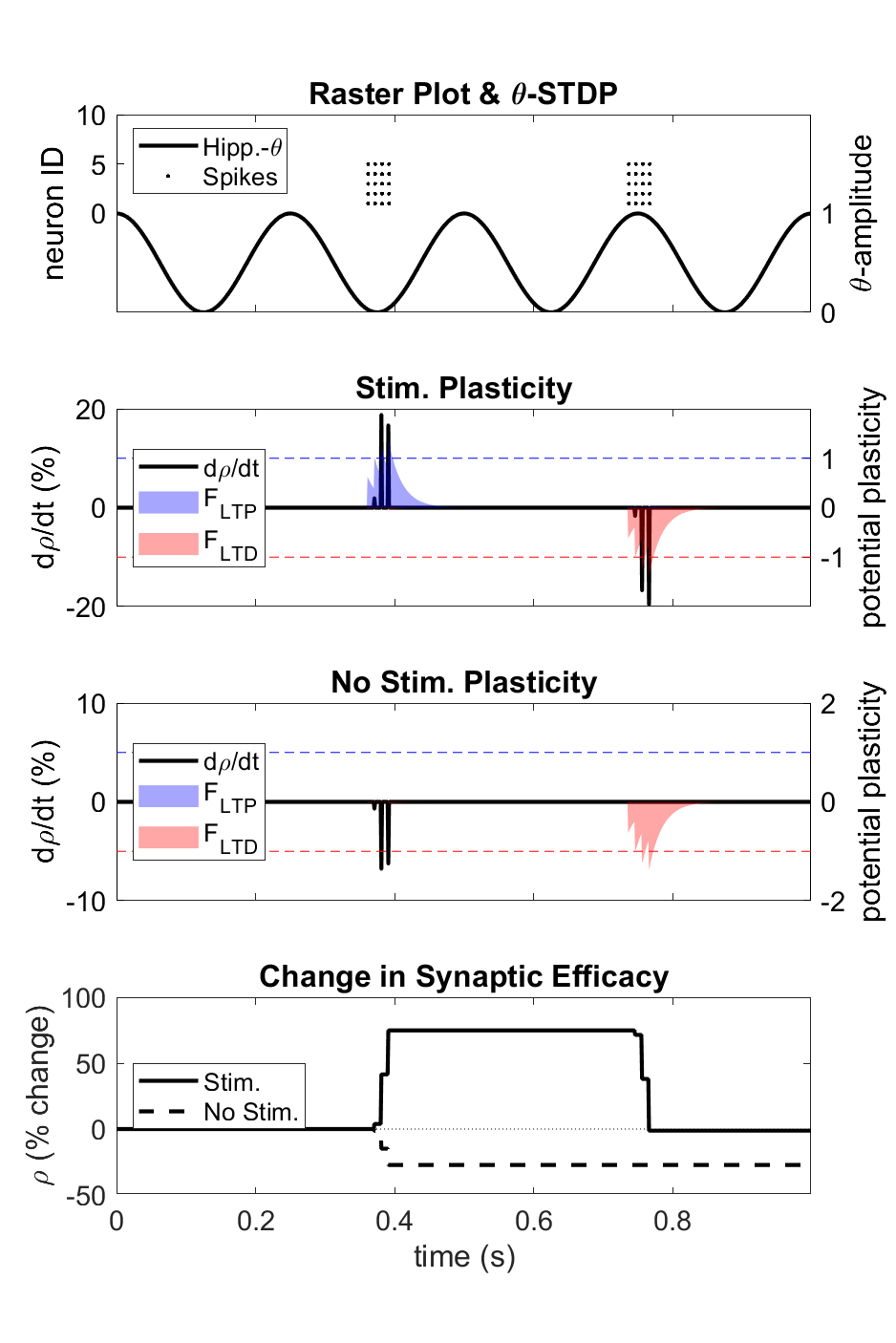

Supplement: Extended Data 1 — Human data and the MATLAB code of the model to run the simulations., Download [file enu-eN-NWR-0333-22-s07.zip › Sync-deSync-TIME-revision/HL_model/demo/het-STDP_multi-burst_100T/weight_change_both_4-shocks.tiff]

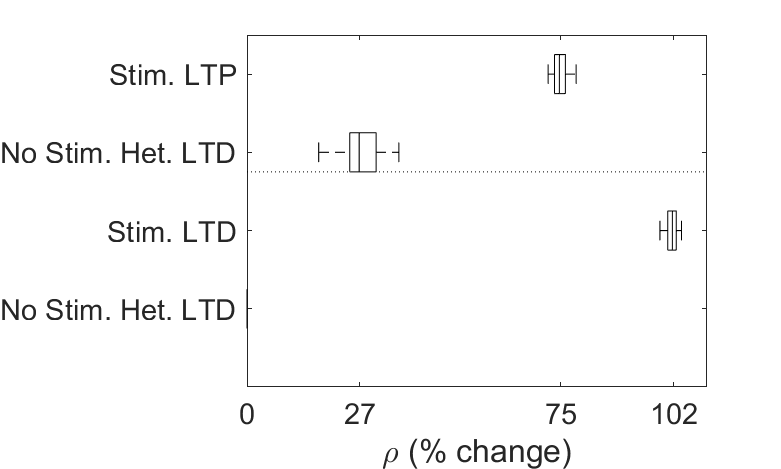

Supplement: Extended Data 1 — Human data and the MATLAB code of the model to run the simulations., Download [file enu-eN-NWR-0333-22-s07.zip › Sync-deSync-TIME-revision/HL_model/demo/het-STDP_multi-burst_100T/weight_change_theta_both_4-shocks.tiff]

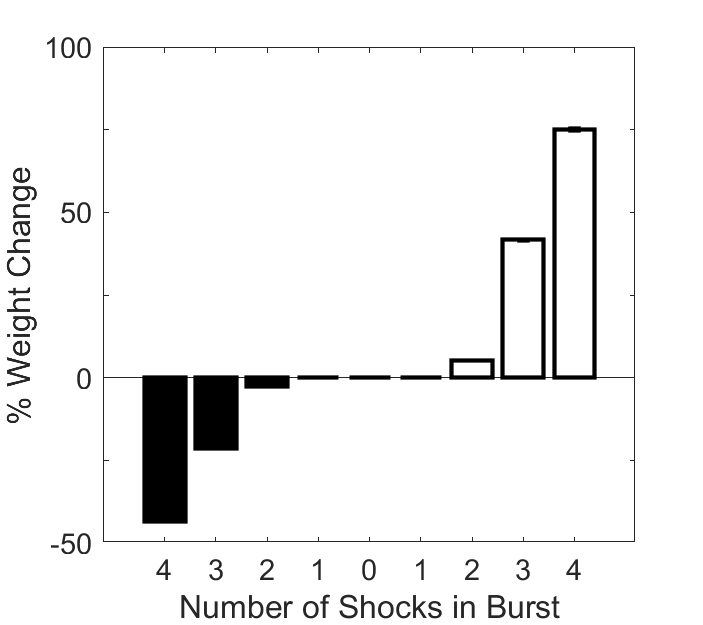

Supplement: Extended Data 1 — Human data and the MATLAB code of the model to run the simulations., Download [file enu-eN-NWR-0333-22-s07.zip › Sync-deSync-TIME-revision/HL_model/demo/het-STDP_single-burst_25T/weight_change.tiff]

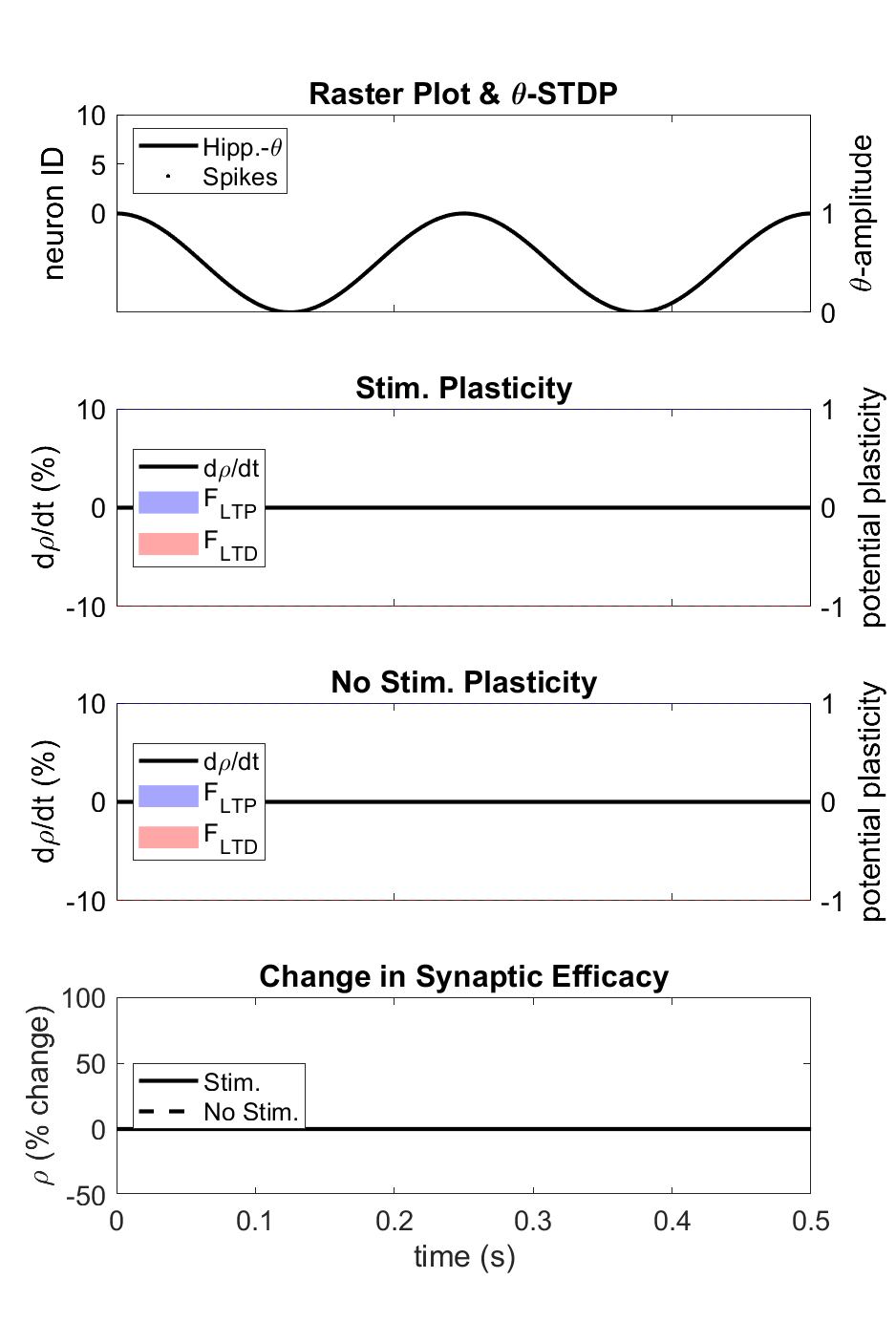

Supplement: Extended Data 1 — Human data and the MATLAB code of the model to run the simulations., Download [file enu-eN-NWR-0333-22-s07.zip › Sync-deSync-TIME-revision/HL_model/demo/het-STDP_single-burst_25T/weight_change_peak_0-shocks.tiff]

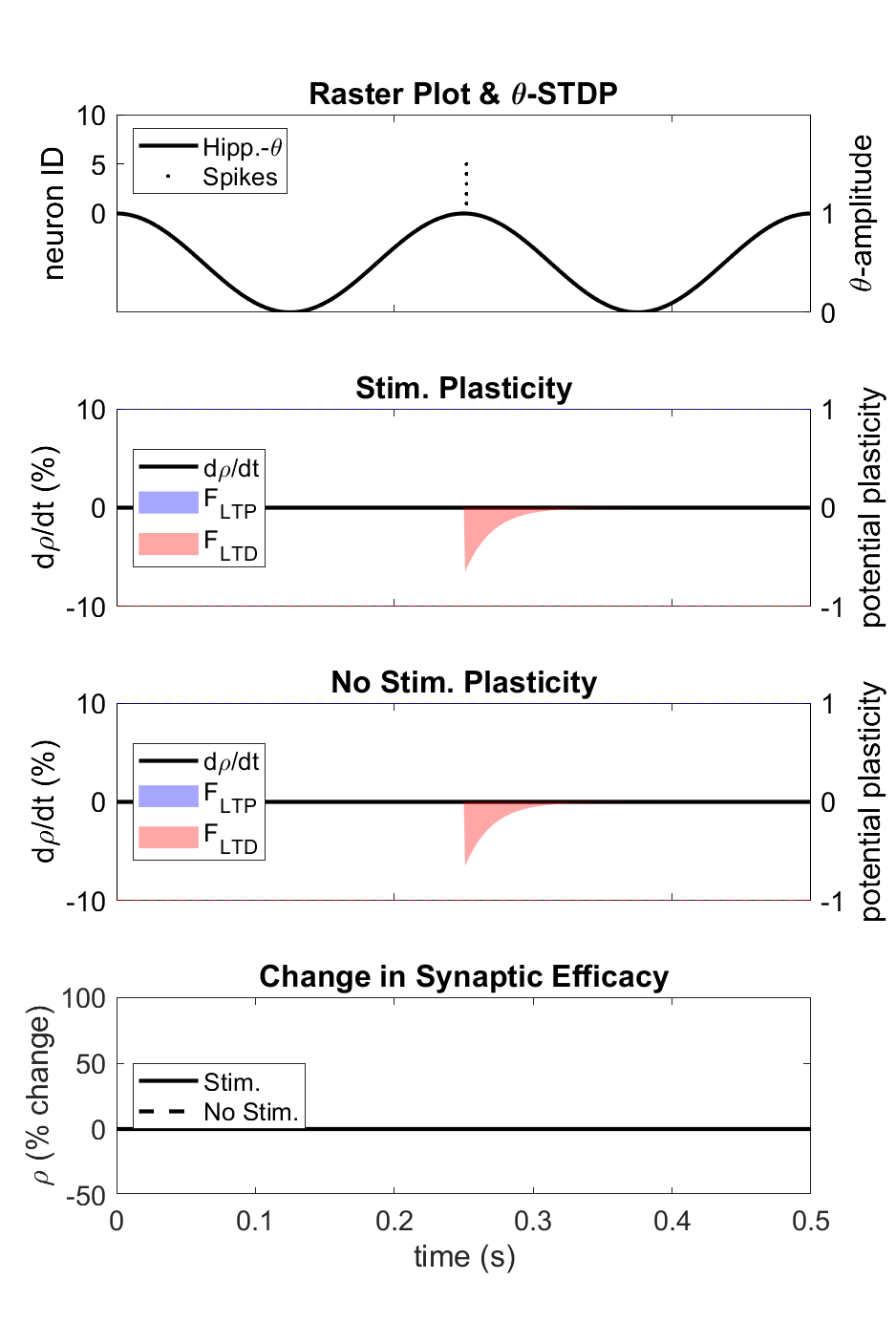

Supplement: Extended Data 1 — Human data and the MATLAB code of the model to run the simulations., Download [file enu-eN-NWR-0333-22-s07.zip › Sync-deSync-TIME-revision/HL_model/demo/het-STDP_single-burst_25T/weight_change_peak_1-shocks.tiff]

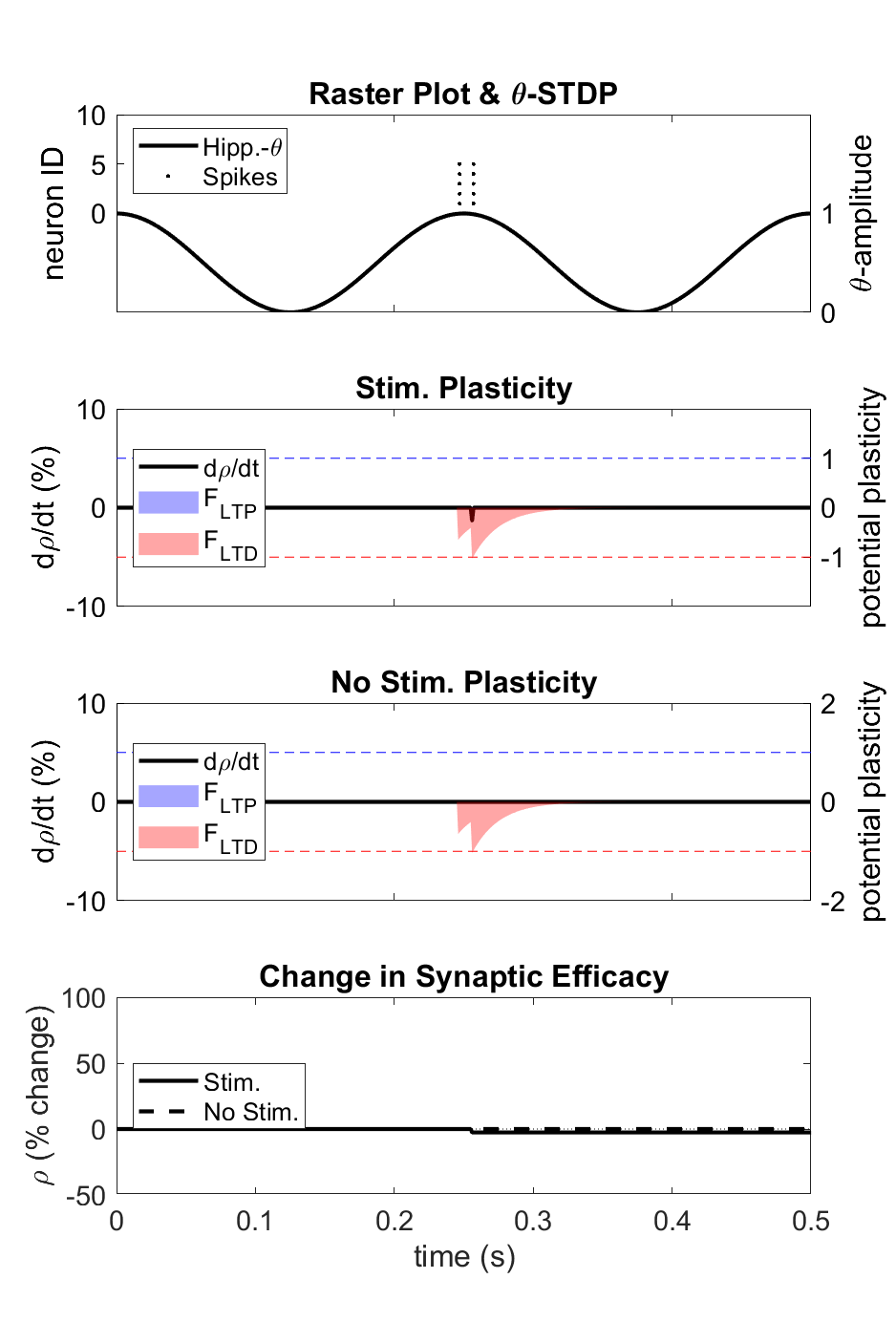

Supplement: Extended Data 1 — Human data and the MATLAB code of the model to run the simulations., Download [file enu-eN-NWR-0333-22-s07.zip › Sync-deSync-TIME-revision/HL_model/demo/het-STDP_single-burst_25T/weight_change_peak_2-shocks.tiff]

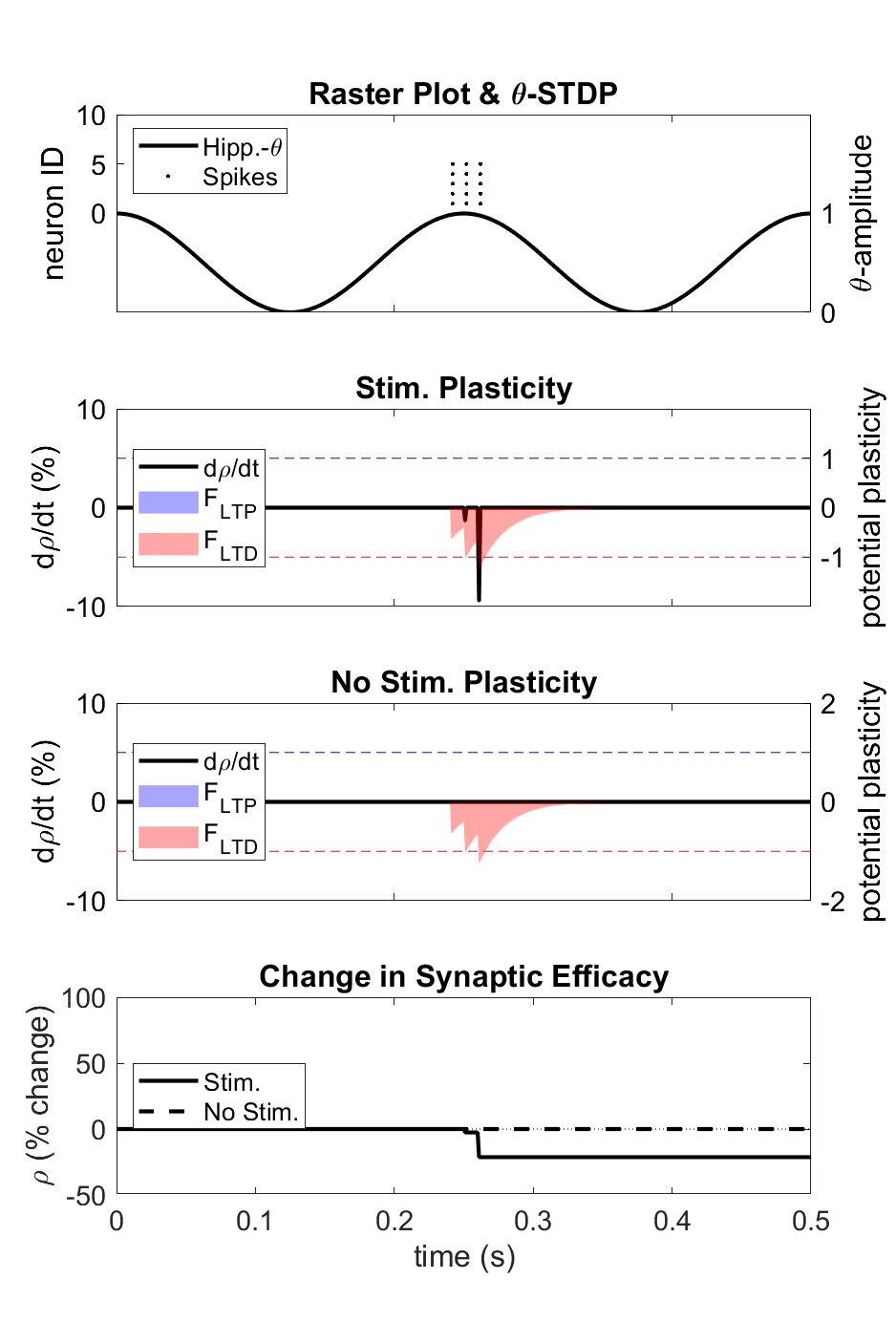

Supplement: Extended Data 1 — Human data and the MATLAB code of the model to run the simulations., Download [file enu-eN-NWR-0333-22-s07.zip › Sync-deSync-TIME-revision/HL_model/demo/het-STDP_single-burst_25T/weight_change_peak_3-shocks.tiff]

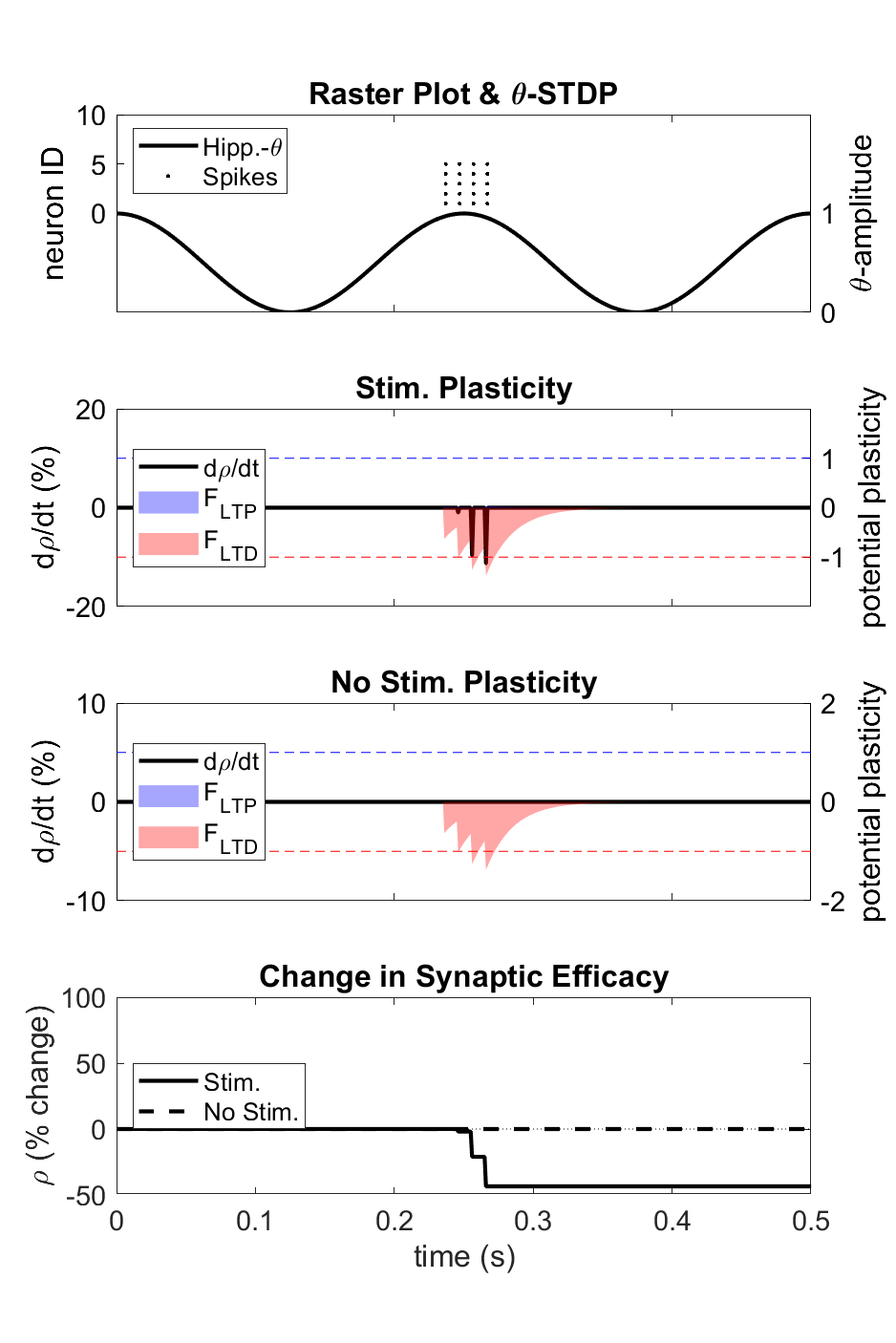

Supplement: Extended Data 1 — Human data and the MATLAB code of the model to run the simulations., Download [file enu-eN-NWR-0333-22-s07.zip › Sync-deSync-TIME-revision/HL_model/demo/het-STDP_single-burst_25T/weight_change_peak_4-shocks.tiff]

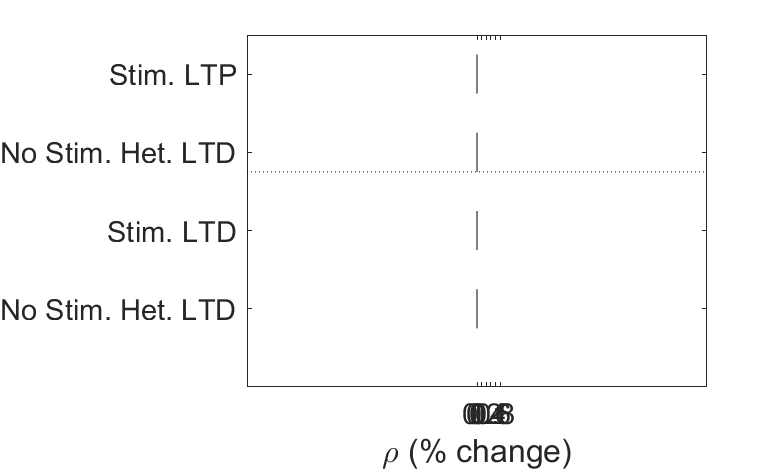

Supplement: Extended Data 1 — Human data and the MATLAB code of the model to run the simulations., Download [file enu-eN-NWR-0333-22-s07.zip › Sync-deSync-TIME-revision/HL_model/demo/het-STDP_single-burst_25T/weight_change_theta_peak_0-shocks.tiff]

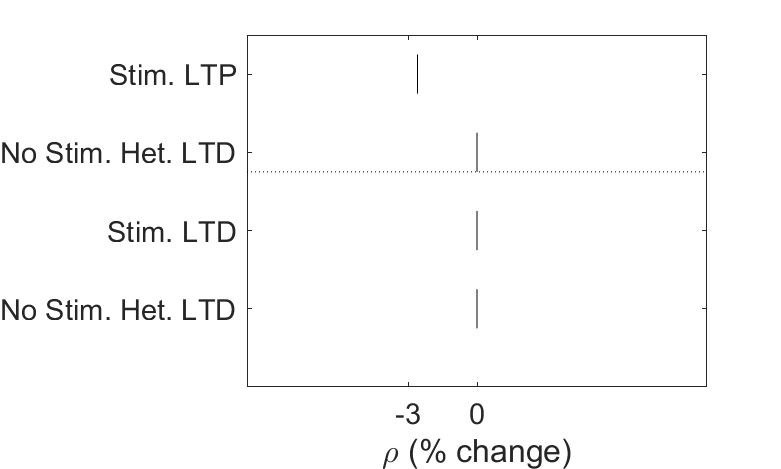

Supplement: Extended Data 1 — Human data and the MATLAB code of the model to run the simulations., Download [file enu-eN-NWR-0333-22-s07.zip › Sync-deSync-TIME-revision/HL_model/demo/het-STDP_single-burst_25T/weight_change_theta_peak_2-shocks.tiff]

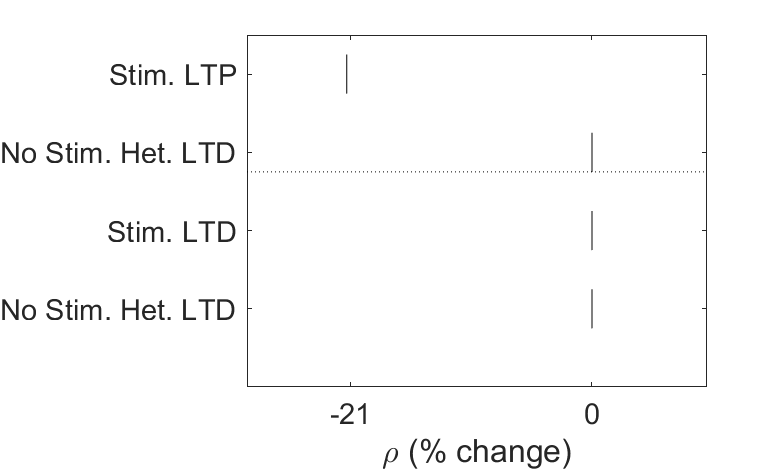

Supplement: Extended Data 1 — Human data and the MATLAB code of the model to run the simulations., Download [file enu-eN-NWR-0333-22-s07.zip › Sync-deSync-TIME-revision/HL_model/demo/het-STDP_single-burst_25T/weight_change_theta_peak_3-shocks.tiff]

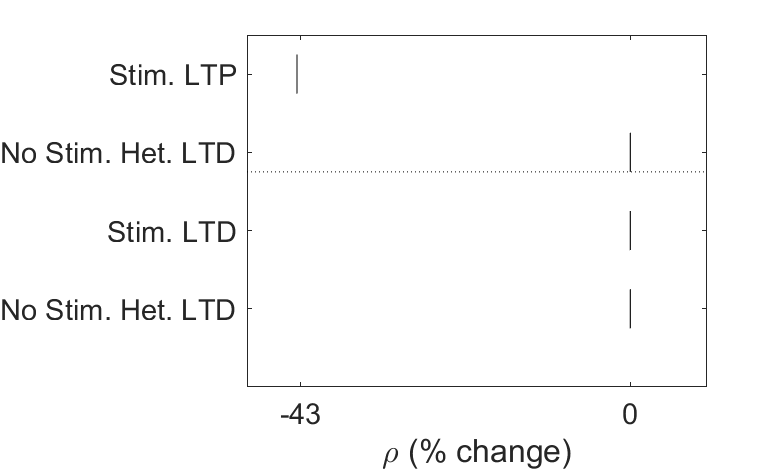

Supplement: Extended Data 1 — Human data and the MATLAB code of the model to run the simulations., Download [file enu-eN-NWR-0333-22-s07.zip › Sync-deSync-TIME-revision/HL_model/demo/het-STDP_single-burst_25T/weight_change_theta_peak_4-shocks.tiff]

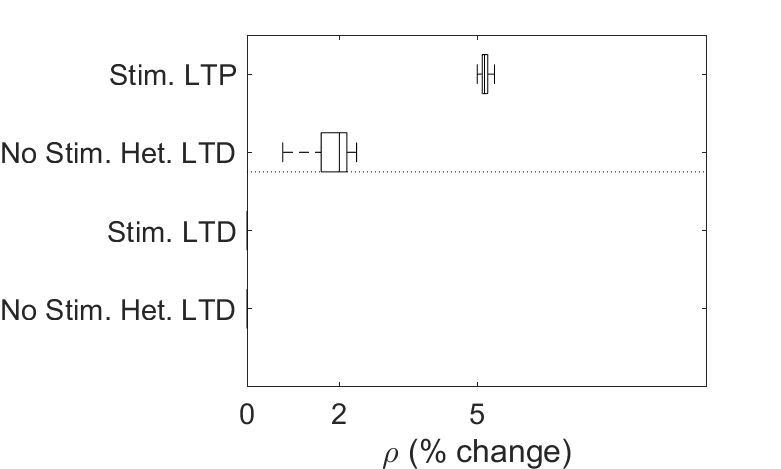

Supplement: Extended Data 1 — Human data and the MATLAB code of the model to run the simulations., Download [file enu-eN-NWR-0333-22-s07.zip › Sync-deSync-TIME-revision/HL_model/demo/het-STDP_single-burst_25T/weight_change_theta_trough_2-shocks.tiff]

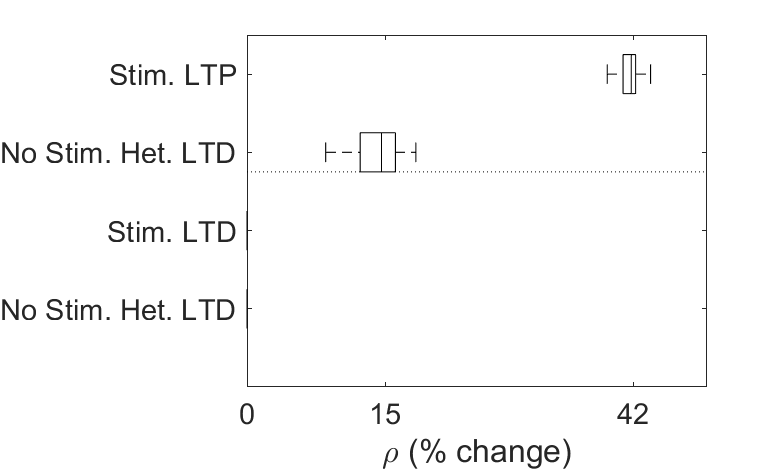

Supplement: Extended Data 1 — Human data and the MATLAB code of the model to run the simulations., Download [file enu-eN-NWR-0333-22-s07.zip › Sync-deSync-TIME-revision/HL_model/demo/het-STDP_single-burst_25T/weight_change_theta_trough_3-shocks.tiff]

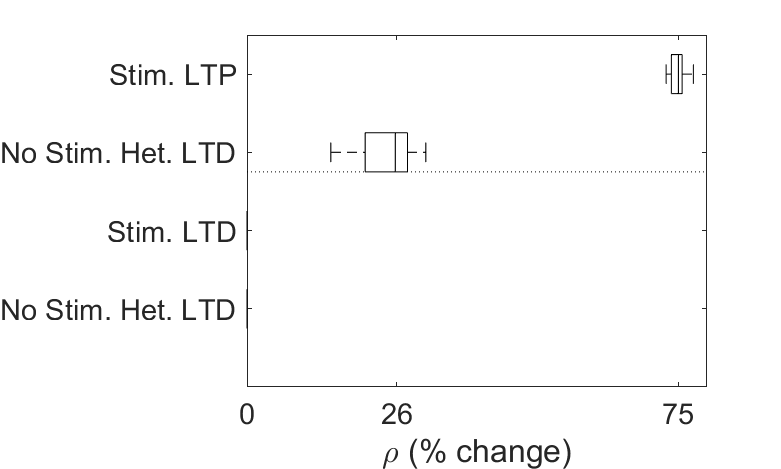

Supplement: Extended Data 1 — Human data and the MATLAB code of the model to run the simulations., Download [file enu-eN-NWR-0333-22-s07.zip › Sync-deSync-TIME-revision/HL_model/demo/het-STDP_single-burst_25T/weight_change_theta_trough_4-shocks.tiff]

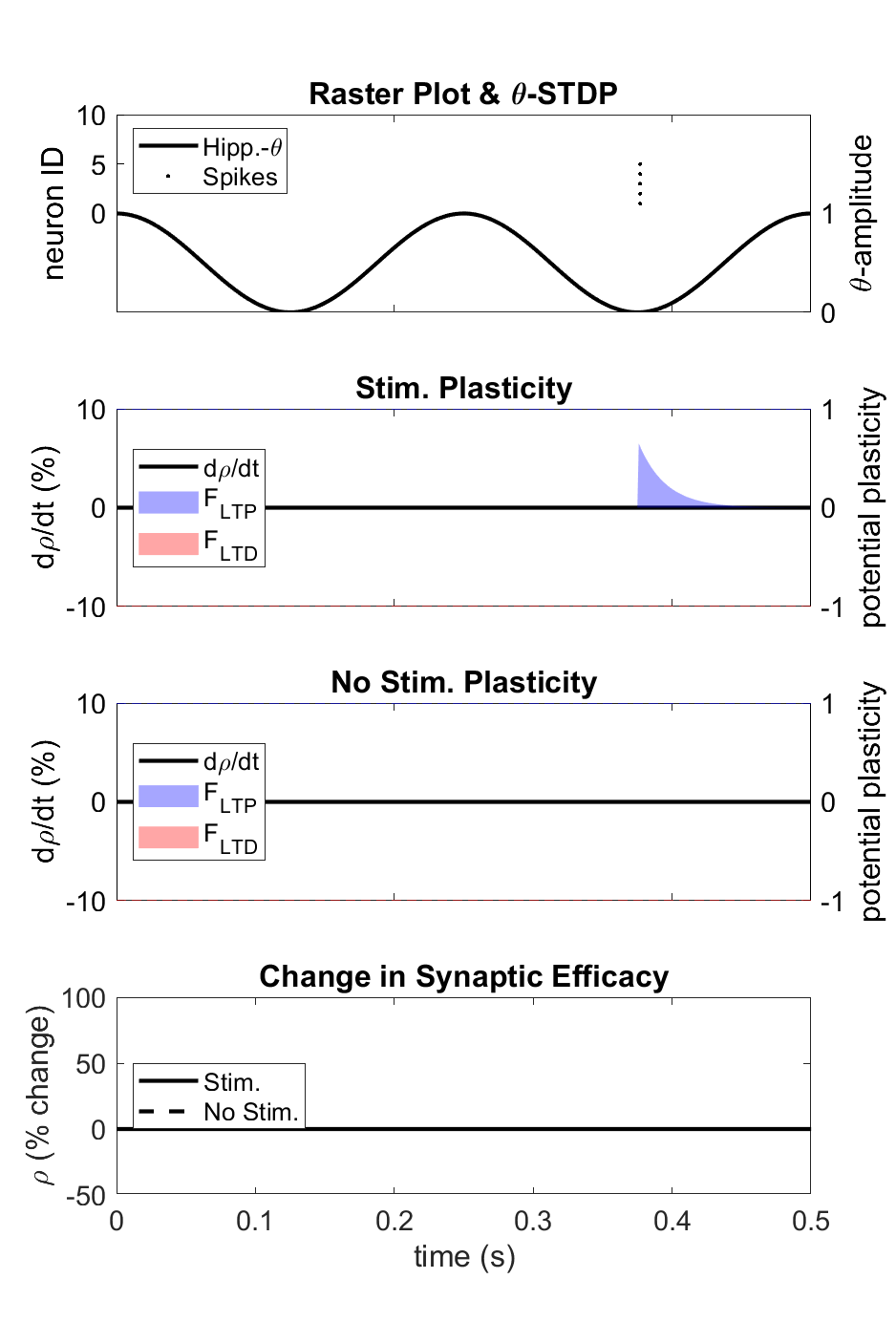

Supplement: Extended Data 1 — Human data and the MATLAB code of the model to run the simulations., Download [file enu-eN-NWR-0333-22-s07.zip › Sync-deSync-TIME-revision/HL_model/demo/het-STDP_single-burst_25T/weight_change_trough_1-shocks.tiff]

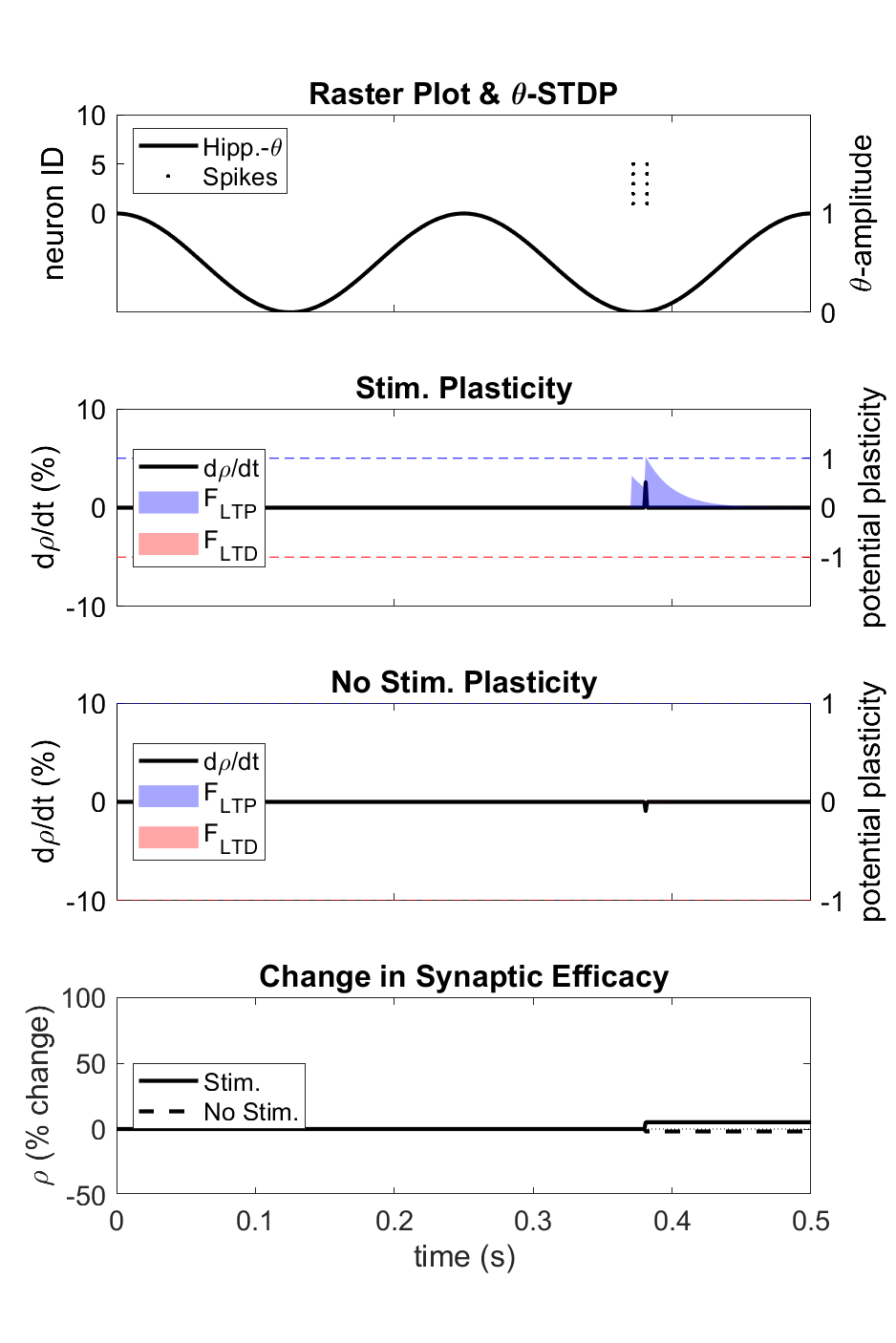

Supplement: Extended Data 1 — Human data and the MATLAB code of the model to run the simulations., Download [file enu-eN-NWR-0333-22-s07.zip › Sync-deSync-TIME-revision/HL_model/demo/het-STDP_single-burst_25T/weight_change_trough_2-shocks.tiff]

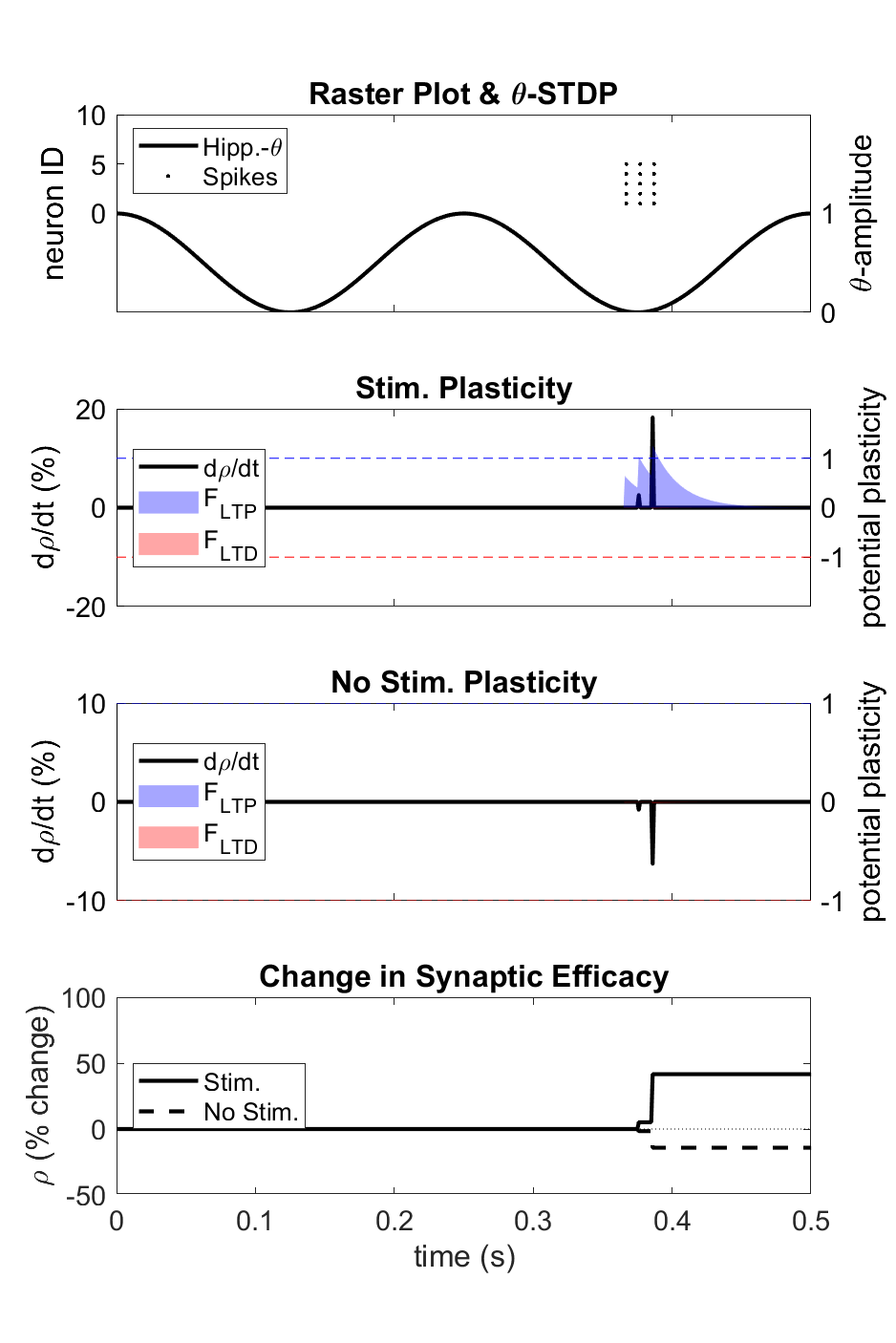

Supplement: Extended Data 1 — Human data and the MATLAB code of the model to run the simulations., Download [file enu-eN-NWR-0333-22-s07.zip › Sync-deSync-TIME-revision/HL_model/demo/het-STDP_single-burst_25T/weight_change_trough_3-shocks.tiff]

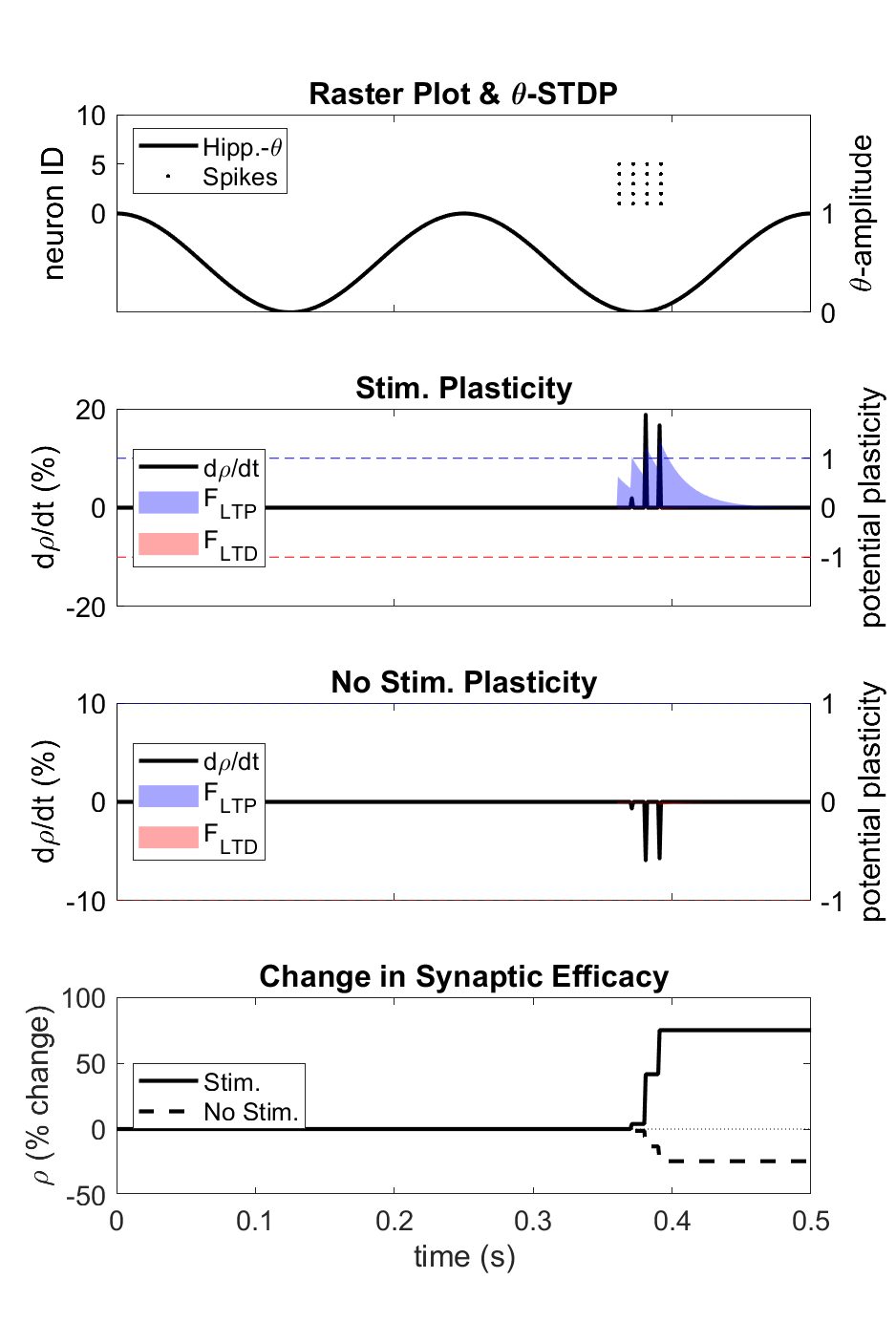

Supplement: Extended Data 1 — Human data and the MATLAB code of the model to run the simulations., Download [file enu-eN-NWR-0333-22-s07.zip › Sync-deSync-TIME-revision/HL_model/demo/het-STDP_single-burst_25T/weight_change_trough_4-shocks.tiff]

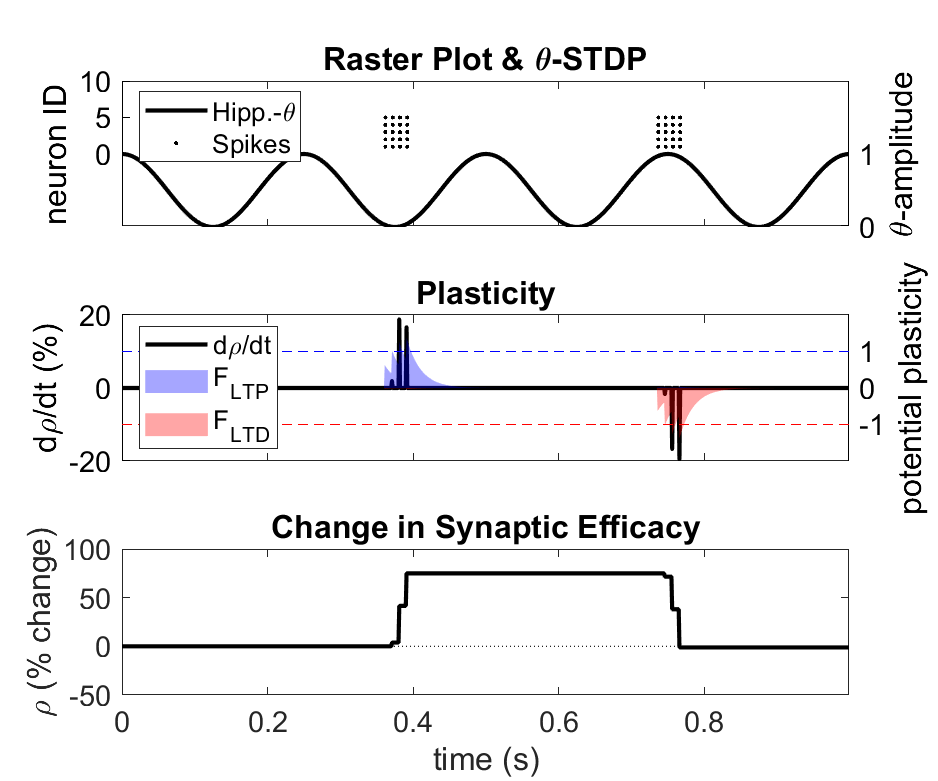

Supplement: Extended Data 1 — Human data and the MATLAB code of the model to run the simulations., Download [file enu-eN-NWR-0333-22-s07.zip › Sync-deSync-TIME-revision/HL_model/demo/multi-burst_100T/weight_change_both_4-shocks.tiff]

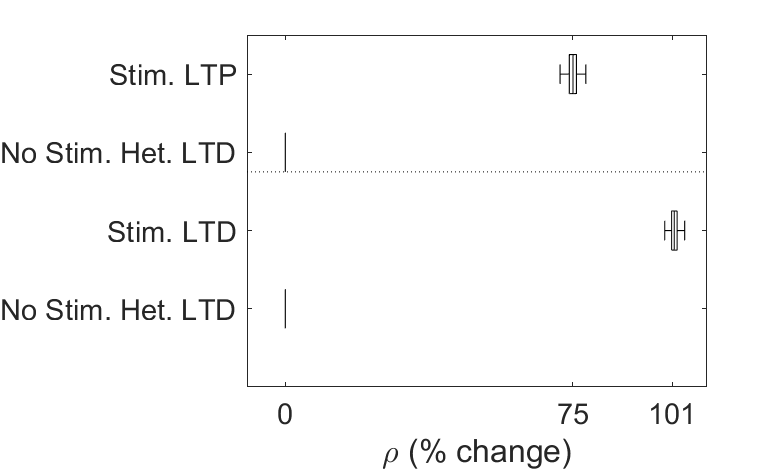

Supplement: Extended Data 1 — Human data and the MATLAB code of the model to run the simulations., Download [file enu-eN-NWR-0333-22-s07.zip › Sync-deSync-TIME-revision/HL_model/demo/multi-burst_100T/weight_change_theta_both_4-shocks.tiff]

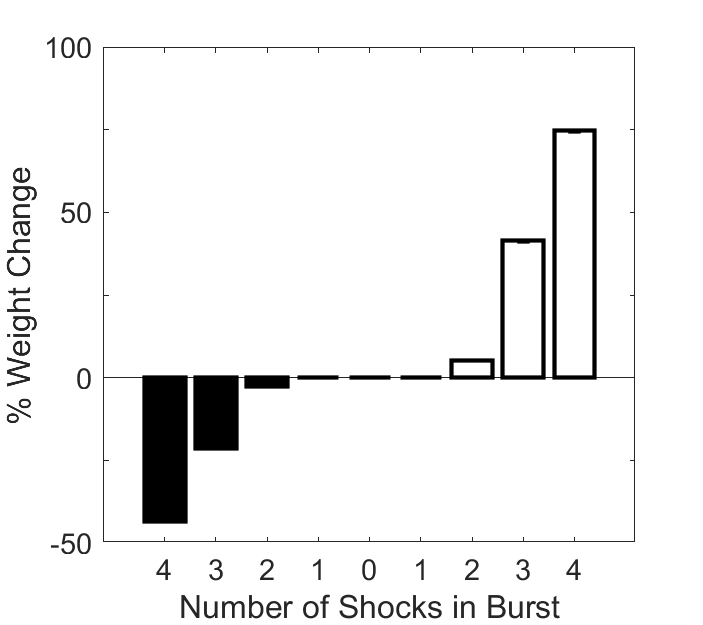

Supplement: Extended Data 1 — Human data and the MATLAB code of the model to run the simulations., Download [file enu-eN-NWR-0333-22-s07.zip › Sync-deSync-TIME-revision/HL_model/demo/single-burst_25T/weight_change.tiff]

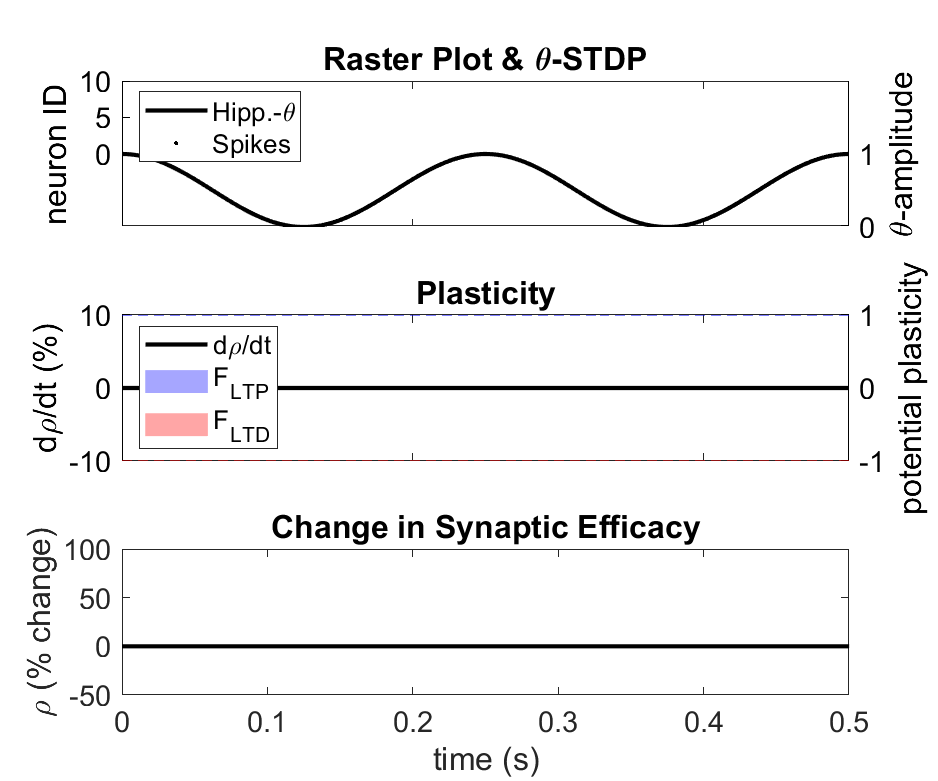

Supplement: Extended Data 1 — Human data and the MATLAB code of the model to run the simulations., Download [file enu-eN-NWR-0333-22-s07.zip › Sync-deSync-TIME-revision/HL_model/demo/single-burst_25T/weight_change_peak_0-shocks.tiff]

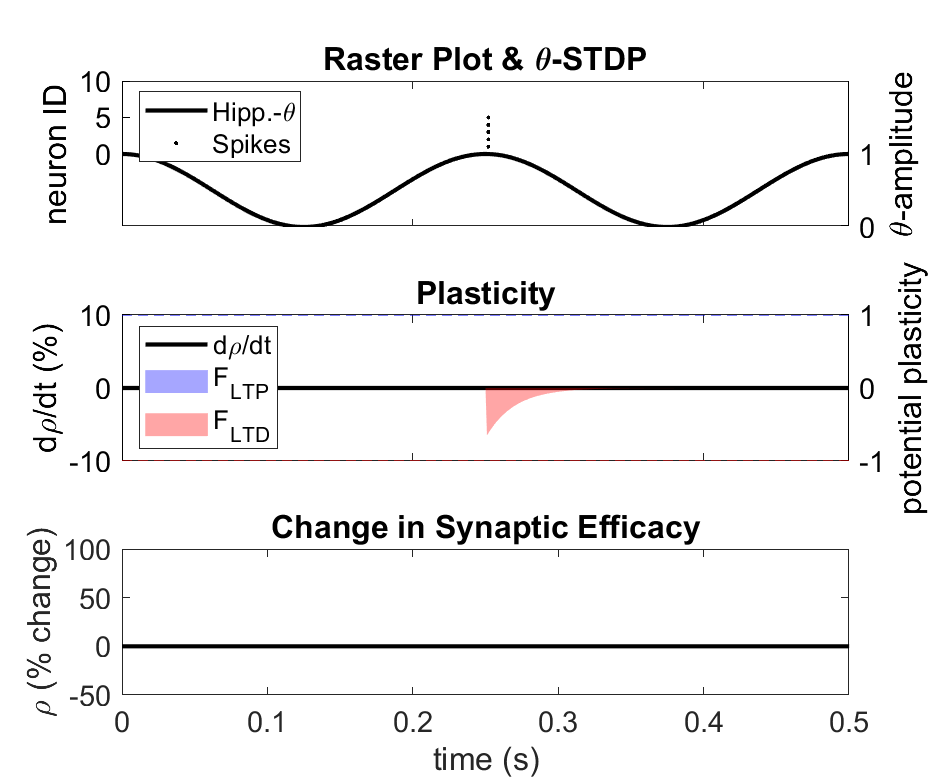

Supplement: Extended Data 1 — Human data and the MATLAB code of the model to run the simulations., Download [file enu-eN-NWR-0333-22-s07.zip › Sync-deSync-TIME-revision/HL_model/demo/single-burst_25T/weight_change_peak_1-shocks.tiff]

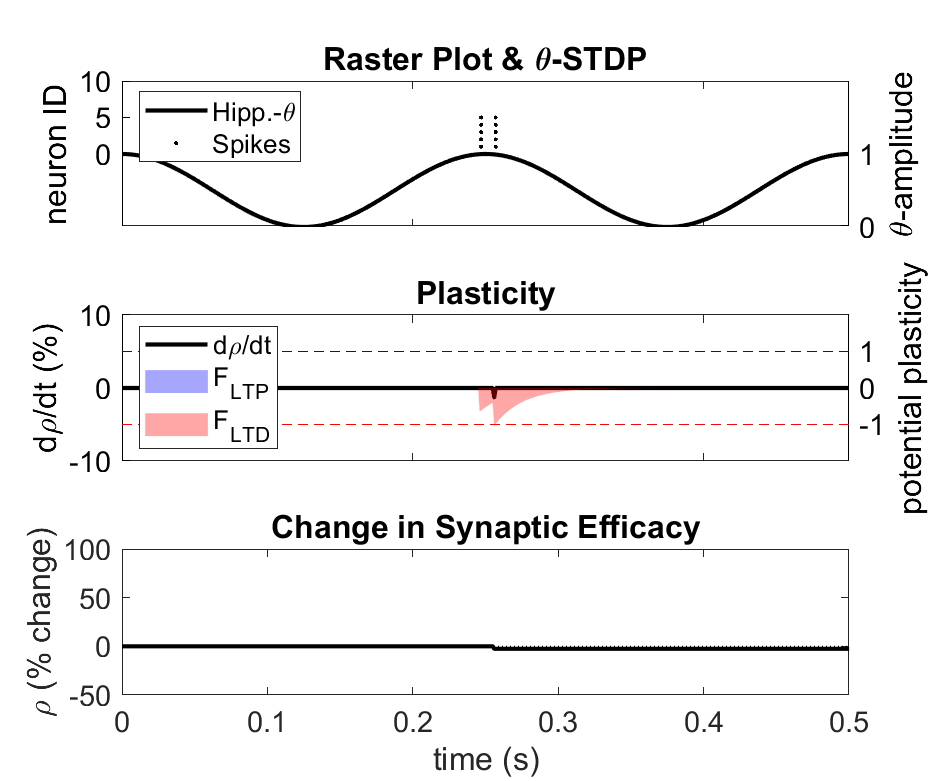

Supplement: Extended Data 1 — Human data and the MATLAB code of the model to run the simulations., Download [file enu-eN-NWR-0333-22-s07.zip › Sync-deSync-TIME-revision/HL_model/demo/single-burst_25T/weight_change_peak_2-shocks.tiff]

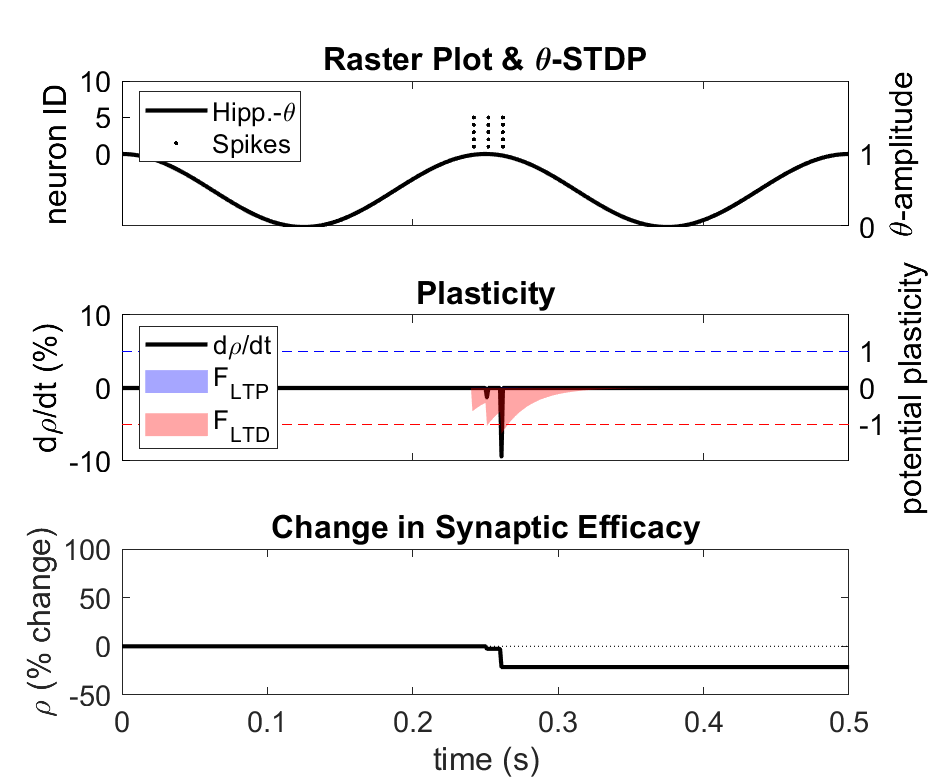

Supplement: Extended Data 1 — Human data and the MATLAB code of the model to run the simulations., Download [file enu-eN-NWR-0333-22-s07.zip › Sync-deSync-TIME-revision/HL_model/demo/single-burst_25T/weight_change_peak_3-shocks.tiff]

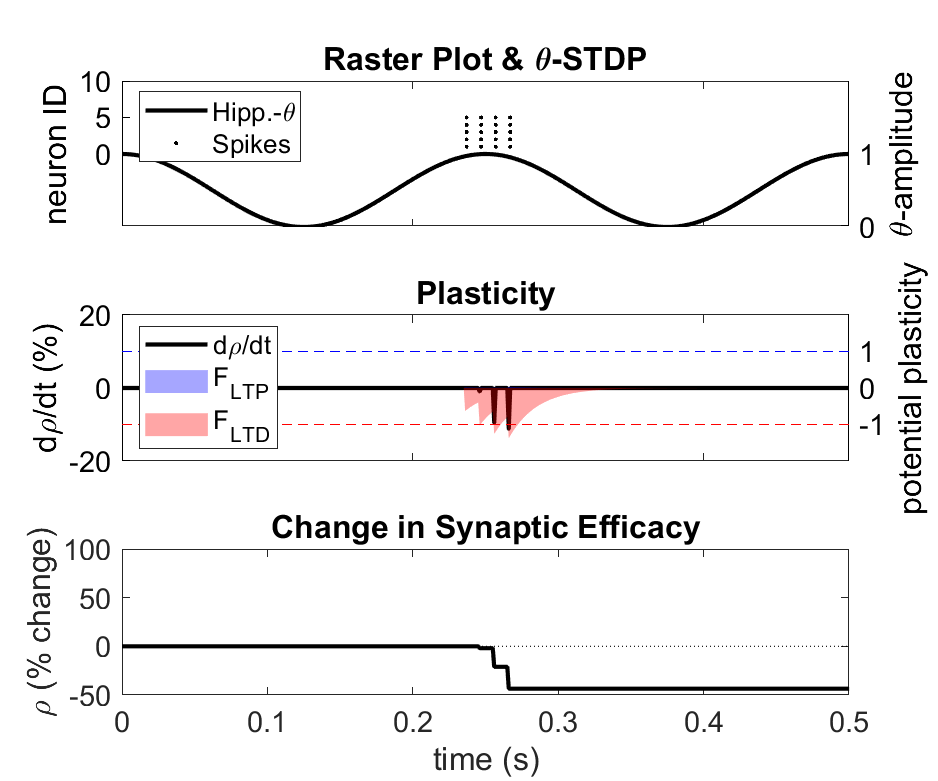

Supplement: Extended Data 1 — Human data and the MATLAB code of the model to run the simulations., Download [file enu-eN-NWR-0333-22-s07.zip › Sync-deSync-TIME-revision/HL_model/demo/single-burst_25T/weight_change_peak_4-shocks.tiff]

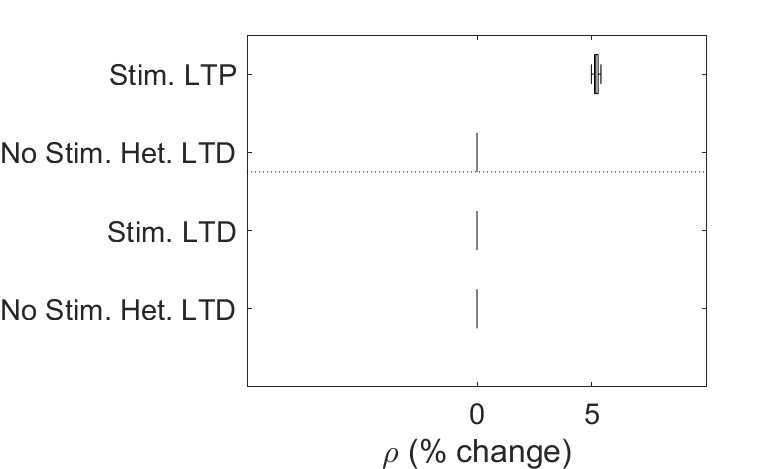

Supplement: Extended Data 1 — Human data and the MATLAB code of the model to run the simulations., Download [file enu-eN-NWR-0333-22-s07.zip › Sync-deSync-TIME-revision/HL_model/demo/single-burst_25T/weight_change_theta_trough_2-shocks.tiff]

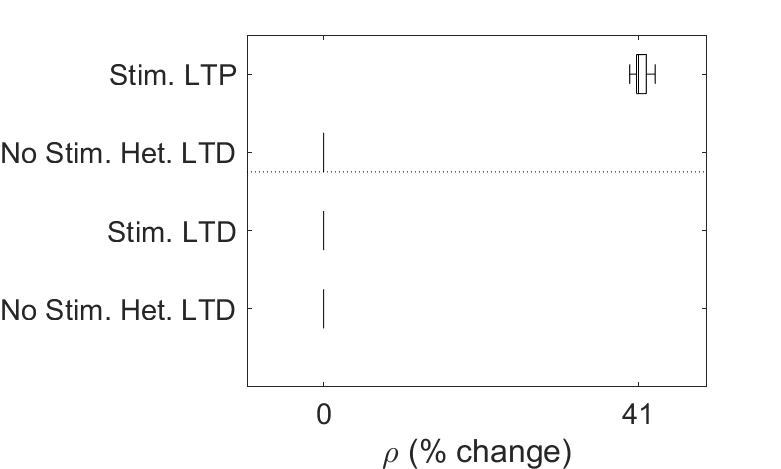

Supplement: Extended Data 1 — Human data and the MATLAB code of the model to run the simulations., Download [file enu-eN-NWR-0333-22-s07.zip › Sync-deSync-TIME-revision/HL_model/demo/single-burst_25T/weight_change_theta_trough_3-shocks.tiff]

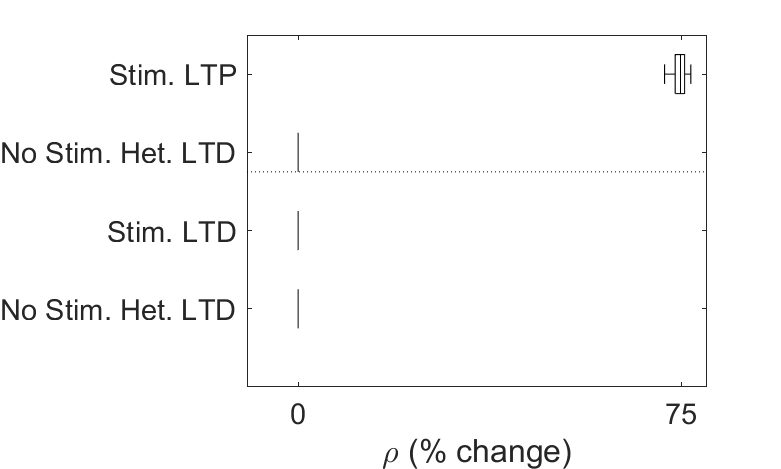

Supplement: Extended Data 1 — Human data and the MATLAB code of the model to run the simulations., Download [file enu-eN-NWR-0333-22-s07.zip › Sync-deSync-TIME-revision/HL_model/demo/single-burst_25T/weight_change_theta_trough_4-shocks.tiff]

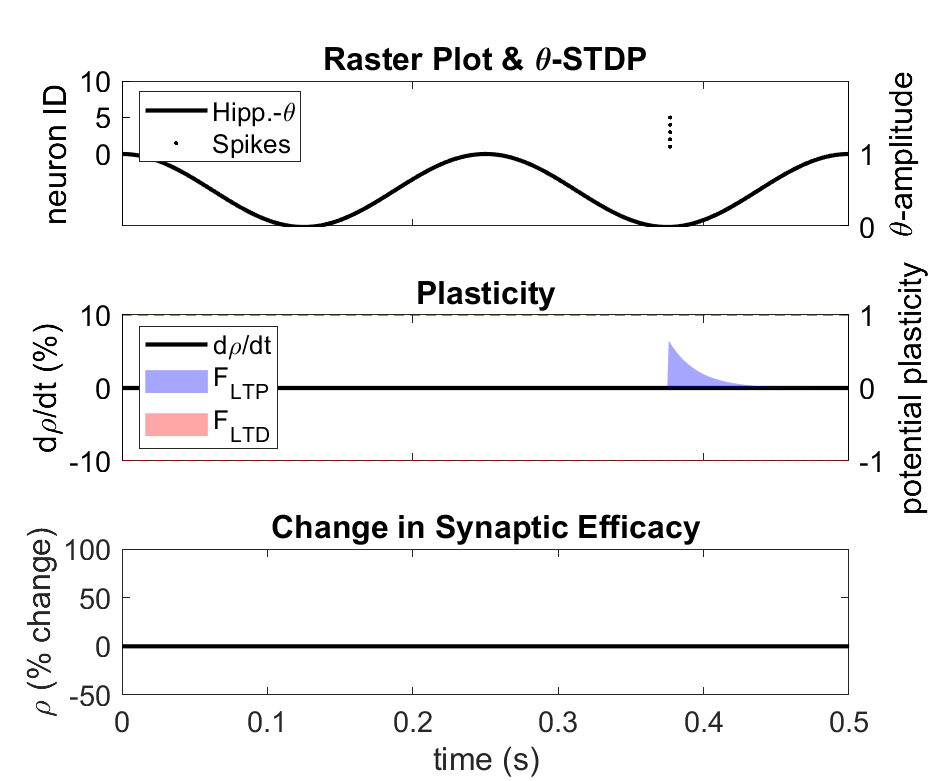

Supplement: Extended Data 1 — Human data and the MATLAB code of the model to run the simulations., Download [file enu-eN-NWR-0333-22-s07.zip › Sync-deSync-TIME-revision/HL_model/demo/single-burst_25T/weight_change_trough_1-shocks.tiff]

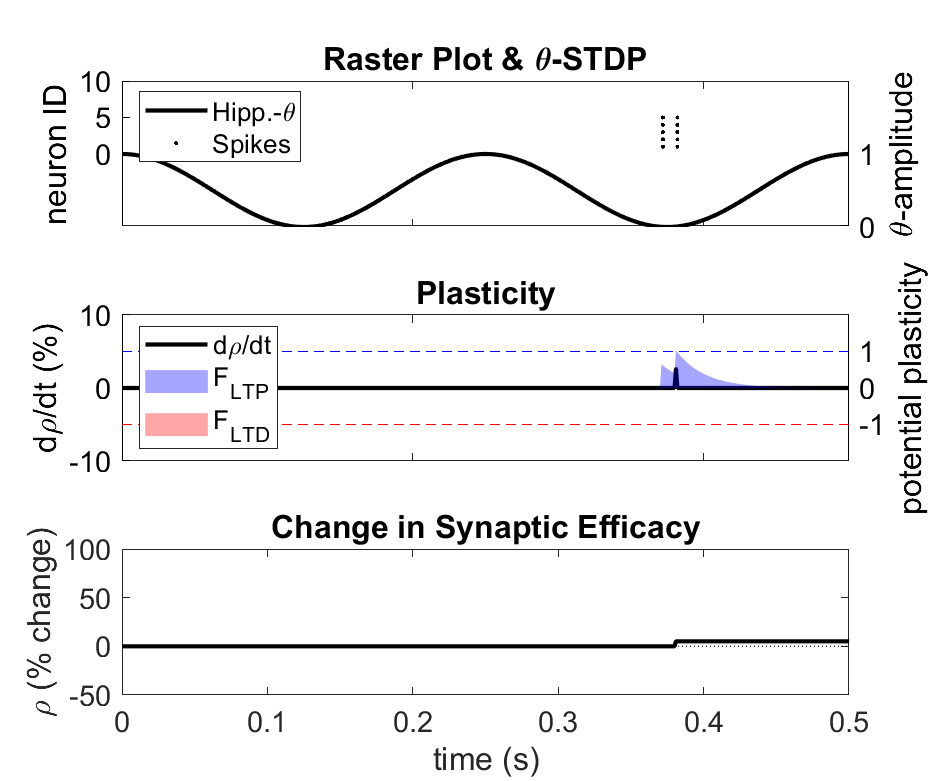

Supplement: Extended Data 1 — Human data and the MATLAB code of the model to run the simulations., Download [file enu-eN-NWR-0333-22-s07.zip › Sync-deSync-TIME-revision/HL_model/demo/single-burst_25T/weight_change_trough_2-shocks.tiff]

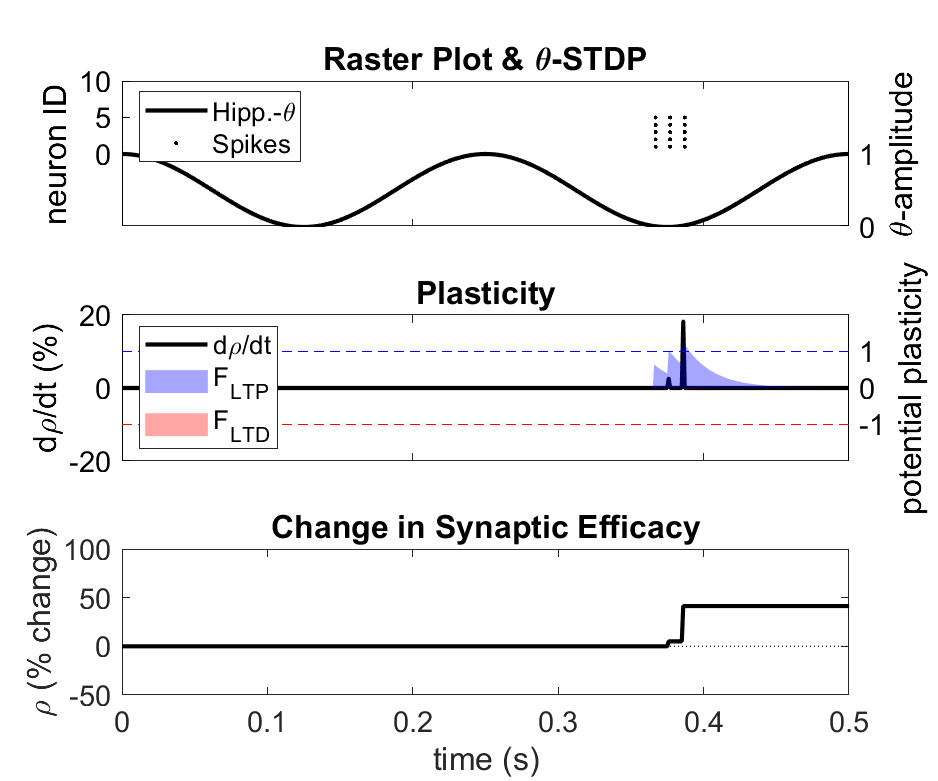

Supplement: Extended Data 1 — Human data and the MATLAB code of the model to run the simulations., Download [file enu-eN-NWR-0333-22-s07.zip › Sync-deSync-TIME-revision/HL_model/demo/single-burst_25T/weight_change_trough_3-shocks.tiff]

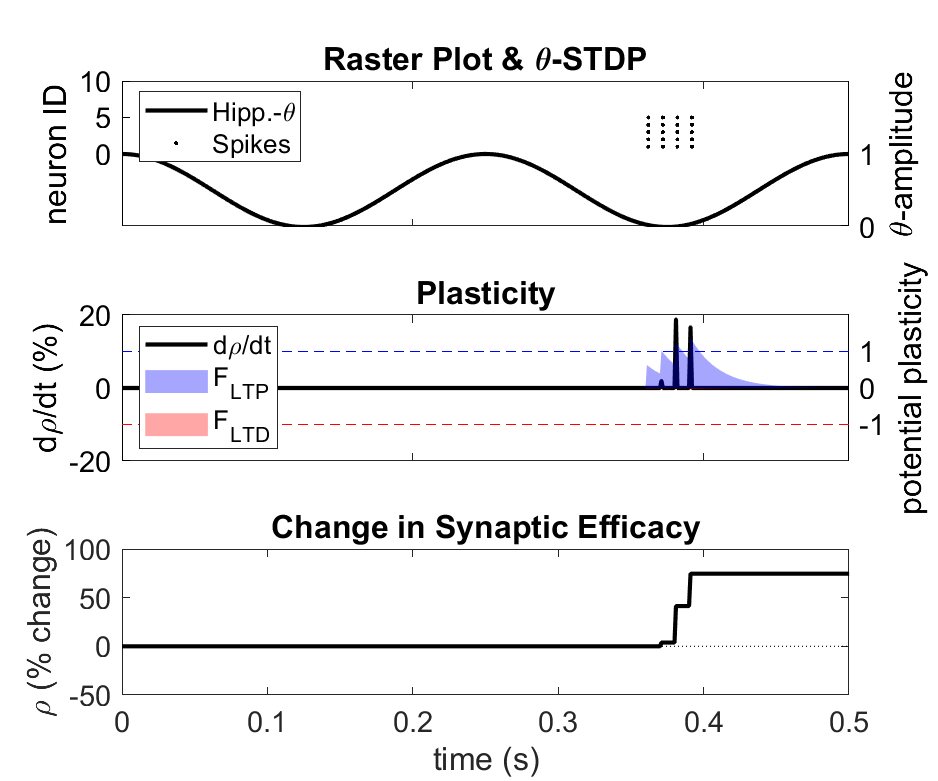

Supplement: Extended Data 1 — Human data and the MATLAB code of the model to run the simulations., Download [file enu-eN-NWR-0333-22-s07.zip › Sync-deSync-TIME-revision/HL_model/demo/single-burst_25T/weight_change_trough_4-shocks.tiff]
